# Revealing the Mechanism of TEMPO-Hypervalent Iodine(III) Oxidation of Alcohols

**DOI:** 10.1021/jacs.5c21609

**Published:** 2026-02-19

**Authors:** Michael Bingham, Tapas R. Pradhan, Dhananjay Bhattacherjee, Rawiyah Alkahtani, Paul Kavanagh, Thomas Wirth, Paul Dingwall

**Affiliations:** † School of Chemistry and Chemical Engineering, 1596 Queen’s University Belfast, BT7 1NN Belfast, Northern Ireland; ‡ School of Chemistry, 2112 Cardiff University, Main Building, CF10 3AT Cardiff, Cymru/Wales

## Abstract

Experimental and computational studies on the mechanism of a well-known procedure for the oxidation of alcohols to carbonyl compounds using TEMPO and the hypervalent iodine­(III) reagent (diacetoxyiodo)­benzene (PIDA) are reported. Kinetic data show that the assumed classical oxoammonium–hydroxylamine mechanism requires modification due to zero-order behavior observed in TEMPO. Instead, a dual catalytic system is proposed featuring two rate-determining steps involving a combination of alcohol, hypervalent iodine species, and water, which is typically present in adventitious quantities and is necessary for the reaction to proceed. The use of different alcohols implies the mechanism to be general. Intramolecular radical trap probes rule out a radical mechanism, while an investigation of TEMPO derivatives suggests that TEMPO is involved prior to the rate-determining step. Kinetic isotope effect studies demonstrate that TEMPO is also involved after the rate-determining step. Electrochemical studies find that the oxoammonium form of TEMPO is reduced by PIDA, likely oxidizing iodine­(III) to an iodine­(V) species. Theoretical investigations support the feasibility of a pathway involving an iodine­(V) species, demonstrate good agreement with the experimentally derived kinetics, and support an updated mechanism. Finally, the demonstration of oxidative kinetic resolution of a secondary alcohol using a chiral iodine­(III) reagent rationally extends the reactivity of this system to new chemistry and lends further support toward our mechanistic proposals.

## Graphical abstract

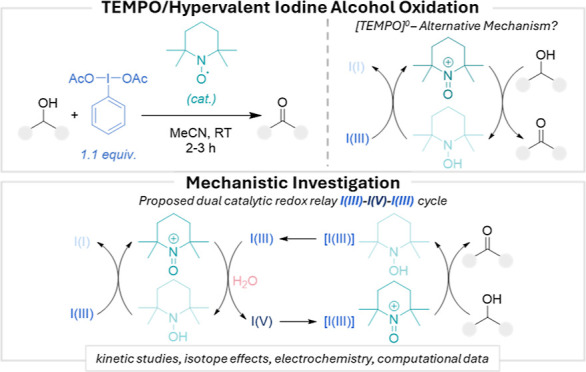

## Introduction

The oxidation of alcohols to aldehydes or ketones is a basic transformation in organic chemistry. Nitroxides or aminoxyl radicals, such as 2,2,6,6-tetramethylpiperidin-1-oxyl (TEMPO), are well-known catalysts for alcohol oxidation and have been subject to numerous studies and reviews.^1–4^ TEMPO-mediated oxidations fall into several distinct modes of activity. The most common is via an oxoammonium/hydroxylamine pathway (Scheme 1A).^5–7^ Here, the active oxidant is the oxoammonium species, either generated in situ from catalytic TEMPO by a terminal oxidant or used stoichiometrically via a stable form.^8,9^ The identity of the terminal oxidant can be wide ranging, from NaOCl,^10,11^ Oxone,^12^ an electrode,^13^ or cocatalytic aerobic systems.^14–16^

**Scheme 1:**
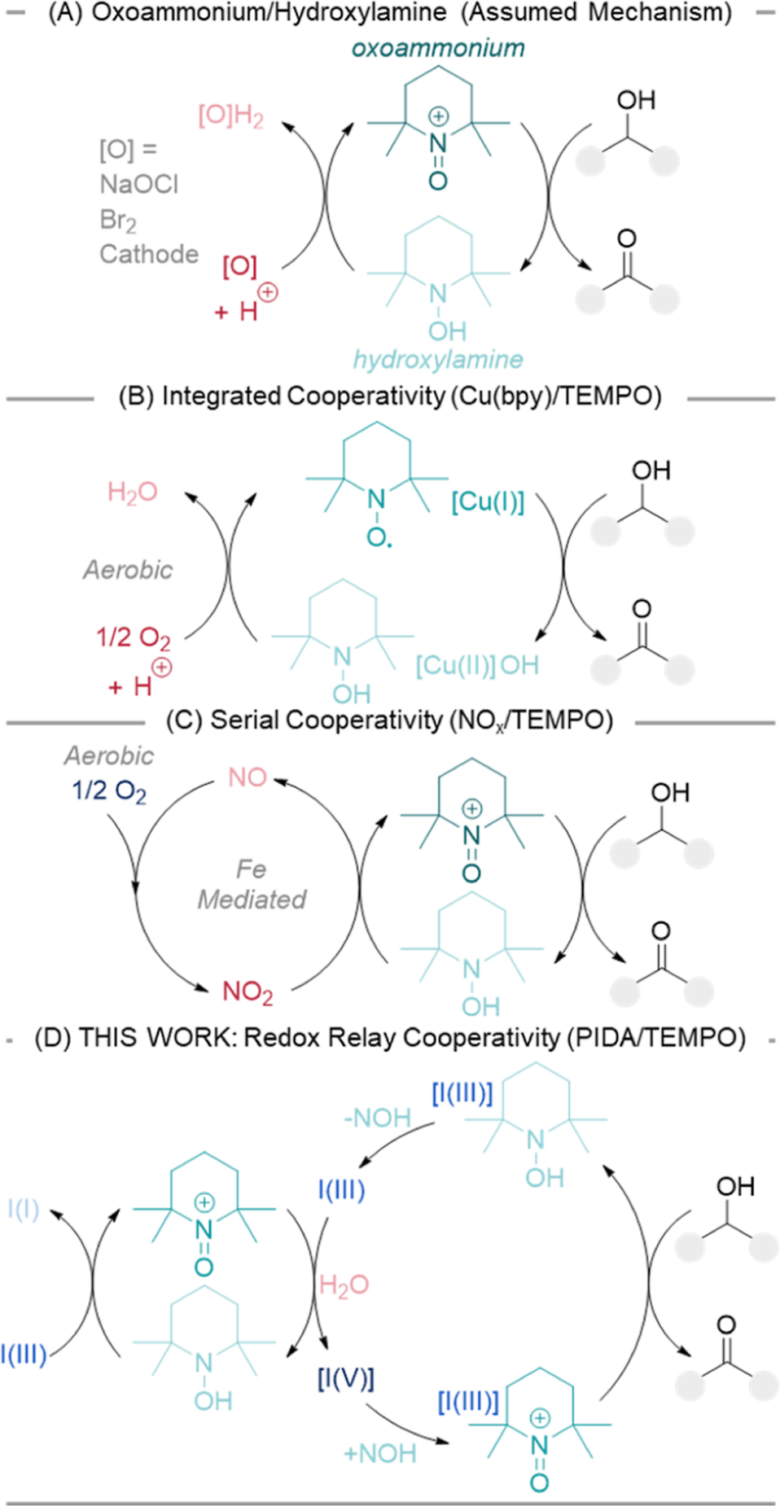
Mechanisms of TEMPO Alcohol Oxidation: (A) Oxoammonium/Hydroxylamine Direct Oxidizing Agent;^5–7^ (B) Integrated Cooperativity;^17–21^ (C) Serial Cooperativity;^14^ (D) This Work: Redox Relay Cooperativity. Reproduced from ref 14. Copyright 2021 American Chemical Society

TEMPO has also been shown to act as a cocatalyst, promoting alcohol oxidation through cooperativity rather than directly through an active oxoammonium, the most prominent example being Stahl’s seminal mechanistic work on an aerobic Cu­(bpy)/TEMPO system (Scheme 1B)^17–21^ and, recently, a NO_*x*_/TEMPO system (Scheme 1C).^14^

A very popular terminal oxidant for TEMPO-mediated alcohol oxidations is (diacetoxyiodo)­benzene (PIDA), developed by Piancatelli and co-workers in 1997 (Scheme 2).^22^ While hypervalent iodine­(V) reagents have their own rich history as alcohol oxidants, there are significantly fewer examples of alcohol oxidation involving iodine­(III) reagents.^23,24^

**Scheme 2:**
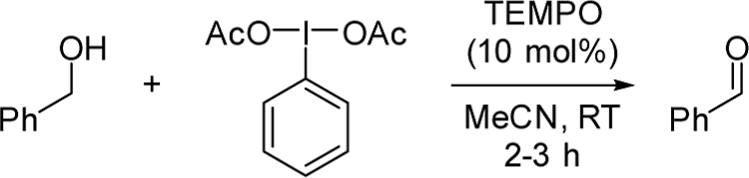
Modification of Piancatelli and Co-Worker’s TEMPO/PIDA-Mediated Oxidation of Benzyl Alcohol to Benzaldehyde Used as a Model Reaction in This Study^a^

a [BnOH] = 0.16M, [PIDA] = 0.176M.^22^

TEMPO/PIDA oxidation to aldehydes proceeds rapidly under mild conditions with no need for exclusion of air or water, is exceptionally functional group-tolerant, can oxidize a wide range of structurally diverse alcohols, is highly selective toward primary alcohols, and displays no overoxidation to carboxylic acids. Since inception, the role of PIDA has been assumed simply as that of a terminal oxidant, with the mechanism supposedly proceeding via an oxoammonium/hydroxylamine cycle as shown in Scheme 1A.^1,22,25^

However, there are several inconsistencies displayed by the TEMPO/PIDA system that do not fit expected mechanistic behavior: (1) oxoammonium systems favor the oxidation of primary over secondary alcohols under basic conditions and vice versa under acidic conditions.^5,26^ Yet TEMPO/PIDA, with the release of two equivalents of acetic acid, oxidizes primary alcohols exclusively in the presence of secondary alcohols. (2) TEMPO/PIDA cleaves 1,2-diols, whereas oxoammonium systems produce dicarbonyl compounds.^26^ (3) The presence of an organic base dramatically decreases the reaction rate of TEMPO/PIDA, whereas a base typically increases the rate of oxoammonium-mediated oxidations due to turnover-limiting hydride transfer.^2,26^

Herein, we report a full reappraisal of the TEMPO/PIDA reaction mechanism, including kinetic, electrochemical, and computational studies. We demonstrate that the hydroxylamine/oxoammonium pathway of Scheme 1A is unlikely to be in operation without significant modification and, instead, propose an updated mechanism (Scheme 1D) for redox relay cooperativity. We show the mechanistically important role of water and, while TEMPO does ultimately oxidize the alcohol, PIDA unusually acts as both a terminal oxidant and a cocatalyst in a dual catalytic cycle while accessing the iodine­(V) oxidation state.

## Results and Discussion

### Kinetic Studies

Kinetic studies employing the variable time normalization analysis (VTNA) of Burés were conducted.^27^ Modifications were made to Piancatelli and co-worker’s original reaction conditions to ensure a homogeneous reaction mixture (Scheme 2, see Table S1 of the Supporting Information).^22^ While the reaction was found to be first order in PIDA, partially positive order in alcohol, and zero order in the acetic acid byproduct, the first surprising result was the finding of zero-order behavior in TEMPO (Figure 1 and Figure S1 of the Supporting Information). As the rate does not change when [TEMPO] is modified, this result effectively calls into question the long-assumed mechanism shown in Scheme 1A, where TEMPO is present in each step of the catalytic cycle.

**Figure 1:**
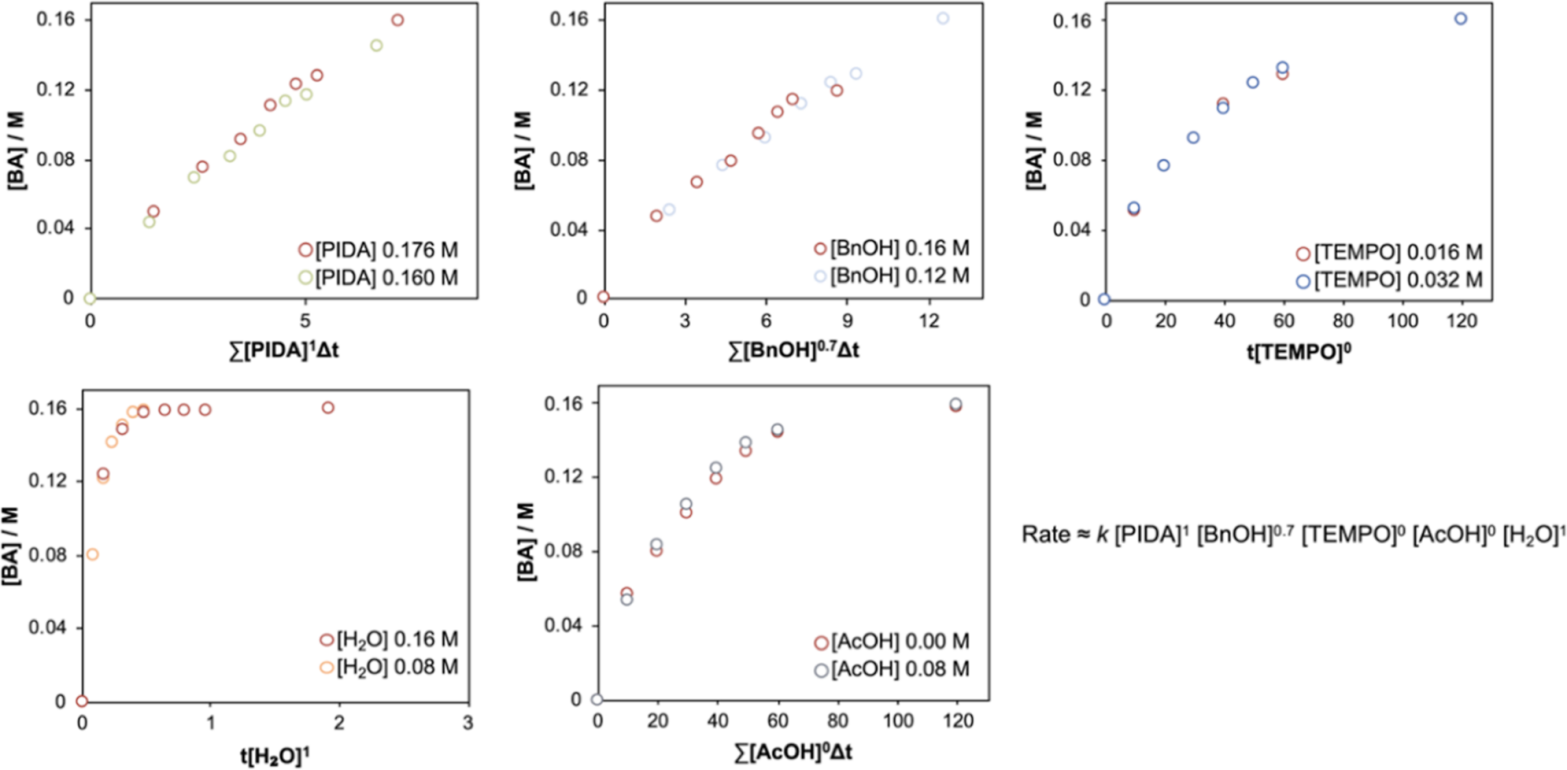
VTNA studies indicating the reaction order for each compound. Concentration ranges, with bold indicating standard conditions: [BnOH] = 0.12–**0.16** M, [PIDA] = 0.16–**0.176** M, [TEMPO] = **1.6**–3.2 mM, [AcOH] = **0**–0.08M, [H_2_O] = **0**–0.16 M MeCN, RT.

The second surprising result was the finding that the reaction is first order in water.^25^ Our experiments show that water is essential for the reaction to proceed (see Figure S3 in the Supporting Information) and that adventitious water is enough for the reaction to proceed. Further experimentation shows that the reaction does not saturate under increasing addition of water, even above the concentration of PIDA, and that a small inverse kinetic isotope effect is observed on the addition of D_2_O over H_2_O (Figure 2). Mechanistically, this lack of saturation in water rules out a scenario in which water acts as an off-cycle activator to create an on-cycle catalyst (see Scheme S1 in the Supporting Information), suggesting that water plays a direct role in the catalytic cycle.

**Figure 2:**
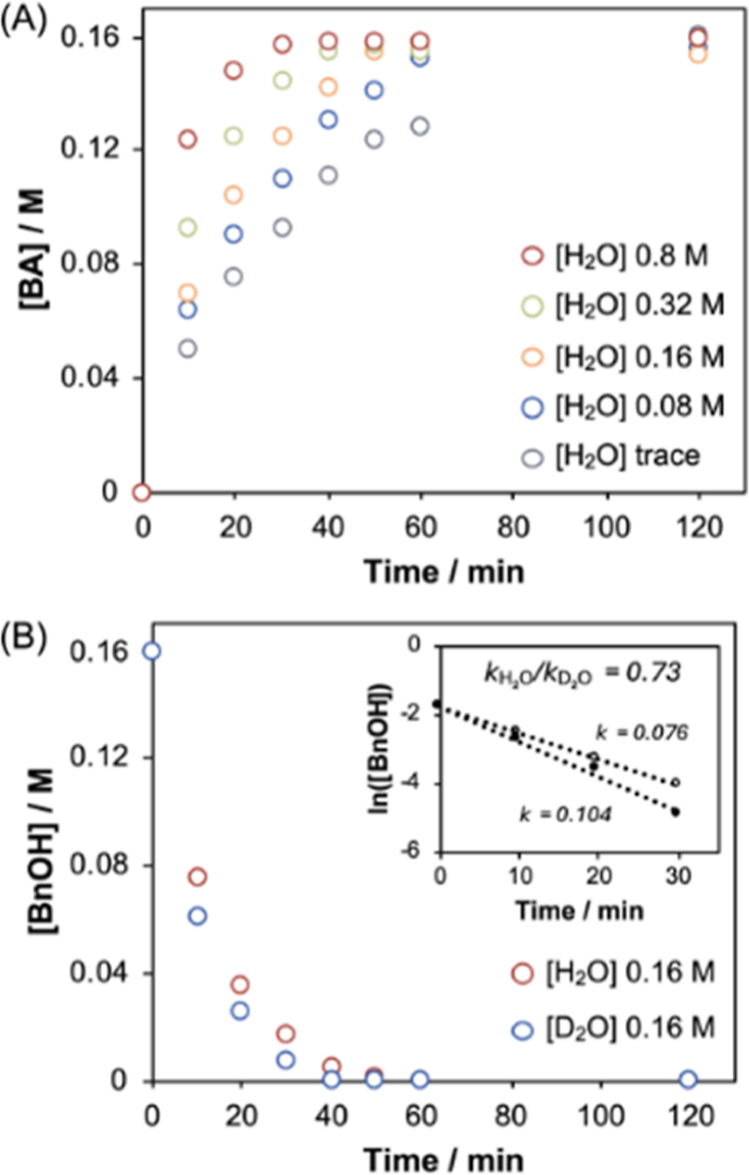
(A) Time course data showing continuous change in reaction rate for increasing concentrations of H_2_O; no saturation behavior is observed; (B) time course data showing an increased rate of reaction for H_2_O and D_2_O. (B, inset) A plot of ln­[BnOH] against time showing an inverse kinetic isotope effect for H_2_O/D_2_O. These experiments have been carried out in triplicate with an error of ca. 5%.

Considering the number of exchangeable protons in the reaction system, the observation of an inverse kinetic isotope effect is more challenging to explain. However, this does indicate the importance of deuterium substitution in the rate-determining step (Figure 2B, inset).

### Comparing Alcohol Reactivity

We thought we would examine the kinetic behavior of different alcohols to probe the generality of the mechanism. Further kinetic studies for model primary and secondary alcohols, both aromatic and aliphatic, were performed (Figures 3; see Figure S5 in the Supporting Information).

**Figure 3:**
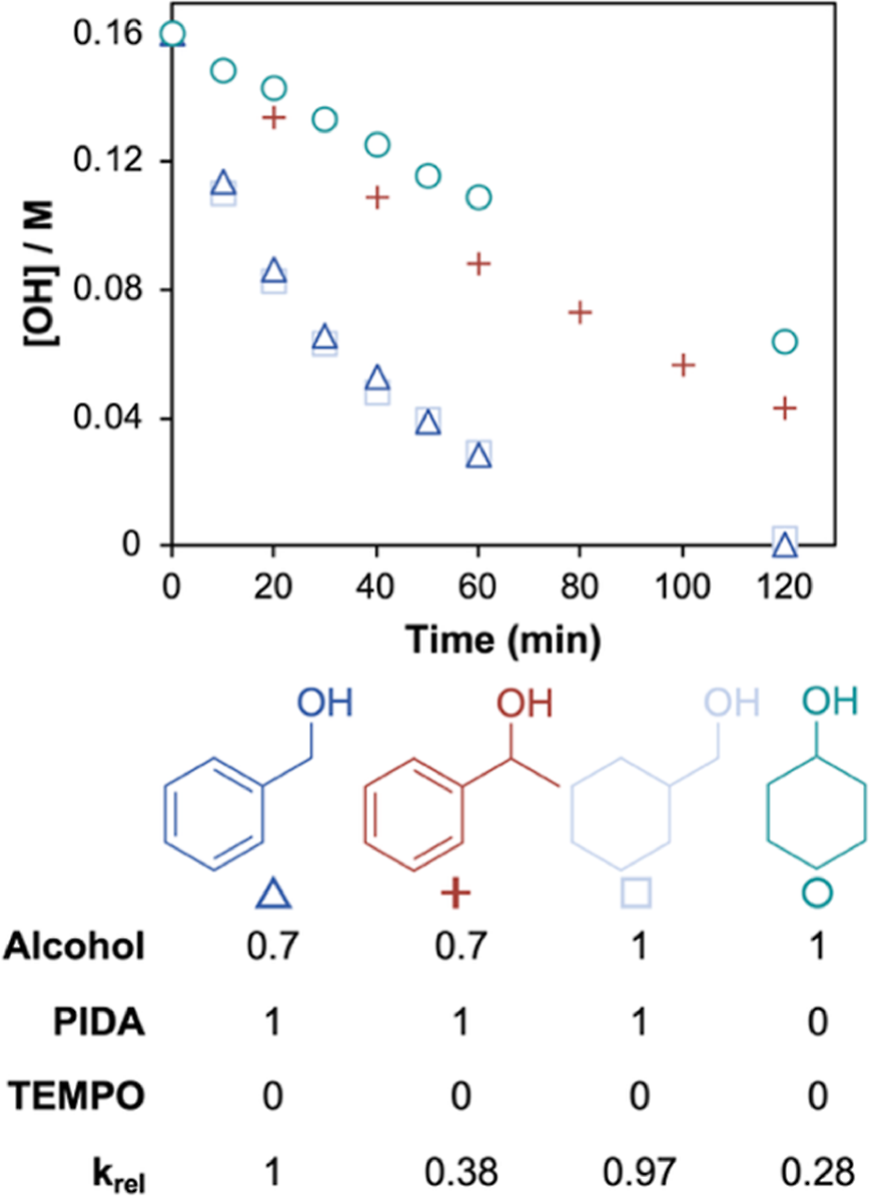
Comparing the behavior of different alcohols: absolute rates and kinetic orders. [OH] = 0.16 M, [PIDA] = 0.176 M, [TEMPO] = 1.6 mM, MeCN, RT. The relative rate, *k*_rel_, is given as *k*_obv_ (ROH)/*k*_obv_(BnOH).

Benzyl alcohol and cyclohexyl methanol displayed almost identical rate behavior and similar rate laws. 1-Phenylethanol displayed a rate law identical to that of benzyl alcohol but was considerably slower. Similarities between rate laws suggest that these alcohols operate by the same general mechanism. Cyclohexanol was slower still and displayed a different rate law, with PIDA now zero order, suggesting the oxidation of secondary aliphatic alcohols may proceed by a different mechanism (Figure S5 in the Supporting Information).

A Hammett study was conducted by varying the electronic properties of *para*-substituted benzyl alcohol derivatives. Comparison of the standard reaction conditions, in which the concentration of PIDA is noticeably changing over time due to its small excess (1.1 equiv), reveals a Hammett relationship of small magnitude (Figure 4A).

**Figure 4:**
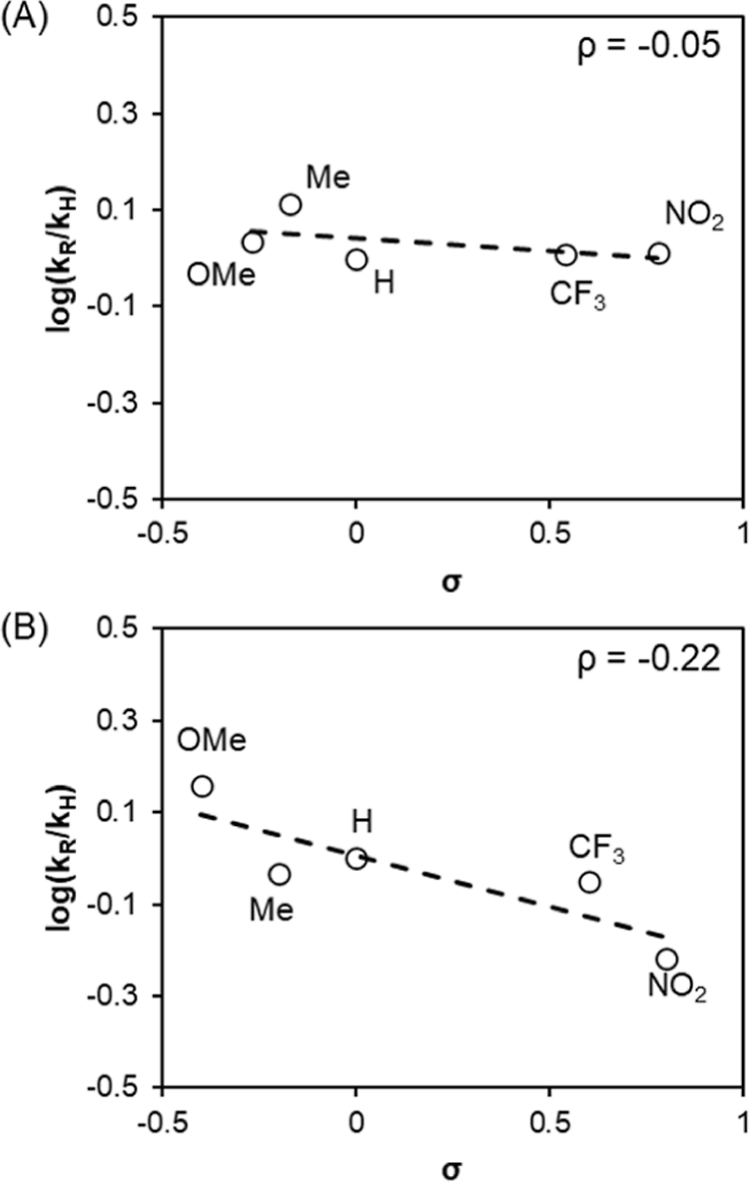
Hammett plots constructed from independent rate measurements at the following: (A) standard: [PIDA] = 0.176 M, [BnOH] = 0.16 M, [TEMPO] = 0.016 M; (B) flooding: [PIDA] = 0.2 M, [BnOH] = 0.04 M, [TEMPO] = 4 mM.

A more pronounced ρ value is observed when a significantly larger excess of PIDA (5 equiv) is used (Figure 4B). Identical behavior was observed for both independent and competition reactions. The more rapid oxidation of electron-rich alcohols demonstrates that a less acidic alcohol proton is more favorable for the reaction to proceed. The mechanistic origin of this complex behavior, which we believe also ties in with the inverse KIE observed with D_2_O (Figure 2B), will be discussed below.

### Radical Trapping and TEMPO Derivatives

With the involvement of TEMPO, it is possible that the reaction mechanism is single electron in nature. Attempts to monitor the reaction by EPR were unsuccessful. Nitroxide radicals like TEMPO are commonly used in EPR studies as spin labels and single-electron probes.^2,28^ Due to the high stability of the unpaired electron, only the TEMPO signal was observable, overshadowing any potential observations in the system.

TEMPO is also routinely used as a radical trap to probe whether a reaction is single electron in nature;^29^ this ruled out the addition of ex situ radical traps to probe the presence of radicals in the mechanism. Instead, we employed an intramolecular radical trap on the alcohol,^30,31^ with the assumption that cyclization would be faster than intermolecular radical trapping, especially as only catalytic quantities of TEMPO are present. Only the expected aldehyde was observed in excellent yield, suggesting radical formation at the benzylic position is unlikely (Scheme 3).^32–36^

**Scheme 3:**
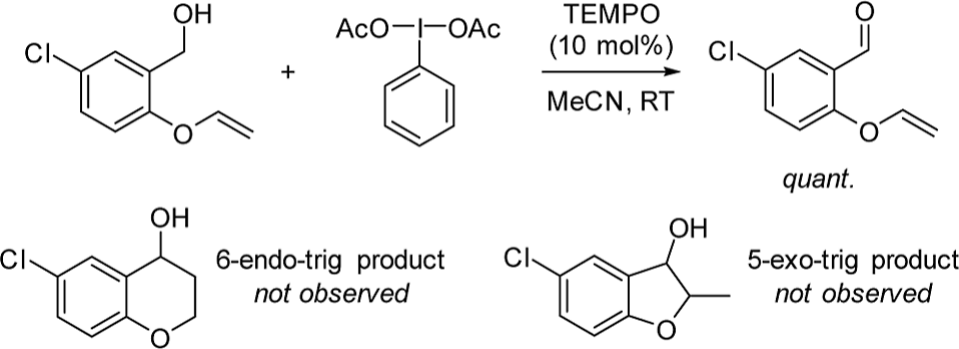
Radical Trap Experiment Observes Only Conversion to Aldehyde, Ruling Out Formation of a Radical at the Benzylic Position^a^

a [Alcohol] = 0.16M, [PIDA] = 0.176M.

There are a range of TEMPO derivatives available, each with different redox properties as well as steric profiles,^2^ either of which can alter mechanisms and also allow tuning of selectivity.^37^ To our surprise, the rate of the reaction changed (Figure 5A), correlating broadly with the redox potential (Figure 5B). This observation suggests that TEMPO must be involved in the mechanism prior to the rate-determining step. An obvious outlier is 4-CyanoTEMPO, which shows a higher rate than predicted by the simple correlation. This may arise from additional substituent effects such as solvation or association of the oxoammonium intermediate, indicating that factors beyond redox potential alone can influence the observed kinetics.^2^

**Figure 5:**
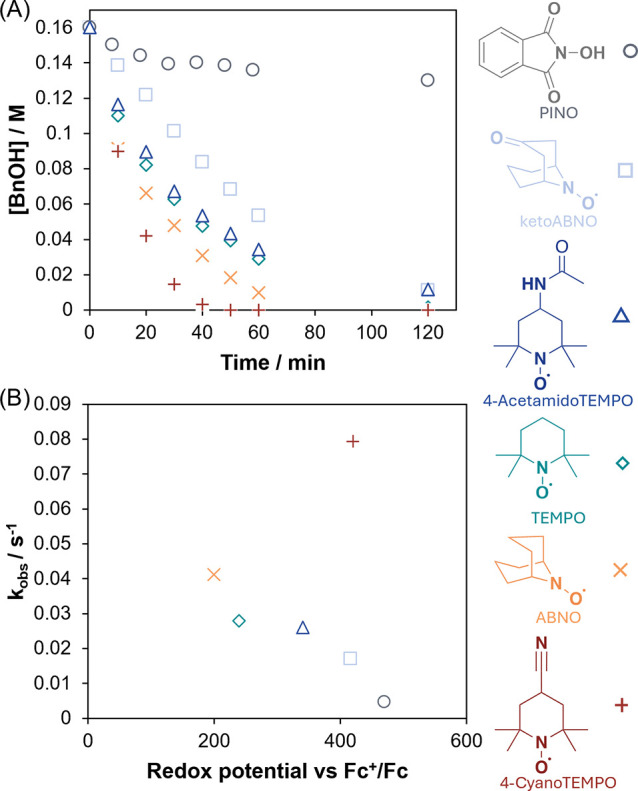
(A) The consumption of benzyl alcohol as a function of time for various nitroxyl species; (B) *k*_obs_ (initial rate) for each nitroxyl species as a function of redox potential.

Nitroxyl radicals are often part of complex one- and two-electron redox equilibria with many different species,^1,2^ and it can be difficult to determine which species are involved in a particular mechanism.^38^ The exceedingly slow reaction with *N*-hydroxyphthalimide (NHPI) as a nitroxyl radical precursor suggests the central importance of the oxoammonium form of TEMPO in the reaction mechanism. PIDA is known to generate the radical PINO from NHPI.^39^ However, PINO cannot access an oxoammonium form, and we attribute the poor reactivity to the inability to access the oxoammonium (Scheme 4).^31^ We also suggest this is additional evidence against a radical mechanism, as PINO is significantly more proficient at H atom transfer than TEMPO due to the high energy of the NO–H bond.^40^

**Scheme 4:**
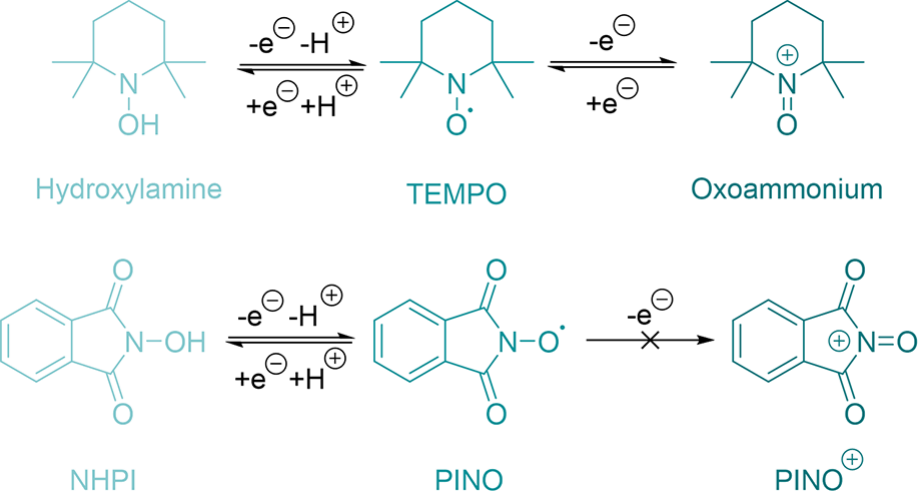
One- and Two-Electron Redox Pathways of TEMPO, to Access the Hydroxylamine and Oxoammonium, and NHPI to PINO

### Kinetic Isotope Effects

Kinetic isotope effects were studied to gain further insight into the nature of the rate-determining step. Independent rate measurements of the kinetic isotope effect of α,α-d_2_ benzyl alcohol showed no discernible KIE (Figure 6A), indicating that alcohol oxidation is not rate-determining.

**Figure 6:**
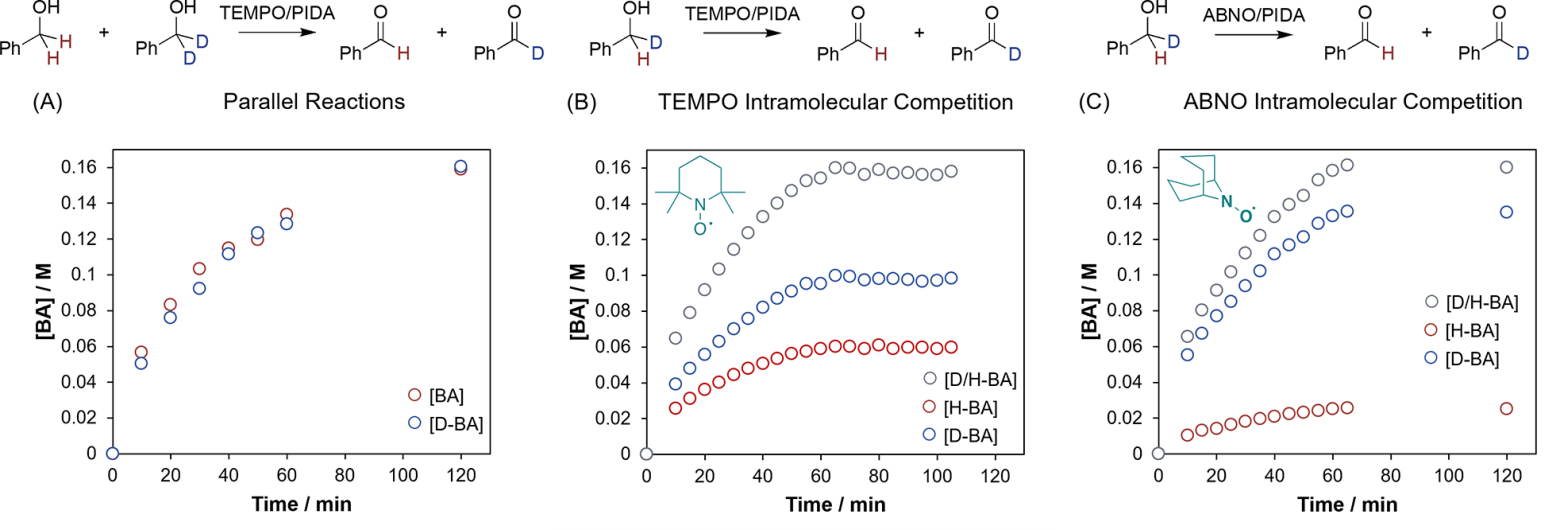
(A) Formation of benzaldehyde in parallel reactions for benzyl alcohol (red circles) and α,α-d_2_ benzyl alcohol (blue circles); the formation of benzaldehyde for an intramolecular competition reaction using α-d_1_ benzyl alcohol showing selectivity between H/D extraction using (B) TEMPO and (C) ABNO.

Oxidation of the alcohol likely occurs after the rate-determining step, which kinetic analysis shows must involve the alcohol, PIDA, and water. KIEs can be used to probe events after a rate-determining step, provided the isotopically labeled bond is involved in the product-determining step.^41^ To this end, we investigated the product ratio from an intramolecular competition KIE using monodeuterated α-d_1_ benzyl alcohol. The ratio of H/D incorporation in the product, rather than differences in rate, should be able to supply a KIE of the alcohol oxidation step, which is product-determining. Although no overall KIE is observed, selectivity toward the deuterated benzaldehyde derivative, and so preference for abstraction of the H atom during the oxidation step, is favored. With TEMPO, a KIE of 1.6 (Figure 6B) is observed, which is consistent with the reported KIE range of 1.8–3.6 for electrochemically generated oxoammonium species.^13,42^

Repetition of this experiment with ABNO, a sterically less encumbered TEMPO derivative, significantly changed the KIE to 5.4 (Figure 6C), similar to a literature value of the oxoammonium of ABNO of 3.7.^43^ The change in KIE indicates that the TEMPO derivative must be involved in the oxidation of the alcohol. The magnitudes of both KIEs are strongly supportive of an oxidation by an oxoammonium species rather than a radical process. Radical processes often display tunneling and result in much larger KIEs; the oxidation of benzyl alcohol through rate-determining hydrogen atom transfer by a NHPI/PINO system displays KIEs > 14.^44^

### Electrochemical Investigation

To further probe the relationship between TEMPO and PIDA, we performed cyclic voltammetry (CV) studies. For a solution of TEMPO, on increasing the oxidation potential (Figure 7A, blue line top, left to right), TEMPO is oxidized to the oxoammonium species, characterized by the oxidation peak at 0.26 V vs Fc^+^/Fc. Conversely, as the potential is then decreased (Figure 7A, blue line bottom, right to left), the oxoammonium is reduced back to TEMPO, with a reduction peak potential of 0.17 V vs Fc^+^/Fc. The flat regions at either side of the CV indicate that no additional redox processes occur within this potential range.

**Figure 7:**
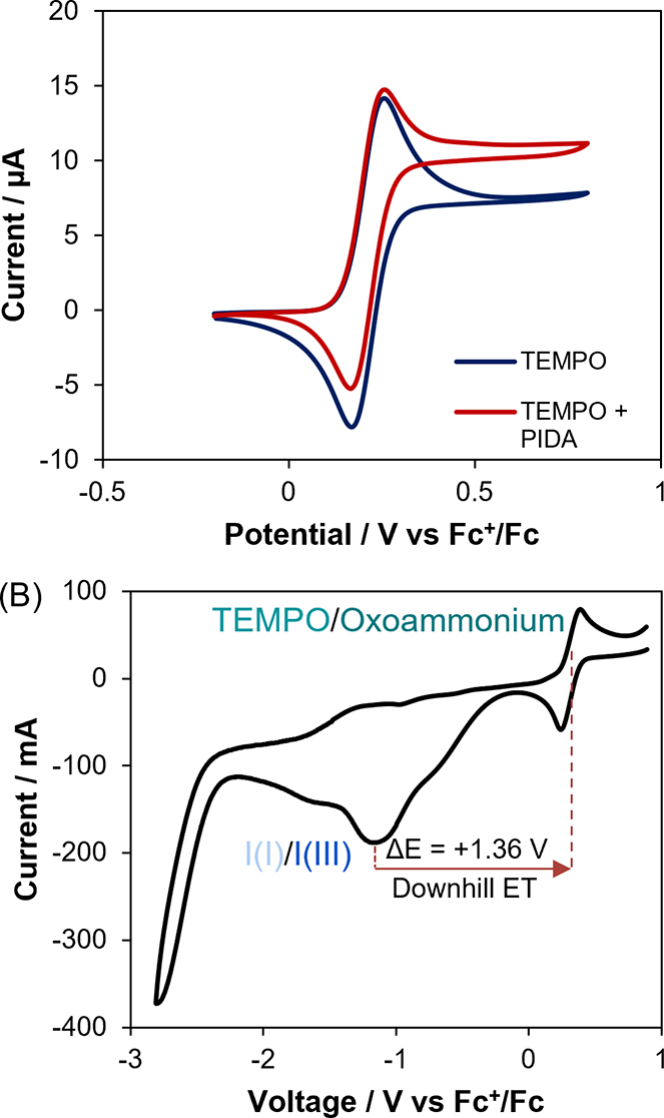
(A) CV curves of 3 mM TEMPO (blue line) and 3 mM TEMPO with 100 mM PIDA (red line); scan rate 10 mV s^–1^; (B) CV curves of 3 mM TEMPO and 100 mM PIDA; scan rate 100 mV s^–1^. Both CV curves are recorded in MeCN containing 0.8 M water and 0.1 M tetrabutylammonium perchlorate as supporting electrolyte.

Upon the addition of PIDA, the CV of TEMPO changed significantly (Figure 7A, red line). Electrochemical oxidation of TEMPO to oxoammonium still occurs at 0.26 V vs Fc^+^/Fc. However, a higher current is now observed, consistent with regeneration of TEMPO in the diffusion layer. Further, the electrochemical reduction peak at 0.17 V vs Fc^+^/Fc is diminished, as some of the oxoammonium species in the diffusion layer have been reduced back to TEMPO in a chemical step. The most likely explanation is that the electrochemically generated oxoammonium species undergoes a homogeneous chemical reaction with PIDA, resulting in the regeneration of TEMPO and the formation of an oxidized PIDA species. This process is consistent with the classical EC′ (electrochemical–chemical) catalytic mechanism.^45^

Examination of the redox characteristics of both TEMPO and PIDA over a wider potential range (Figure 7B) further supports the feasibility of electron transfer from PIDA to the oxoammonium species. The TEMPO/TEMPO^+^ couple is clearly visible with a midpoint potential at approximately +0.21 V, while PIDA displays an irreversible reduction peak at −1.25 V vs Fc^+^/Fc, corresponding to the iodine­(III)/iodine­(I) reduction.^46^ The large thermodynamic driving force (Δ*E* = +1.36 V) (Figure 7B) indicates that the electron transfer from PIDA to the oxoammonium species is energetically favorable, consistent with electrochemical thermodynamic principles.^45^

### Mechanistic Summary

It is possible to propose a mechanism that fits with the diverse results gathered so far as well as ruling several out. The fact that the reaction is positive order in three reagents (alcohol, PIDA, and water) suggests the potential for two rate-determining steps.^47–49^ The partial order in benzyl alcohol suggests two possible catalytic scenarios: either a split between resting states or the presence of a parasitic equilibria involving the alcohol, which reversibly removes the catalyst from the catalytic cycle. On monitoring the reaction by in situ ^1^H NMR, a monoalkoxy PIDA species can be detected (see Figure S6 in the Supporting Information), the concentration of which drops rapidly at the start of the reaction, suggesting a role as an off-cycle intermediate and fitting the latter scenario.

The TEMPO derivative clearly plays a complex role, one in which the oxoammonium form is central. While a change in the reaction rate on a change in the TEMPO derivative demonstrates that TEMPO must be involved ***prior*** to the rate-determining step (Figure 5), the KIE results (Figure 6) suggest that TEMPO must also be involved ***after*** the rate-determining step as well. This rules out any mechanistic scenario in which TEMPO is involved in alcohol oxidation only after some other rate-determining step(s) involving iodine, water, and alcohol (i.e., a simple extension of the existing hydroxylamine/oxoammonium cycle). The fact that the reaction displays zero-order behavior in TEMPO means the TEMPO derivative is not involved in the rate-determining step. Neither can TEMPO be present in any intermediates involved in the rate-determining step, as it is present in only catalytic quantities, and this would result in positive-order behavior. This rules out possible roles such as a Lewis acid or other activator.

However, TEMPO must be acting as a catalyst; it is required in only catalytic quantities, but as it is zero order, there must be a second catalytic cycle on which the rate-determining step(s) sit without involvement of TEMPO. Of the three other reagents displaying positive-order kinetics, PIDA seems the most likely to be acting in a catalytic context. If this were the case, then rate-determining ligand exchange with both water and alcohol would fit these positive-order kinetics. This also agrees with the inverse KIE observed with D_2_O (Figure 2) and the negative Hammett relationship (Figure 4). If hydrogen bonding plays an important role in ligand exchange at hypervalent iodine, as is suggested computationally,^50,51^ then deuterium would lower the barrier of this step as would a less acidic alcohol proton. PIDA must also be acting as the terminal oxidant, as it is reduced to iodobenzene and acetic acid during the reaction (see Figure S8 in Supporting Information). The requirement for two processes involving PIDA would fit with a dual catalytic cycle: one in which PIDA is reduced to I­(I), likely to oxidize the hydroxylamine to the invoked oxoammonium, and another in which oxidation of the alcohol occurs, likely overall redox-neutral for iodine.

Several hypothetical mechanistic scenarios can be considered to fit this line of reasoning (Figure 8). Scenarios in which iodine remains continually as I­(III) or is reoxidized from I­(I) to I­(III) can essentially be discarded. There is no room in either for TEMPO to be involved both before and after the rate-determining step, and neither can satisfactorily explain the role of water, without which the reaction does not proceed (see Figures S3 and S4 in the Supporting Information). Further complicating the matter, I­(III) compounds only have two labile ligands, yet three ligand exchanges (TEMPO, water, and alcohol) are required, each of which needs an acetate to proceed. Indeed, the lability of the ligands on I­(III) is an important point; if one of these is tethered to the phenyl ring, stabilizing the interaction, the reaction does not proceed (see Figure S9 in the Supporting Information).

**Figure 8:**
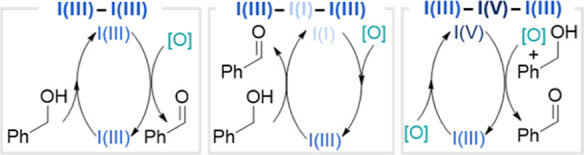
Hypothetical scenarios considering the order of addition of alcohol and oxidant, release of the carbonyl product, and oxidation states accessed by the hypervalent iodine reagent.

Electrochemical experimentation has shown that oxoammonium can oxidize PIDA (Figure 7). If this were from an I­(III) to an I­(V) species, this would account for the role of water as the additional ligand present to form the four, or possibly five, coordinate I­(V) species. The intermediacy of an I­(V) species would also account for the cleavage of 1,2-diols by TEMPO/PIDA, as both DMP^52^ and IBX^53^ are known to promote this transformation. This I­(V) could then perform a rate-determining ligand exchange with the alcohol before TEMPO re-enters the cycle to be involved in the oxidation step, thus leaving and returning outside rate-determining ligand exchanges (Figure 8).

### Computational Studies

Computational studies were conducted to model the feasibility of the proposed mechanism. Ligand exchange on PIDA can occur by two microscopic mechanisms: isomerization followed by association or concerted interchange.^50^ In all but one case, we find isomerization and then association to be lower in energy than the concerted pathway (see Table S3 in the Supporting Information). The first step of association is coordination of the substrate to the apical acetate via a hydrogen bond. This is an extremely shallow point on the potential energy surface, and we were able to find only a single instance of this transition state (**TS5**, vide infra). Ariafard and co-workers estimated the energy barrier to hydrogen bond formation to be roughly equal to that of isomerization, and so we use this energy barrier as a substitute where an explicit transition state could not be found.^50^

To begin the catalytic cycle (Figure 9; see Figure S11 in the Supporting Information for a simplified version showing only kinetically meaningful steps), on-cycle exchange with water occurs to form monohydroxy species **4** in the first rate-determining step with a barrier of at least 24.1 kcal mol^–1^. At this stage, PIDA can partake in an off-cycle equilibria with benzyl alcohol to form experimentally observed **4**_**OC**_ (see Figure S6 in Supporting Information). It is the formation of this relatively thermodynamically stable species that we consider the origin of the partial order in benzyl alcohol.

**Figure 9:**
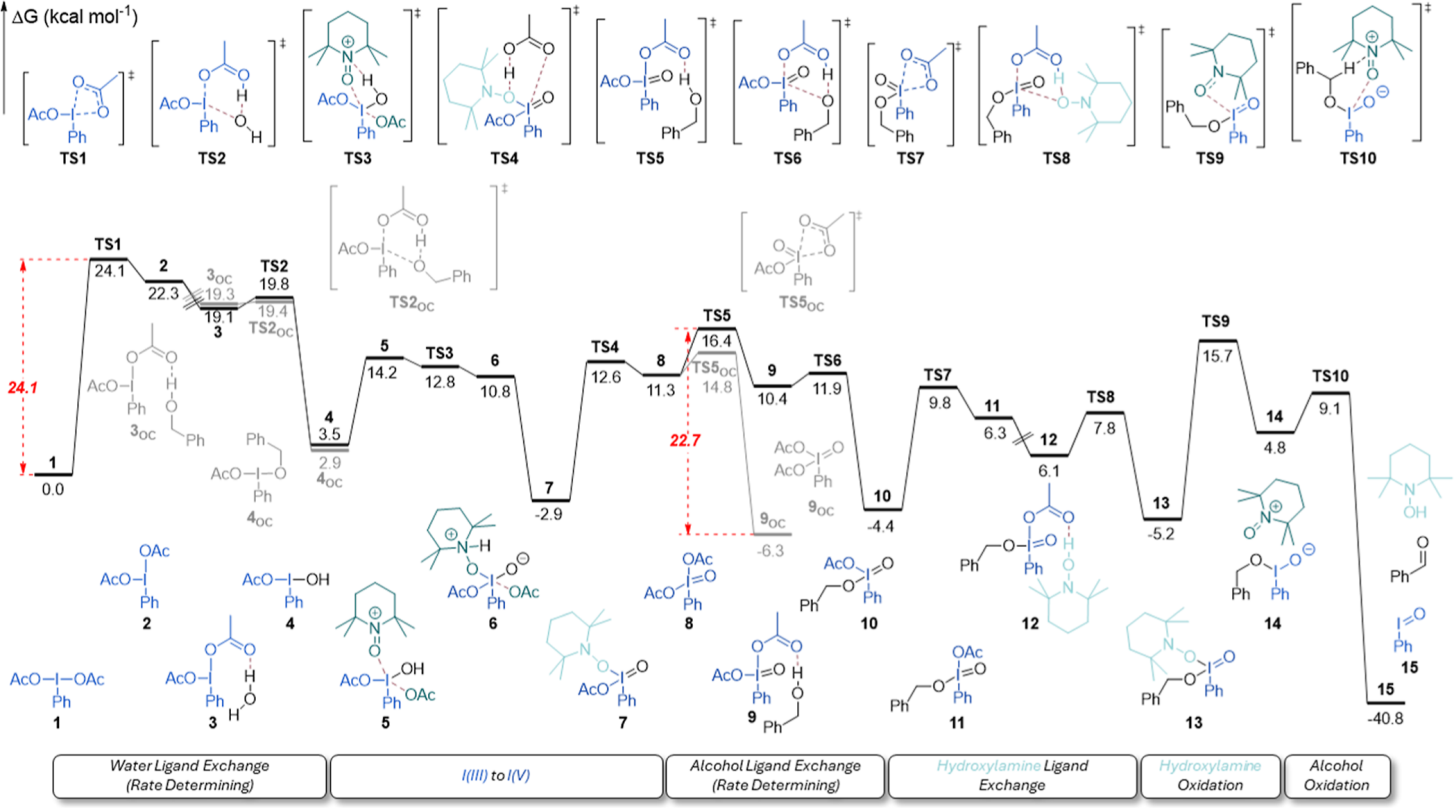
M06-2X/Def2TZVP/PCM_acetonitrile_//M06-2X/6-31G­(d,p)/SDD­(I)-computed Gibbs free energy (kcal mol^–1^) profile of the redox relay I­(III)–I­(V)–I­(III) mechanism. Important energy barriers of the two rate-determining steps are highlighted in red. A simplified energy surface, highlighting only kinetically meaningful or important structures, is available in Figure S11 in the Supporting Information.

Formation of monohydroxy hypervalent iodine **4** is an important first step, as this species can undergo oxidation by an oxoammonium to form the putative I­(V) species **7**. **TS3** is facile, with an activation barrier of 12.8 kcal mol^–1^. From **TS3** onward, the thermodynamic driving force of the reduction of PIDA on the second cycle of Figure 10, a large 23.4 kcal mol^–1^ benefit, is taken into account. In part, the reduction of I­(III) to I­(I) on one cycle acts as a strong driving force for the oxidation of a further I­(III) to I­(V) on another.

**Figure 10:**
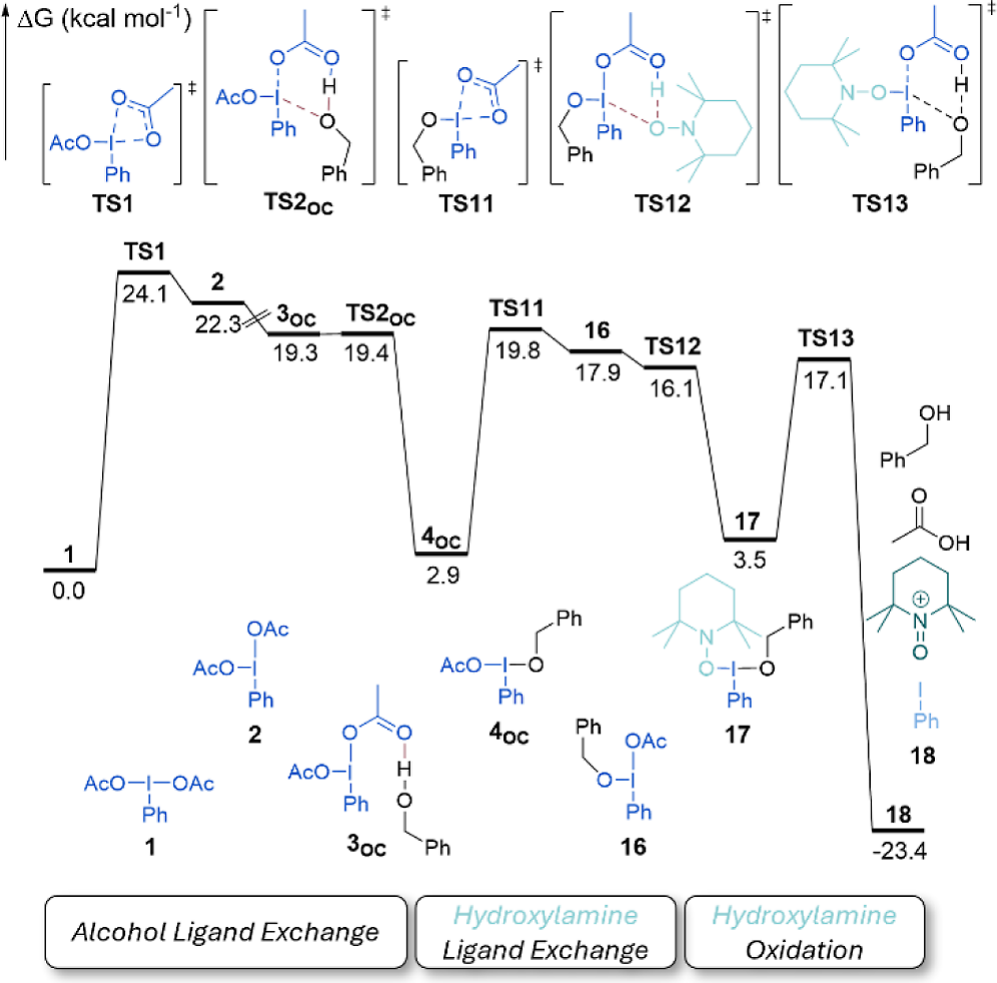
M06-2X/Def2TZVP/PCM_acetonitrile_//M06-2X/6-31G­(d,p)/SDD­(I)-computed Gibbs free energy (kcal mol^–1^) profile of oxidation of hydroxylamine to oxoammonium by PIDA.

Hydroxylamine is lost from **7** in **TS4** in a low-barrier ligand exchange with acetic acid, 12.6 kcal mol^–1^. Compound **8** can either isomerize to thermodynamically stable off-cycle **9**_**OC**_ or coordinate to benzyl alcohol to begin a ligand exchange process (see Table S3 in the Supporting Information). Compound **9**_**OC**_ represents a thermodynamic low on the potential energy surface. Maximizing the energy span suggests that **TS5**, the formation of a hydrogen bond between the apical acetoxy group of the hypervalent iodine and benzyl alcohol, is likely a second rate-determining step, with a barrier of 22.7 kcal mol^–1^.

The similarity in energy spans between this step and the initial ligand exchange of **TS1** computationally suggests two rate-determining steps and correctly predicts positive-order kinetics for iodine, water, and alcohol and zero-order kinetics for TEMPO.^54,55^ The fact that rate-determining **TS5** involves the formation of a hydrogen bond also matches experimental observations of the H_2_O/D_2_O inverse KIE and the Hammett study. The good agreement between the computational study and experimental kinetics acts as significant validation of the presented model.

Hydroxylamine now re-enters the catalytic cycle in an isomerization-association ligand exchange with the acetate of **10** to form **13**. The barrier to isomerization of the acetate on I­(V), **TS7**, is much lower in energy than that for I­(III). A hydrogen bond transition state moving from **11** to **12** could not be explicitly found.

Rather than intramolecular oxidation of the alcohol in **13** as might at first be expected of an I­(V) species, and as occurs with I­(V) oxidants Dess–Martin periodinane or 2-iodoxybenzoic acid (see Figures S13–S15 in the Supporting Information), the hydroxylamine is instead reoxidized to the oxoammonium with the now anionic hypervalent iodine I­(III) of **14** acting as a counterion. In this close ion pair, oxidation of the alcohol bound to the iodine, **TS10**, is facile, with a barrier of only 9.1 kcal mol^–1^. The hydroxylamine is reformed along with iodosobenzene **15**, which can undergo a series of known and kinetically unimportant steps in the presence of acetic acid to release water, reform PIDA, and close the catalytic cycle.

The oxoammonium required for turnover, experimentally central and participating in **TS3** of Figure 9, is formed in a second catalytic cycle, as shown in Figure 10. Direct ligand exchange between PIDA and the hydroxylamine has a slightly higher barrier than exchange with either water or benzyl alcohol at 20.3 kcal mol^–1^ (see Table S3 of Supporting Information). Although formation of the oxoammonium can occur from this species (see Figure S16 of the Supporting Information), a lower energy pathway is possible if beginning with the ligand between PIDA and benzyl alcohol, considered off-cycle in Figure 9. Ligand exchange with hydroxylamine from monoalcohol hypervalent iodine species **4**_**OC**_ in **TS11** is significantly lower in energy. Rather than initiating a further ligand exchange, the approach of an acetate in **TS13** leads directly to reduction of PIDA and oxidation of the hydroxylamine to the desired oxoammonium with the acetate group acting directly as a counterion.

### Rate Equation

Good agreement between computational modeling and experimental results provides confidence in the updated mechanistic model (Scheme 5). A rate equation of this dual-catalytic system involving redox-relay between hypervalent iodine and TEMPO oxidation states can be derived (eq 1, see Section S9 in the Supporting Information).

1 $$rate=k_{4}{{K_{1}K}_{2}K}_{3}\frac{\left[BnOH\right]\left[H_{2}O\right]{\left[PIDA\right]}_{T}}{1+K_{OC}\frac{\left[BnOH\right]}{\left[AcOH\right]}}$$

**Scheme 5:**
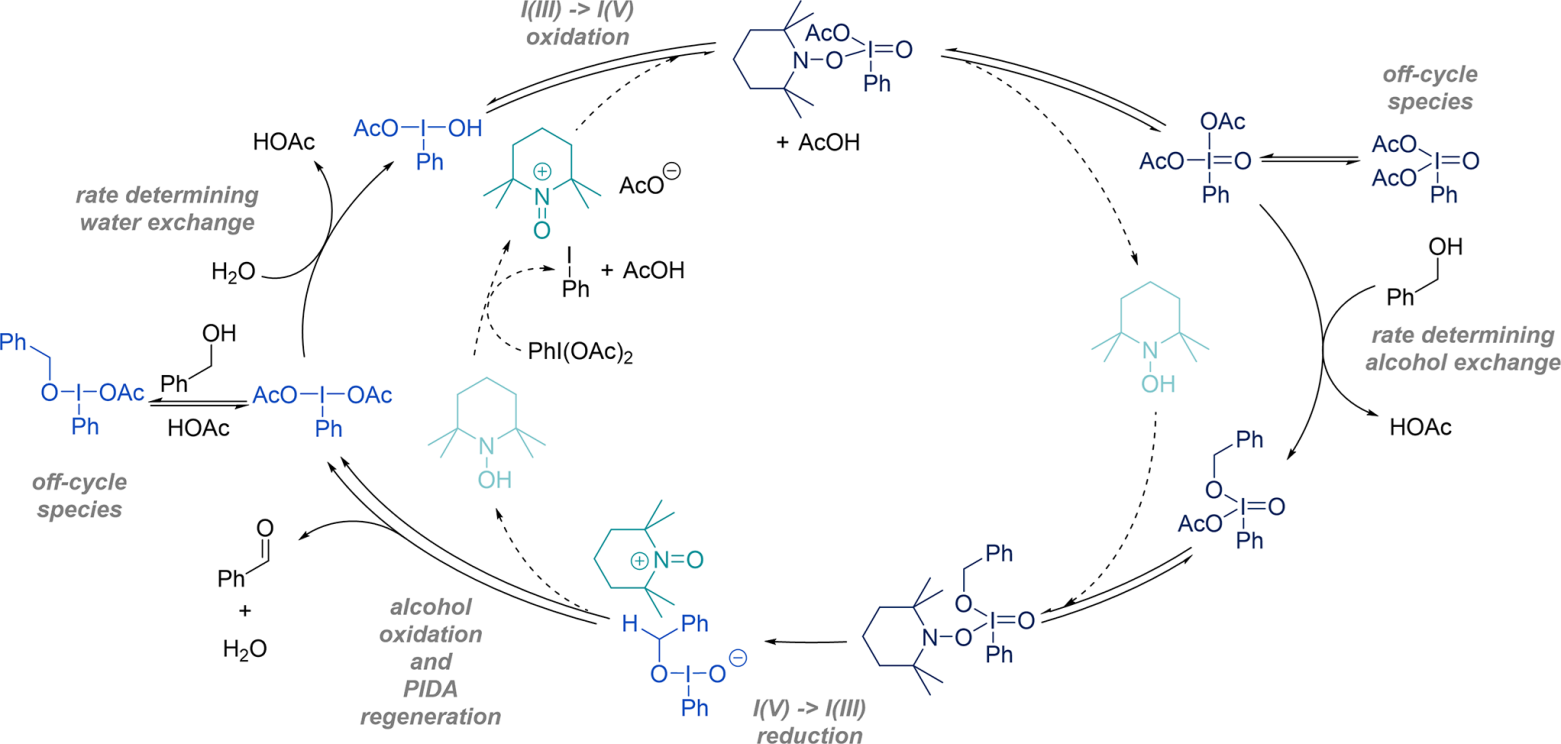
Proposed Dual-Catalytic Redox Relay Reaction Mechanism, Highlighting Both Catalytic Cycles, Two Rate-Determining Steps, and Off-Cycle Species

This is consistent with the observed kinetics, where the rate is dependent on PIDA and water as well as having a fractional dependence upon benzyl alcohol and no dependence on TEMPO. In line with in situ NMR experiments (see Figure S6 in the Supporting Information), a shift in the turnover-determining intermediate, in which monoalkoxy species **4**_**OC**_ becomes negligible toward the start of the reaction, results in zero-order dependence on AcOH and simplification of the rate equation by removal of the *K*_OC_[BnOH]/[AcOH] term. Rate-determining ligand exchange on iodine with water and benzyl alcohol accounts for the positive-order kinetics for these substrates as well as the Hammett relationship and D_2_O inverse KIE, pointing to an energetically challenging formation of a hydrogen bond to initiate the ligand exchange process. Off-cycle formation of a monoalcohol hypervalent iodine adduct explains the partial positive order in benzyl alcohol. Electrochemical results incorporate the reduction of the oxoammonium by iodine and the subsequent formation of an I­(V) compound. KIEs and TEMPO derivative control experiments account for the presence of TEMPO both before and after the rate-determining steps and the involvement of the oxoammonium in the oxidation of the alcohol.

### Oxidative Kinetic Resolution of Secondary Alcohols

Our mechanism suggests that iodine, while not directly oxidizing the alcohol, is present during the oxidation step. To further test this hypothesis, we reasoned that the oxidative kinetic resolution (OKR) of secondary alcohols using a chiral hypervalent iodine­(III) derivative should be possible.^56^

OKRs of secondary alcohols mediated by chiral hypervalent iodine­(V) reagents are known.^57,58^ OKR by iodine­(III) reagents has been reported, with highly specific examples of the resolution of a secondary homoallylic alcohol via oxidative lactonization^59^ and the dearomatization of naphtholic alcohols.^60,61^ While PIDA has been used in direct OKR when employing a chiral TEMPO derivative,^62^ there are no examples of iodine­(III)-mediated OKR of secondary alcohols without either neighboring group participation or a secondary step.

We synthesized a range of chiral hypervalent iodine reagents^63^ and tested them against a model alcohol (see Section S10 of the Supporting Information). Proof of principle of direct I­(III) OKR was demonstrated with chiral hypervalent iodine **34-OAc** returning the highest *ee* of 17% and an *S*-factor of 1.8 (Scheme 6). Although not currently representing levels of selectivity required for synthetic utility, this result nevertheless provides additional support toward our proposed mechanism. While KIE studies have shown that the TEMPO derivative must oxidize the alcohol, OKR demonstrates that hypervalent iodine, as the source of chirality, must also be present during oxidation.

**Scheme 6:**
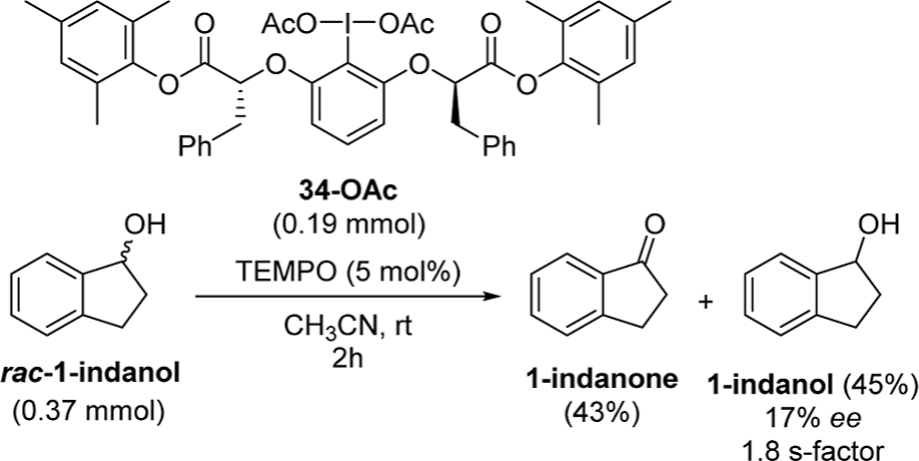
Direct OKR of a Secondary Alcohol Using a Chiral Hypervalent Iodine­(III) Reagent

## Conclusion

In summary, this study has provided extensive insights into the mechanism of the hypervalent iodine and TEMPO-catalyzed oxidation of alcohols, leading to the rational development of new reactivity in the OKR of secondary alcohols by a chiral hypervalent iodine­(III) derivative. The updated mechanism is demonstrated to be significantly more complex than previously assumed, differing from the standard oxoammonium pathway as well as the integrated or serial cooperativity regimes proposed for other TEMPO-based cocatalytic oxidation systems. Instead, we propose a dual-catalytic redox relay mechanism in which PIDA, as the terminal oxidant on one catalytic cycle, oxidizes TEMPO to an oxoammonium. On a second catalytic cycle, the oxoammonium in turn oxidizes catalytic quantities of a monohydroxy-I­(III) species to an I­(V) species. This I­(V) species binds the alcohol and then is reduced back to I­(III) as it reoxidizes TEMPO to an oxoammonium. This oxoammonium, in a tight ion pair, oxidizes the I­(III)-bound alcohol, returning PIDA, water, and aldehyde to close the catalytic cycle.

The study demonstrates the mechanism to be general across most alcohols. A synthetically useful finding is that the simple addition of water can drastically speed up the rate of this popular reaction, as can the choice of alternative TEMPO derivatives and the requirement for only low loadings of TEMPO. The transient access of an I­(V) species, monitored electrochemically and supported computationally, implicates the development of new hypervalent iodine chemistry and is worthy of future investigation.

## Supplementary Material



## Data Availability

All data created during this research are openly available from the PURE data repository at https://doi.org/10.17034/c0faf1af-03db-488b-9f53-94b3c675cc15.

## Notes

Materials and methods, NMR spectra, kinetic data and plots, derivations, Cartesian coordinates, electronic energies, and imaginary frequencies (PDF)

## §

Department of Life Sciences, Atlantic Technological University, Ash Lane, F91 YW50, Sligo, Ireland

## ∥

Department of Chemistry Education, Seoul National University, Seoul 08826, Republic of Korea.

## ⊥

College of Science, Princess Nourah bint Abdulrahman University, PO Box 84428, Riyadh 11671, Saudi Arabia.
