# Supplementary material for: Revealing the Mechanism of TEMPO-Hypervalent Iodine(III) Oxidation of Alcohols
Source: J Am Chem Soc. 2026 Feb 19;148(8):8852–62. doi: 10.1021/jacs.5c21609 (PMC12964531; doi:10.1021/jacs.5c21609)
Supplement: Supplementary file 1 [file ja5c21609_si_001.pdf]

Supporting Information for

Revealing the Mechanism of TEMPO-Hypervalent Iodine(III) Oxidation of Alcohols

Michael Bingham,[a] Tapas R. Pradhan,[b] Dhananjay Bhattacherjee,[b] Rawiyah Alkahtani,[b] Paul Kavanagh*,[c] Thomas Wirth*,[b] Paul Dingwall*[c]

AUTHOR ADDRESS

[a] *Department of Life Sciences, Atlantic Technological University, Ash Lane, F91 YW50, Sligo, Ireland*

[b] *School of Chemistry, Cardiff University, Main Building, CF10 3AT, Cardiff, Cymru/Wales*

[c] *School of Chemistry and Chemical Engineering, Queen's University Belfast, BT7 1NN, Belfast, Northern Ireland*

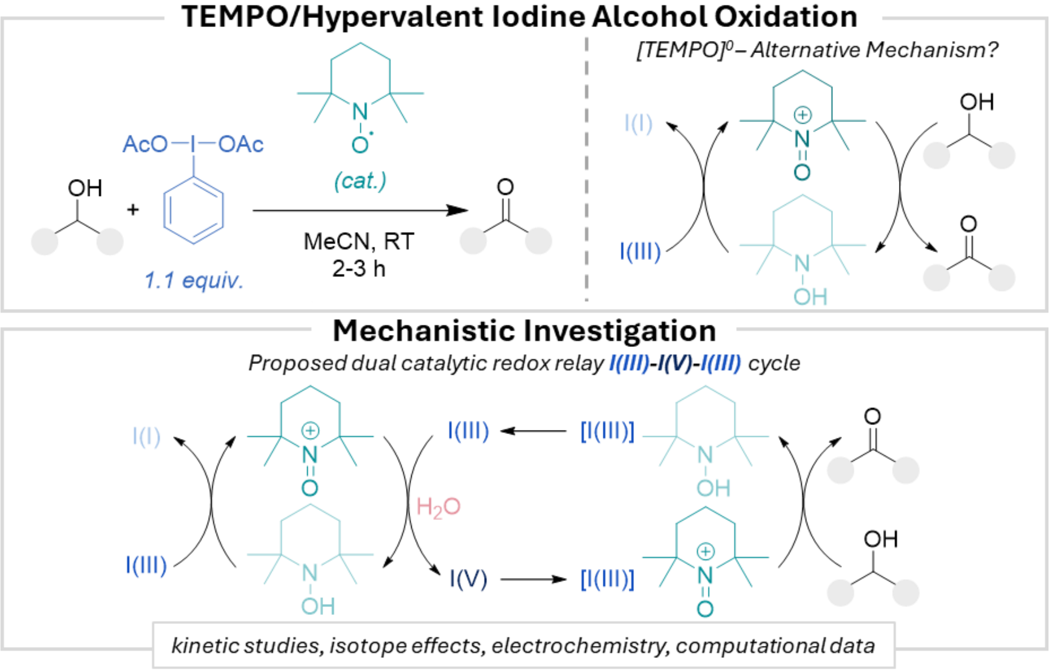

Table of Contents

1. Experimental 4
   1.1 Materials and instrumentation. 4
   1.2 Synthesis of α-d_1-benzyl alcohol.[1-2] 6
   1.3 Synthesis of α,α-d_2-benzyl alcohol.[3] 6
   1.4 Synthesis of 3,3-dimethyl-1λ^3-benzo[d][1,2]iodaoxol-1(3H)-yl acetate 7
   1.5 General Procedures for the Synthesis of C2-Symmetric Chiral Hypervalent Iodine 9
   1.6 Reagents 10
   1.7 NMR data 19
2. Kinetic Analysis 39
   2.1 Standard kinetic procedure: Reaction sampling 39
   2.2 Kinetic procedure: In situ NMR 39
   2.3 Standard reaction conditions for kinetic investigation 39
   2.4 Kinetic Plots 40
   2.5 Varying [TEMPO] 41
3. Standard reaction under Schlenk conditions 42
4. Reactions with increasing [H_2O] 43
5. Different alcohol VTNA 45
6. Mono-alkoxy PIDA species 46
7. Stoichiometric acetic acid formation 47
8. Ligand tethered hypervalent iodine species 48
9. Derivation of rate equation 49
10. Oxidative Kinetic Resolution Optimisation 52
11. Density Functional Theory (DFT) calculations 55
   11.1 Computational Details 55
   11.2 Comparison of PIDA Ligand Exchange Pathways 56
   11.3 Simplified Potential Energy Surface 58
   11.3 TS5 Hydrogen Bonds 60
   11.4 Comparison of Alcohol Oxidation Transition States 61
   11.5 Oxidation of Hydroxylamine by PIDA via Direct Ligand Exchange Between PIDA and Hydroxylamine 63
   11.6 Catalytic Pathway for Different Alcohols 64
12. DFT Cartesian Coordinates 65

12.1 Starting Materials and Products 65
12.2 Cycle 1: Alcohol Oxidation 67
12.3 Cycle 2: TEMPO Oxidation 82
12.4 Cycle 2_SI: TEMPO Oxidation 85
13. References 89

1. *Experimental*

1.1 Materials and instrumentation.

Reagents were purchased from Apollo Scientific, Acros Organics, Alfa Aesar, Fisher Scientific, FluoroChem, Merck, Sigma Aldrich and TCI were used as received without purification.

Dry solvent of diethyl ether, tetrahydrofuran, toluene, and acetonitrile were collected from a solvent purification system (SPS) stored under a nitrogen atmosphere, which is from the company M BRAUN (MB SPS-800). Dry dichloromethane was freshly distilled from calcium hydride under air or dried nitrogen atmosphere.

Thin layer chromatography (TLC) was performed to monitor the reactions using precoated aluminium sheets of Merck silica gel 60 F254 (0.20 m), and detection of compounds was performed under UV light (254 nm) or dipping into a solution of KMnO4 (1.5 g in 200 mL H2O, 5 g NaHCO3).

Flash chromatography (Combi Flash NEXTGEN 300+ TELEDYNE ISCO) was carried out using silica gel (Redi Sep Rf Gold, 24 Gram Flash Column), as mentioned. Eluting solvents for chromatographic purification (n-hexane, MeOH and EtOAc) were purchased from commercial sources and used directly for the purification indicated in the text.

Optical rotations were measured with a SCHMIDT and HAENSCH UniPol L polarimeter at 20 °C in a cuvette of 50 mm length with a sodium light (589.30 nm). HPLC grade chloroform was used to prepare the solution, and the concentration is indicated in the data section.

High-performance liquid chromatography (HPLC) analysis was conducted using Shimadzu Prominence HPLC System equipped with LC-10 AD coupled diode array-detector SPD-MA-10AVP and chiral column. The solvents used were HPLC grade of *n*-hexane and 2-propanol. The chiral columns that used for the separation of enantiomers were YMC Chiral Amylose C S-5 μ m (0.46 cm O x 25 cm), Daicel Chiralcel R OD-H (0.46 cm O x 25 cm), and CHIRALPAK R IC (0.46 cm O x 25 cm). Data are reported as follows: column type, eluent, wavelength, flow rate, retention time (tR).

For kinetic studies (Queen's University Belfast), ^{1}H NMR was conducting using a 400 MHz Bruker NMR with 32 scans. High resolution NMR was carried out on a 600 MHz Bruker NMR with 32 scans for in-situ experiments. Chemical shifts (δ) are quoted in parts per million (ppm) and were measured against trimethylsilane (TMS), residual chloroform ($CHCl_3$), or 1,2-dichloroethane as an internal standard. ^{1}H NMR spectra are reported as follows: chemical shift (number of protons, multiplicity, coupling constant). Multiplicity is abbreviated as follows: s = singlet, br = broad, d = doublet, dd = doublet of doublets, ddd = double, doublet of doublets, t = triplet, q = quartet, m = multiplet.

For synthetic studies (Cardiff), ^{1}H NMR spectra were obtained in $CDCl_3$ at 300 MHz, 400 MHz, or 500 MHz. Chemical shifts are reported in ppm and referenced to the $CDCl_3$ singlet at 7.26 ppm. $^{13}C\{^{1}H\}$ NMR spectra were obtained in $CDCl_3$ at 75 MHz, 100 MHz, or 126 MHz and referenced to the center of the $CDCl_3$ triplet at 77.16 ppm. The abbreviations s, d, t, q, quint, sext, sept, dd, ddd, dt and m stand for the resonance multiplicities singlet, doublet, triplet, quartet, quintet, septet, doublet of doublets, doublet of doublet of doublets, doublet of triplets and multiplet, respectively.

High resolution mass spectrometry (HRMS) analyses at Queen's University Belfast were conducted by the Analytical Services and Environmental Projects (ASEP) service on a Waters LCT Premier ToF mass spectrometer in ESI positive-ion mode. HRMS at Cardiff University were performed by the Analytical services CHEMY Mass Spectrometry Facility on either a Waters Xevo G2S, a Waters GCTOF spectrometer, or a Thermo Scientific Exactive GC orbitrap (short temperature gradient). Ions were generated using electron ionisation (EI), chemical ionisation (CI) atmospheric pressure chemical ionisation (APCI), or atmospheric-solid-analysis-probe (ASAP) techniques. All signals are reported with a mass-to-charge (m/z) ratio unit. Software: MassLynx Mass Spectrometry Software (Waters).

Cyclic voltammetry (CV) experiments were carried out using a PalmSens4 potentiostat. Measurements were conducted in a single-compartment electrochemical cell equipped with a glassy carbon disc working electrode (3 mm diameter), a platinum wire counter electrode, and a silver/silver ion (Ag/Ag^{+}) pseudo-reference electrode. The electrolyte solution consisted of acetonitrile containing 0.8 M water and 0.1 M tetrabutylammonium perchlorate (TBAP) as the supporting electrolyte. All potentials were referenced internally to the ferrocenium/ferrocene (Fc^{+}/Fc) redox couple.

1.2 Synthesis of α-d_1-benzyl alcohol.[1-2]

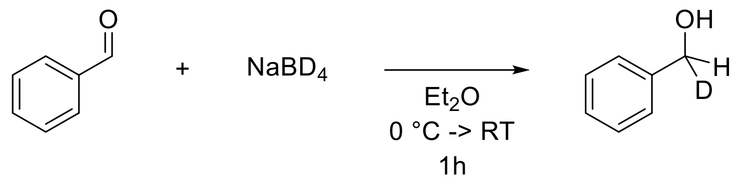

Sodium borodeuteride (0.10 g, 2.5 mmol, 1.0 equiv.) was added to a stirred solution of benzaldehyde (0.25 mL, 2.5 mmol, 1.0 equiv.) in diethyl ether (10 mL) and methanol (2 mL) at 0 °C. The mixture was allowed to react at ambient temperature and stirred for 1 h. The reaction was quenched by the addition of saturated aqueous NH_4Cl (10 mL). Water (10 mL) was added, and the aqueous phase was extracted with ethyl acetate (3 x). The combined organic layers were washed with brine, dried ($MgSO_4$) and concentrated in vacuo to give α-deuterobenzyl alcohol (0.312 g, >98%, 99 % D incorporation by ^{1}H NMR) as a colourless oil that was used without further purification.

^{1}H NMR (400 MHz, $CDCl_3$) δ = 7.35–7.29 (5 H, m), 4.69–4.66 (1 H, m), 5.1 (1 H, br s).

1.3 Synthesis of α,α-d_2-benzyl alcohol.[3]

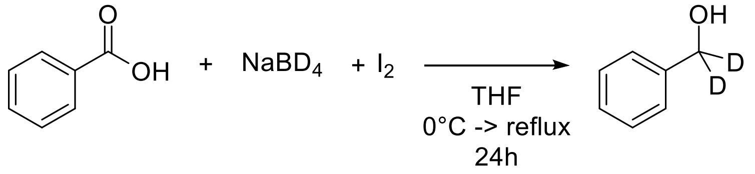

Sodium borodeuteride (1 g, 24 mmol) was dissolved in THF (40 mL, dried over mol. Sieves). Benzoic acid was then added (1.95 g, 76 mmol). The flask was then cooled to 0 °C using an ice bath. An I_2 solution (4.06 g, 16 mmol) in THF (50 mL) was added dropwise to the flask. After gas evolution had stopped, the flask was heated to reflux for 24 h and then cooled to room temperature. Methanol was then added dropwise until the solution turned clear and left to stir for 30 mins. The solvent was removed by rotary evaporation leaving a white paste. This was then dissolved in 20% aqueous KOH (35 mL) and stirred for 4 hours followed by an extraction with CH_2Cl_2 (30 mL x 2). The combined organic layers were washed with brine, dried over magnesium sulphate and concentrated under vacuum to give α,α-d_2-benzyl alcohol (1.84 g, >98%, 99 % D incorporation by ^{1}H NMR) which was used without further purification.

^{1}H NMR (400 MHz, $CDCl_3$) δ 7.33–7.28 (4 H, m), 5.1 (1 H, br s).

*1.4 Synthesis of 3,3-dimethyl-1λ³-benzo[d][1,2]iodaoxol-1(3*H*)-yl acetate*

2-(2-iodophenyl)propan-2-ol (23)

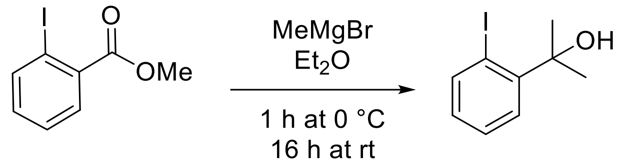

22 **23**

Following reported procedure,[4] in 50 mL dried round bottom flask solution of methyl Grignard (methylmagnesium bromide) reagent in Et_2O (6.4 mL, 3 M, 19.08 mmol, 2.5 equiv.) was added dropwise to a stirred solution of methyl-*o*-iodobenzoate **1** (2 g, 7.63 mmol, 1 equiv.) in dry Et_2O (30 mL) at 0 °C under nitrogen atmosphere. The mixture was warmed to room temperature over 1 h, monitoring through TLC (10% EtOAc in petroleum ether). The reaction was quenched after 16 h through the addition of saturated aqueous NH_4Cl (50 mL). The aqueous phase was extracted with Et_2O (3 x 30 mL). The combined organic phases were dried over $MgSO_4$ and concentrated in vacuum. The crude mixture was purified two times. First purification by flash column chromatography (petroleum ether/ethyl acetate: 9/1). The second time with flash column chromatography with slow flowrate using only chloroform solvent to yield the title product in 35% as colorless oil.

^{1}H NMR (300 MHz, $CDCl_3$) δ 7.96 (dd, *J* = 7.8, 1.3 Hz, 1H), 7.63 (dd, *J* = 8.0, 1.7 Hz, 1H), 7.36 – 7.29 (m, 1H), 6.90 (ddd, *J* = 7.8, 7.3, 1.7 Hz, 1H), 2.55 (s, 1H), 1.76 (s, 6H).

^{13}C{^{1}H}NMR (75 MHz, $CDCl_3$) δ 148.5, 142.8, 128.7, 128.2, 126.8, 93.3, 73.7, 29.9.

Data are in agreement with the literature.[4-5]

*3,3-dimethyl-λl^3-benzo[d][1,2]*iodaoxol-*1(3*H*)-yl acetate (24)*

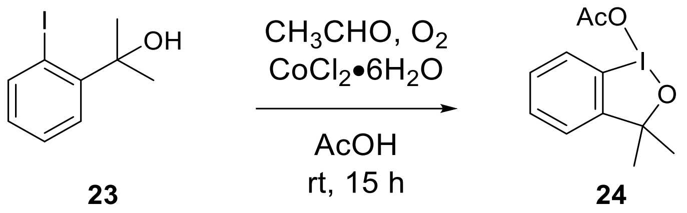

Following a reported procedure,[6] a dry round bottom flask was charged with glacial AcOH (16 mL), 2-(2-iodophenyl)propan-2-ol (0.60 g, 2.29 mmol, 1.0 equiv.) and $CoCl_2$•$6H_2O$ (3 mg, 0.023 mmol, 1 mol%) and was fitted with a rubber septum. The reaction vessel was purged with O_2 for 5 min before acetaldehyde (1.04 g, 1.3 mL, 23.58 mmol, 10.3 equiv.) was added in one portion. The reaction mixture was stirred under O_2, delivered by two inflated balloons, at 23 °C for 15 h. The solvent was removed in vacuo and residue was dissolved in CH_2Cl_2. The organic layer was washed with distilled water (15 mL) and extracted with CH_2Cl_2 (3 x 15 mL). The organic layer was dried over $MgSO_4$ and solvent was removed in vacuo. The obtained residue was washed with petroleum ether to afford the title compound as beige solid (99% yield).

^{1}H NMR (300 MHz, $CDCl_3$) δ 7.78 (d, *J* = 7.5 Hz, 1H), 7.56 – 7.35 (m, 2H), 7.17 (d, *J* = 6.9 Hz, 1H), 2.10 (s, 3H), 1.51 (s, 6H).

^{13}C{^{1}H}NMR (75 MHz, $CDCl_3$) δ 177.4, 149.4, 130.4, 130.0, 129.9, 126.2, 115.7, 84.6, 29.2, 21.5.

HRMS (CI) *m/z* $[M+H]^+$ calcd for $C_{11}H_{14}O_3I$ 320.99821, found 320.9984.

Data are in agreement with the literature.[4, 6]

1.5 General Procedures for the Synthesis of C2-Symmetric Chiral Hypervalent Iodine

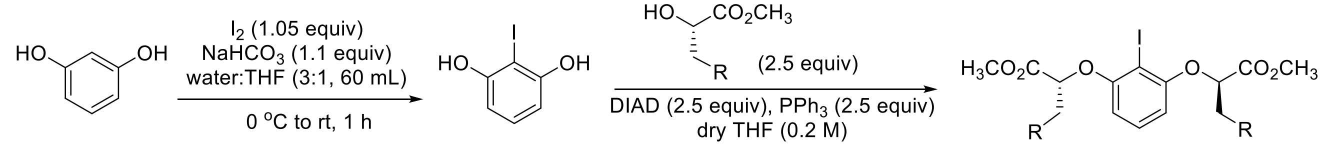

(5.0 g, 45.4 mmol)

25

53%

26

R = H, Ph

27, R = H, 84%
28, R = Ph, 78%

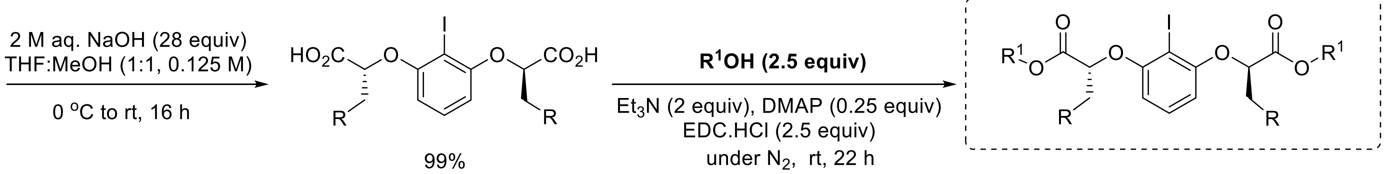

99%

R^1 = aryl / alkyl / benzyl

29, R = H, 99%
30, R = Ph, 99%

31, R = H, R^1 = mesityl, 55%
32, R = H, R^1 = menthol, 51%
33, R = H, R^1 = 1-phenyl ethyl, 55%
34, R = phenyl, R^1 = mesityl, 51%

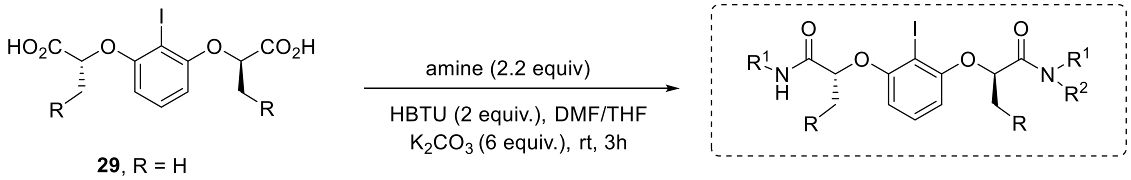

29, R = H
30, R = Ph

35, R = H, R^1 = phenyl, R^2 = phenyl, 78%
36, R = H,R^1 = H, R^2 = 1-phenyl ethyl, 77%

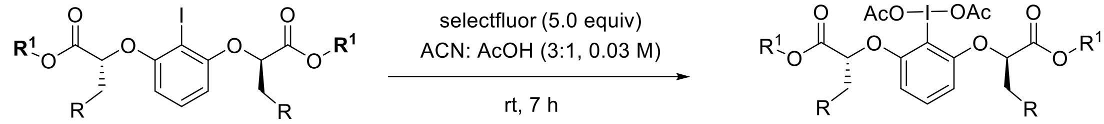

31-OAc, R = H, R^1 = mesityl, 94%
32-OAc, R = H, R^1 = menthol, 91%
33-OAc, R = H, R^1 = 1-phenyl ethyl, 93%
34-OAc, R = phenyl, R^1 = mesityl, 91%

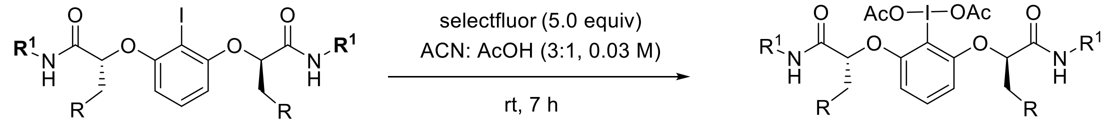

35-OAc, R = H, R^1 = phenyl, R^2 = phenyl, 88%
36-OAc, R = H,R^1 = H, R^2 = 1-phenyl ethyl, 90%

1.6 Reagents

All Reactions involving air and moisture sensitive reagents were carried out in dried glassware under a dry nitrogen atmosphere using Schlenk technique or using a balloon. All reactions were stirred using magnetic stirring, and for the need of heating, over a hotplate with a temperature probe control and an adapted heating block. Lower temperatures reactions were achieved by using ice/water bath (0 °C), dry ice/acetone bath (⁻78 °C) or using a chiller (0 to ⁻20 °C). Buchi rotavapors were used for solvent evaporations, and a high vacuum apparatus was used to further dry the products.

General Procedure 1 (GP1): The Mitsunobu reaction of the 2-iodoresorcinol derivatives

2-iodoresorcinol (4.79 g, 20.3 mmol), triphenylphosphine (13.32 g, 50.8 mmol) and corresponding methyl (*S*)-(-)-lactate / (S)-Methyl 2-hydroxy-3-phenylpropanoate derivative (50.8 mmol) were dissolved in dry THF (100 mL) under N_2 and wrapped in aluminium foil. The reaction was cooled at 0 °C and DIAD (10 mL, 50.8 mmol) was added dropwise. After 1 h at 0 °C, the mixture was warmed up to room temperature and stirred overnight. The solvent was removed under vacuum and Et_2O (100 mL) was added. The triphenylphosphine oxide, which precipitated, was removed by filtration and the filtrate was evaporated under vacuum. The product was obtained after column chromatography (0 to 20% EtOAc in hexane) as a colourless solid.

General Procedure 2 (GP2): The basic hydrolysis of diesters

The iodoarene (3.00 mmol) was dissolved in THF (12 mL) and MeOH (12 mL). After cooling the solution to 0 °C, a 2 M aqueous solution of NaOH (12 mL, 24 mmol, 8.0 equiv.) was added slowly and the resulting solution was stirred at room temperature for 16 h. The reaction mixture was then acidified with 3 M aqueous HCl at 0 °C and extracted with EtOAc (3 x 15 mL). The combined organic layers were washed with brine (30 mL), dried over anhydrous MgSO4 and concentrated under vacuum to afford the pure product.

General Procedure GP3 for the formation of esters

Dicarboxylic acid (1.052 mmol), alcohol / phenol (2.63 mmol, 2.5 equiv.), dry triethylamine (2.1 mmol, 2.0 equiv.) and 4-DMAP (0.26 mmol, 0.25 equiv.) were dissolved in dry CH_2Cl_2 (15 mL) in a flame-dried Schlenk tube under nitrogen atmosphere and EDC×HCl (2.63 mmol, 2.5 equiv.) was added. After the solution was stirred at room temperature for 22 h, 3 M aqueous HCl was added and the resulting mixture was extracted with CH2Cl2 (3 x 10 mL), dried over anhydrous $MgSO_4$ and concentrated under vacuum. The crude mixture was purified by flash column chromatography (n-hexane:EtOAc = 90:10) to afford desired esters as a colourless solid in 51-55% yield.

General Procedure GP4 for the formation of amides

A portion of the acid derivative of iodoarenes (1.052 mmol) was dissolved in DMF (5 mL) and THF (2 mL). To this solution was added aromatic / benzylamine (2.1 mmol), potassium carbonate (871 mg, 6.3 mmol), and HBTU (796 mg, 2.1 mmol). The reaction was allowed to stir for 3 h, after which time water (50 mL) was added. The crude mixture was extracted with

ethyl acetate (2 x 50 mL). The organic layer was washed with 1% aqueous sodium carbonate (5 x 50 mL) and brine (1 x 25 mL), dried over anhydrous Na_2SO_4, filtered, and concentrated under reduced pressure to afford the unpurified amide in 78-82% yield.

General Procedure GP5 for the oxidation of iodoarenes with Selectfluor®

The iodoarene (1.00 mmol) was dissolved in CH_3CN (12 mL) and glacial acetic acid (4 mL) under nitrogen atmosphere. Selectfluor^R (708 mg, 2.00 mmol) was added subsequently, and the resulting suspension was stirred at room temperature for 7 h. After completion of the reaction, the solvents were removed under vacuum, and the product was dissolved in $CHCl_3$. After filtration under nitrogen atmosphere, the filtrate was concentrated under reduced pressure, and the residue was washed with Et_2O/*n*-hexane (3:1) to afford the pure product.

2-Iodobenzene-1,3-diol (26)

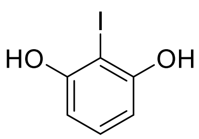

Resorcinol (5.0 g, 45.4 mmol) and iodine (12.3 g, 48.4 mmol) were dissolved in H_2O (32 mL) and cooled down to 0 °C. Sodium bicarbonate (4.23 g, 50.3 mmol) was added slowly with vigorous stirring. After 30 minutes, the brown slurry mixture was extracted with EtOAc (3 x 40 mL). The combined organic layers were washed with sat. aq. sodium thiosulfate solution (100 mL) and brine (100 mL), dried over MgSO4 and concentrated under vacuum. The resulting solid was triturated with cold $CHCl_3$ (20 mL, -10 °C) and left to crystallize at -10 °C. The pale brown crystals were filtered, washed with cold $CHCl_3$ and dried under vacuum (53% yield, 5.63 g, 23.8 mmol).

M.p. = 106 - 107 °C (lit.[3] 105 –108 °C).

^{1}H NMR (300 MHz, MeOD) δ 6.88 – 6.81 (m, 1H), 6.23 (d, *J* = 8.1 Hz, 2H), 4.81 (brs, 2H) ppm.

^{13}C NMR (75 MHz, MeOD) δ 157.92, 128.99, 105.50, 73.97 ppm.

The spectroscopic data are in agreement with literature.[7]

Dimethyl 2,2'-((2-iodo-1,3-phenylene)bis(oxy))(2R,2'R)-dipropionate (27)

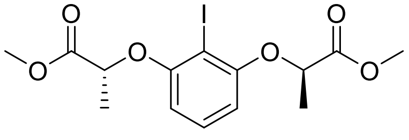

Prepared as per experimental procedure **GP1** using methyl (*S*)-(-)-lactate (4.85 mL, 50.8 mmol) to give **27** as a colourless solid in 84% yield (6.96 g, 17.1 mmol) after flash column chromatography (*n*-hexane:EtOAc = 80:20).

^{1}H NMR (300 MHz, $CDCl_3$) δ 7.13 (t, *J* = 8.3 Hz, 1H), 6.36 (d, *J* = 8.3 Hz, 2H), 4.77 (q, *J* = 6.8 Hz, 2H), 3.74 (s, 6H), 1.69 (d, *J* = 6.8 Hz, 6H) ppm.

^{13}C NMR (75 MHz, $CDCl_3$) δ 172.02, 158.07, 129.52, 106.71, 80.39, 74.02, 52.26, 18.50 ppm.

$[\alpha]_D^{20}$ = –20.0 (c = 1.0, $CHCl_3$) (lit.[4] $[\alpha]_D^{20}$ = –21.0 (c = 1.2, $CHCl_3$)).

The spectroscopic data are in agreement with the literature.[7]

Dimethyl 2,2'-((2-iodo-1,3-phenylene)bis(oxy))(2R,2'R)-bis(3-phenylpropanoate) (28)

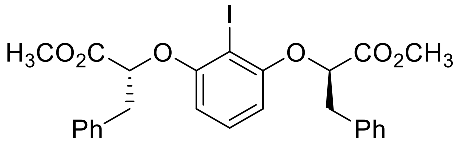

Prepared as per experimental procedure **GP1** using (S)-Methyl 2-hydroxy-3-phenylpropanoate (9.15 g, 50.8 mmol) to give **28** as a colourless solid in 78% yield (8.87 g, 15.8 mmol) after flash column chromatography (*n*-hexane:EtOAc = 80:20).

M.p.: 82 – 86 °C.

^{1}H NMR (400 MHz, $CDCl_3$) δ 7.48 – 7.44 (m, 4H), 7.36 – 7.27 (m, 6H), 7.07 (t, *J* = 8.3 Hz, 1H), 6.27 – 6.22 (m, 2H), 4.84 (dd, *J* = 8.0, 4.5 Hz, 2H), 3.70 (s, 6H), 3.41 – 3.27 (m, 4H) ppm.

^{13}C NMR (101 MHz, $CDCl_3$) δ 171.0, 158.0, 136.0, 129.9, 129.5, 128.4, 127.0, 105.8, 78.8, 52.3, 39.1 ppm.

$[\alpha]_D^{20}$ = +65.0 (c= 0.40, $CHCl_3$).

(2R,2'R)-2,2'-((2-iodo-1,3-phenylene)bis(oxy))dipropionic acid (29)

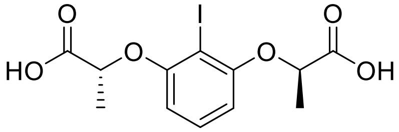

Prepared as per experimental procedure **GP2** using dimethyl 2,2'-((2-iodo-1,3-phenylene)bis(oxy))(2R,2'R)-dipropionate (1224 mg, 3.0 mmol). The obtained compound **29** (1128 mg, 2.97 mmol) was used without any further purification.

^{1}H NMR (500 MHz, CD_3OD) δ 7.18 (t, *J* = 8.3 Hz, 1H), 6.43 (d, *J* = 8.3 Hz, 2H), 4.96 (brs, OH), 4.80 (q, *J* = 6.8 Hz, 2H), 1.65 (d, *J* = 6.8 Hz, 6H) ppm.

^{13}C NMR (126 MHz, CD_3OD) δ 173.88, 158.33, 129.21, 106.16, 79.11, 73.52, 17.61 ppm.

The spectroscopic data are in agreement with the literature.[8]

Dimesityl 2,2'-((2-iodo-1,3-phenylene)bis(oxy))(2R,2'R)-dipropionate (31)

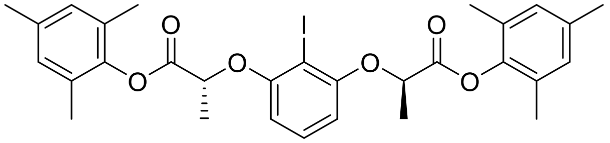

Prepared as per experimental procedure **GP3** using (2R,2'R)-2,2'-((2-iodo-1,3-phenylene)bis(oxy))dipropionic acid (400 mg, 1.052 mmol) and mesitol (358 mg, 2.63 mmol) to give **7** as a colourless solid in 55% yield (356 mg, 0.5786 mmol) after flash column chromatography (*n*-hexane:EtOAc = 80:20).

^{1}H NMR (500 MHz, $CDCl_3$) δ 6.86 – 6.72 (m, 2H), 6.72 – 6.57 (m, 5H), 4.93 – 3.84 (m, 2H), 2.12 (s, 6H), 2.10 (s, 12H), 2.02 (brs, 6H) ppm.

^{13}C NMR (126 MHz, $CDCl_3$) δ 169.23, 149.83, 145.85, 135.28, 129.54, 129.14, 129.02, 122.79, 77.25, 77.00, 76.75, 20.67, 20.36, 20.30, 16.11, 15.75 ppm.

The spectroscopic data are in agreement with the literature.[9]

Bis((1S,2R,5S)-2-isopropyl-5-methylcyclohexyl) 2,2'-((2-iodo-1,3-phenylene)bis(oxy))(2R,2'R)-dipropionate (32)

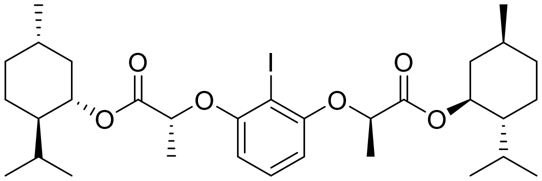

Prepared as per experimental procedure **GP3** using (2R,2'R)-2,2'-((2-iodo-1,3-phenylene)bis(oxy))dipropionic acid (400 mg, 1.052 mmol) and L-Menthol (366 mg, 2.63 mmol) to give **32** as a colourless liquid in 53% yield (356 mg, 0.5786 mmol) after flash column chromatography (*n*-hexane:EtOAc = 80:20).

^{1}H NMR (400 MHz, $CDCl_3$) δ 7.09 (t, *J* = 8.3 Hz, 1H), 6.36 (d, *J* = 8.3 Hz, 2H), 4.80 – 4.66 (m, 4H), 1.84-1.97 (m, 4H), 1.72 – 1.60 (m, 10H), 1.52 – 1.35 (m, 4H), 1.02-1.09 (m, 2H), 0.95 – 0.80 (m, 16H), 0.76 (d, *J* = 7.0 Hz, 6H) ppm.

^{13}C NMR (101 MHz, $CDCl_3$) δ 171.13, 158.16, 129.07, 106.69, 80.76, 75.18, 74.13, 46.67, 40.25, 34.01, 31.19, 26.18, 23.21, 21.86, 20.65, 18.41, 16.09 ppm.

The spectroscopic data are in agreement with literature.[10]

Bis((S)-1-phenylethyl) 2,2'-((2-iodo-1,3-phenylene)bis(oxy))(2R,2'R)-dipropionate (33)

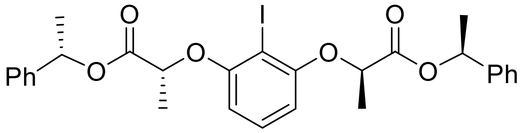

Prepared as per experimental procedure **GP3** using (2R,2'R)-2,2'-((2-iodo-1,3-phenylene)bis(oxy))dipropionic acid (400 mg, 1.052 mmol) and (S)-(−)-1-Phenylethanol (321 mg, 2.63 mmol) to give **9** as a colourless liquid in 51% yield (315 mg, 0.5365 mmol) after flash column chromatography (*n*-hexane:EtOAc = 80:20).

^{1}H NMR (500 MHz, $CDCl_3$) δ 7.30 – 7.22 (m, 10H), 6.86 (t, *J* = 8.3 Hz, 1H), 6.15 (d, *J* = 8.3 Hz, 2H), 5.84 (q, *J* = 6.6 Hz, 2H), 4.70 (q, *J* = 6.8 Hz, 2H), 1.63 (d, *J* = 6.8 Hz, 6H), 1.37 (d, *J* = 6.6 Hz, 6H) ppm.

^{13}C NMR (126 MHz, $CDCl_3$) δ 170.88, 158.01, 140.84, 129.31, 128.50, 128.03, 126.09, 106.54, 80.23, 74.05, 73.33, 21.97, 18.41 ppm.

Dimesityl 2,2'-((2-iodo-1,3-phenylene)bis(oxy))(2R,2'R)-bis(3-phenylpropanoate) (34)

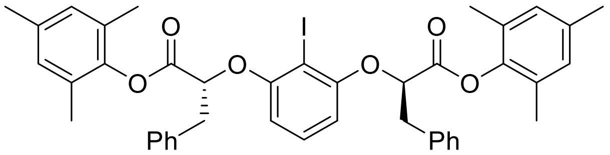

Prepared as per experimental procedure **GP3** using (2R,2'R)-2,2'-((2-iodo-1,3-phenylene)bis(oxy))bis(3-phenylpropanoic acid) (400 mg, 0.7518 mmol) and menthol (256 mg, 1.8796 mmol) to give **10** as a colourless solid in 51% yield (294 mg, 0.3834 mmol) after flash column chromatography (*n*-hexane:EtOAc = 80:20).

^{1}H NMR (400 MHz, $CDCl_3$) δ 7.47 – 7.42 (m, 4H), 7.26 – 7.12 (m, 6H), 7.03 (t, *J* = 8.3 Hz, 1H), 6.72 (s, 4H), 6.41 (d, *J* = 8.4 Hz, 2H), 5.06 (dd, *J* = 8.8, 3.8 Hz, 2H), 3.37-3.51 (m, 4H), 2.15 (s, 6H), 1.81 (s, 12H) ppm.

^{13}C NMR (101 MHz, $CDCl_3$) δ 168.53, 158.10, 145.34, 136.08, 135.62, 130.00, 129.47, 129.31, 129.27, 128.46, 127.09, 106.22, 79.74, 78.68, 39.09, 20.68, 16.27 ppm.

(2R,2'R)-2,2'-((2-iodo-1,3-phenylene)bis(oxy))bis(N,N-diphenylpropanamide) (35)

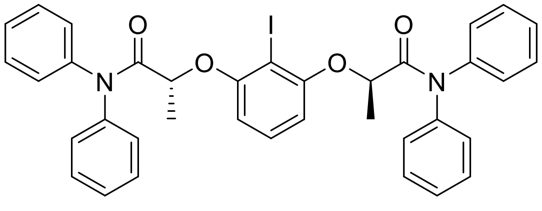

Prepared as per experimental procedure **GP4** using (2R,2'R)-2,2'-((2-iodo-1,3-phenylene)bis(oxy))dipropionic acid (400 mg, 1.052 mmol) and diphenylamine (356 mg, 2.104 mmol) to give **35** as a light yellow solid in 82 % yield (588 mg, 0.8626 mmol) after flash column chromatography (*n*-hexane:EtOAc = 60:40).

^{1}H NMR (300 MHz, $CDCl_3$) δ 7.22-7.35 (m, 20H), 7.14 (t, *J* = 8.2 Hz, 1H), 6.45 – 6.42 (m, 2H), 4.89 (q, *J* = 6.5 Hz, 2H), 1.65 (d, *J* = 6.5 Hz, 6H) ppm.

^{13}C NMR (75 MHz, $CDCl_3$) δ 170.55, 157.97, 141.44, 129.90, 129.06, 128.66, 126.09, 108.79, 83.15, 73.63, 18.07 ppm.

(2R,2'R)-2,2'-((2-iodo-1,3-phenylene)bis(oxy))bis(N-((S)-1-phenylethyl)propanamide) (36)

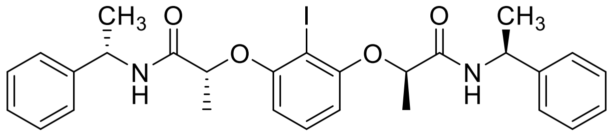

Prepared as per experimental procedure **GP4** using (2R,2'R)-2,2'-((2-iodo-1,3-phenylene)bis(oxy))dipropionic acid (400 mg, 1.052 mmol) and (S)-1-phenylethan-1-amine (255 mg, 2.104 mmol) to give **36** as a colourless solid in 78 % yield (481 mg, 0.8205 mmol) after flash column chromatography (*n*-hexane:EtOAc = 60:40).

^{1}H NMR (400 MHz, $CDCl_3$) δ 7.49 – 7.23 (m, 13H), 6.56 (d, *J* = 7.9 Hz, 2H), 5.32 – 5.10 (m, 2H), 4.86-4.87 (m, 2H), 1.64 (d, *J* = 6.1 Hz, 6H), 1.52 (d, *J* = 6.7 Hz, 6H) ppm.

^{13}C NMR (101 MHz, $CDCl_3$) δ 170.06, 156.86, 142.89, 130.56, 128.71, 127.41, 125.99, 106.91, 80.52, 75.94, 48.65, 22.30, 18.19 ppm.

HRMS (ESI): *m/z* = 609.1221 calcd. for C28H31IN2NaO4+ [M+Na]+, found: 609.1230.

$[\alpha]_D^{20}$ = -81.08 (c = 0.44, $CHCl_3$).

Dimethyl 2,2'-((2-(diacetoxy-l3-iodaneyl)-1,3-phenylene)bis(oxy))(2S,2'S)-dipropionate (27-OAc)

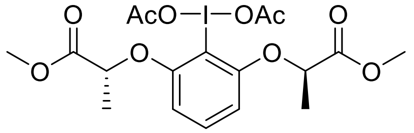

Prepared as per experimental procedure **GP5** using dimethyl 2,2'-((2-iodo-1,3-phenylene)bis(oxy))(2R,2'R)-dipropionate (408 mg, 1.00 mmol) to give **27-OAc** as a colourless solid in 95 % yield (500 mg, 0.95 mmol).

^{1}H NMR (500 MHz, $CDCl_3$) δ 7.40 (t, *J* = 8.4 Hz, 1H), 6.58 (d, *J* = 8.4 Hz, 2H), 4.87 (q, *J* = 6.8 Hz, 2H), 3.76 (s, 6H), 1.99 (s, 6H), 1.69 (d, *J* = 6.8 Hz, 6H) ppm.

^{13}C NMR (126 MHz, $CDCl_3$) δ 176.92, 171.34, 156.67, 135.21, 106.20, 77.25, 77.00, 76.75, 74.47, 52.53, 20.42, 18.49, 18.36 ppm.

Dimethyl 2,2'-((2-(diacetoxy-l3-iodaneyl)-1,3-phenylene)bis(oxy))(2R,2'R)-bis(3-phenylpropanoate) (28-OAc)

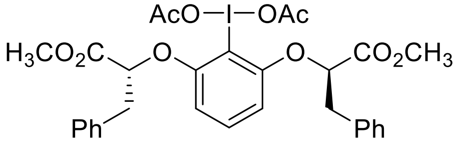

Prepared as per experimental procedure **GP5** using dimethyl 2,2'-((2-iodo-1,3-phenylene)bis(oxy)) (2R,2'R)-bis(3-phenylpropanoate) (560 mg, 1.00 mmol) to give **28-OAc** as a colourless solid in 94 % yield (637 mg, 0.94 mmol).

^{1}H NMR (500 MHz, $CDCl_3$) δ 7.18-7.24 (m, 10H), 7.17 – 7.11 (m, 1H), 6.34 (d, *J* = 8.5 Hz, 2H), 4.86 (t, *J* = 6.2 Hz, 2H), 3.58 (s, 6H), 3.20 (d, *J* = 6.2 Hz, 4H), 1.80 (s, 6H) ppm.

^{13}C NMR (126 MHz, $CDCl_3$) δ 176.94, 169.99, 156.09, 135.31, 129.43, 128.35, 126.92, 105.28, 78.54, 52.31, 38.68, 20.37 ppm.

Dimesityl 2,2'-((2-(diacetoxy-l3-iodaneyl)-1,3-phenylene)bis(oxy))(2S,2'S)-dipropionate (31-OAc)

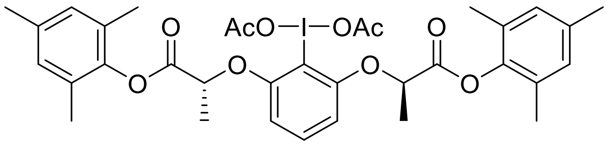

Prepared as per experimental procedure **GP5** using dimesityl 2,2'-((2-iodo-1,3-phenylene)bis(oxy))(2R,2'R)-dipropionate (616 mg, 1.00 mmol) to give 31**-OAc** as a colourless solid in 94 % yield (690 mg, 0.94 mmol).

M.p.: 152 – 156 °C.

^{1}H NMR (400 MHz, $CDCl_3$) δ 7.41 (t, *J* = 8.4 Hz, 1H), 6.79 – 6.72 (m, *J* = 9.8 Hz, 6H), 5.12 (q, *J* = 6.7 Hz, 2H), 2.16 (s, 6H), 1.93 – 1.85 (br, 12H), 1.83 (d, *J* = 6.8 Hz, 6H), 1.62 (s, 6H) ppm.

^{13}C NMR (101 MHz, $CDCl_3$) δ 177.01, 169.01, 156.75, 145.25, 135.82, 135.09, 129.41 (2xC), 107.88, 106.53, 74.60, 20.73, 19.99, 18.74, 16.16 ppm.

$[\alpha]_D^{20}$ = -101.3 (c = 0.4, $CHCl_3$).

The spectroscopic data are in agreement with literature.[11]

Bis((1S,2R,5S)-2-isopropyl-5-methylcyclohexyl) 2,2'-((2-(diacetoxy-l3-iodaneyl)-1,3-phenylene)bis(oxy))(2R,2'R)-dipropionate (32-OAc)

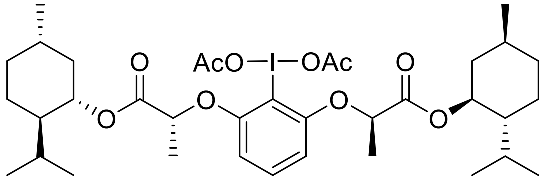

Prepared as per experimental procedure **GP5** using bis((1S,2R,5S)-2-isopropyl-5-methyl cyclohexyl) 2,2'-((2-iodo-1,3-phenylene) bis(oxy))(2R,2'R)-dipropionate (656 mg, 1.00 mmol) to give **32-OAc** as a colourless solid in 96 % yield (630 mg, 0.96 mmol).

^{1}H NMR (400 MHz, $CDCl_3$) δ 7.33 (t, *J* = 8.4 Hz, 1H), 6.53 (d, *J* = 8.4 Hz, 2H), 4.84 – 4.69 (m, 4H), 1.96 (s, 6H), 1.93 – 1.80 (m, 7H), 1.65 (d, *J* = 6.8 Hz, 6H), 1.50 – 1.36 (m, 5H), 0.91 – 0.84 (m, 18H), 0.75 (d, *J* = 7.0 Hz, 6H) ppm.

^{13}C NMR (101 MHz, $CDCl_3$) δ 176.82, 170.42, 156.65, 134.72, 105.86, 75.55, 74.46, 46.67, 40.22, 33.98, 31.20, 26.31, 23.22, 21.85, 20.62, 20.43, 18.19, 16.11 ppm.

$[\alpha]_D^{20}$ = −101.5 (c = 1.3, $CHCl_3$).

Bis((S)-1-phenylethyl) 2,2'-((2-(diacetoxy-l3-iodaneyl)-1,3-phenylene)bis(oxy))(2R,2'R)-dipropionate (33-OAc)

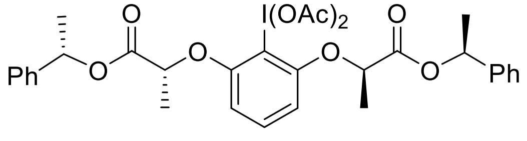

Prepared as per experimental procedure **GP5** using bis((S)-1-phenylethyl) 2,2'-((2-iodo-1,3-phenylene)bis(oxy))(2R,2'R)-dipropionate (588 mg, 1.00 mmol) to give **33-OAc** as a colourless solid in 92 % yield (541 mg, 0.92 mmol).

^{1}H NMR (500 MHz, $CDCl_3$) δ 7.30 – 7.23 (m, 10H), 7.11 (t, *J* = 8.4 Hz, 1H), 6.34 (d, *J* = 8.3 Hz, 2H), 5.86 (q, *J* = 13.3, 6.7 Hz, 2H), 4.76 (q, *J* = 6.8 Hz, 2H), 1.83 (s, 6H), 1.59 (d, *J* = 6.8 Hz, 6H), 1.39 (d, *J* = 6.6 Hz, 6H) ppm.

^{13}C NMR (126 MHz, $CDCl_3$) δ 177.05, 170.09, 156.60, 140.82, 134.94, 128.65, 128.20, 126.03, 125.41, 106.01, 74.51, 73.63, 21.99, 20.45, 18.22 ppm.

Dimesityl 2,2'-((2-(diacetoxy-l3-iodaneyl)-1,3-phenylene)bis(oxy))(2R,2'R)-bis(3-phenylpropanoate) (34-OAc)

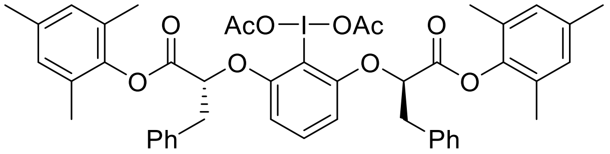

Prepared as per experimental procedure **GP5** using dimesityl 2,2'-((2-iodo-1,3 phenylene)bis(oxy))(2R,2'R)-bis(3-phenyl propanoate) (768 mg, 1.00 mmol) to give **34-OAc** as a colourless solid in 90 % yield (691 mg, 0.90 mmol).

^{1}H NMR (400 MHz, $CDCl_3$) δ 7.37 – 7.30 (m, 3H), 7.25 – 7.14 (m, 8H), 6.76 – 6.63 (m, 8H), 5.24 – 5.15 (m, 2H), 3.51 – 3.38 (m, 4H), 2.15 (s, 6H), 1.90 – 1.64 (m, 12H), 1.50 (s, *J* = 5.1 Hz, 6H) ppm.

^{13}C NMR (101 MHz, $CDCl_3$) δ 176.99, 167.97, 156.48, 145.25, 135.80, 135.58, 134.78, 129.77, 129.36, 129.34, 128.58, 127.21, 106.06, 78.96, 39.02, 20.67, 19.94, 16.12 ppm.

(2,6-bis(((S)-1-(diphenylamino)-1-oxopropan-2-yl)oxy)phenyl)-l3-iodanediyl diacetate (35-OAC)

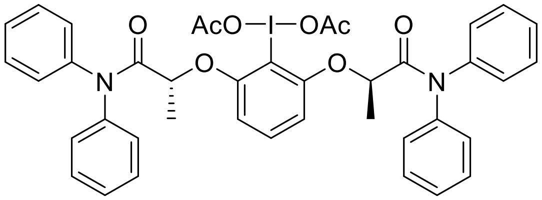

Prepared as per experimental procedure **GP5** using (2R,2'R)-2,2'-((2-iodo-1,3-phenylene) bis(oxy))bis(N,N-diphenylpropanamide) (682 mg, 1.00 mmol) to give **35-OAc** as a colourless solid in 95 % yield (760 mg, 0.95 mmol).

^{1}H NMR (400 MHz, $CDCl_3$) δ 7.38 – 7.11 (m, 21H), 6.47 (d, *J* = 8.4 Hz, 2H), 4.87 (q, *J* = 6.5 Hz, 2H), 1.78 (s, 6H), 1.50 (d, *J* = 6.5 Hz, 6H) ppm.

(2,6-bis(((R)-1-oxo-1-(((S)-1-phenylethyl)amino)propan-2-yl)oxy)phenyl)-l3-iodanediyl diacetate (36-OAC)

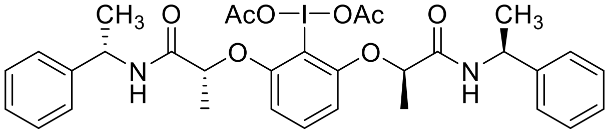

Prepared as per experimental procedure **GP5** using (2R,2'R)-2,2'-((2-iodo-1,3-phenylene)bis(oxy))bis(N-((S)-1-phenylethyl)propanamide) (586 mg, 1.00 mmol) to give **36-OAc** as a colourless solid in 95 % yield (662 mg, 0.94 mmol).

^{1}H NMR (500 MHz, $CDCl_3$) δ 7.46 – 7.42 (m, 1H), 7.26 – 7.15 (m, 10H), 6.66 – 6.64 (m, 2H), 5.11 – 5.05 (m, 2H), 4.84 (q, *J* = 6.7 Hz,2), 1.61 (d, *J* = 6.7 Hz, 6H), 1.57 (s, 6H), 1.20 (d, *J* = 7.0 Hz, 6H) ppm.

^{13}C NMR (126 MHz, $CDCl_3$) δ 176.58, 170.01, 155.61, 142.55, 135.79, 128.61, 127.34, 125.99, 106.09, 76.33, 47.88, 21.05, 19.88, 18.64 ppm.

$[\alpha]_D^{20}$ = -98.4 (c = 0.25, $CHCl_3$).

1.7 NMR data

2-(2-iodophenyl)propan-2-ol (**23**)

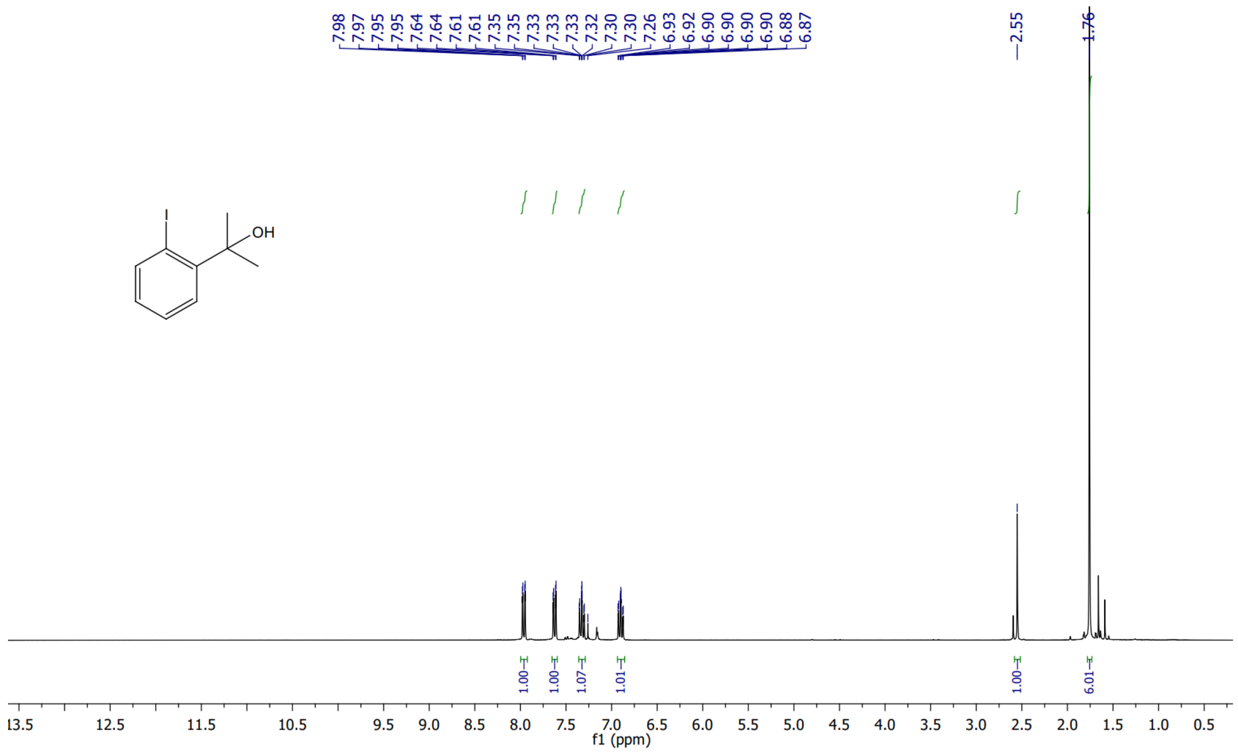

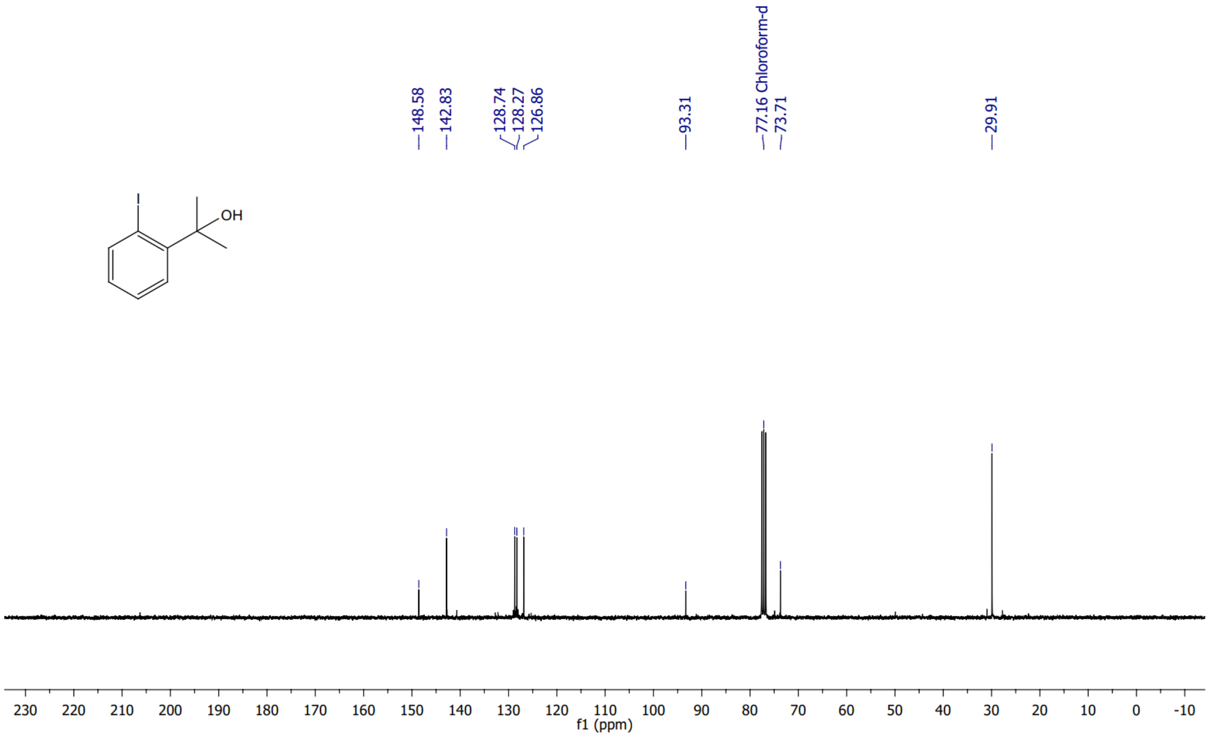

3,3-dimethyl-1λ^3-benzo[d][1,2]iodaoxol-1(3*H*)-yl acetate (24)

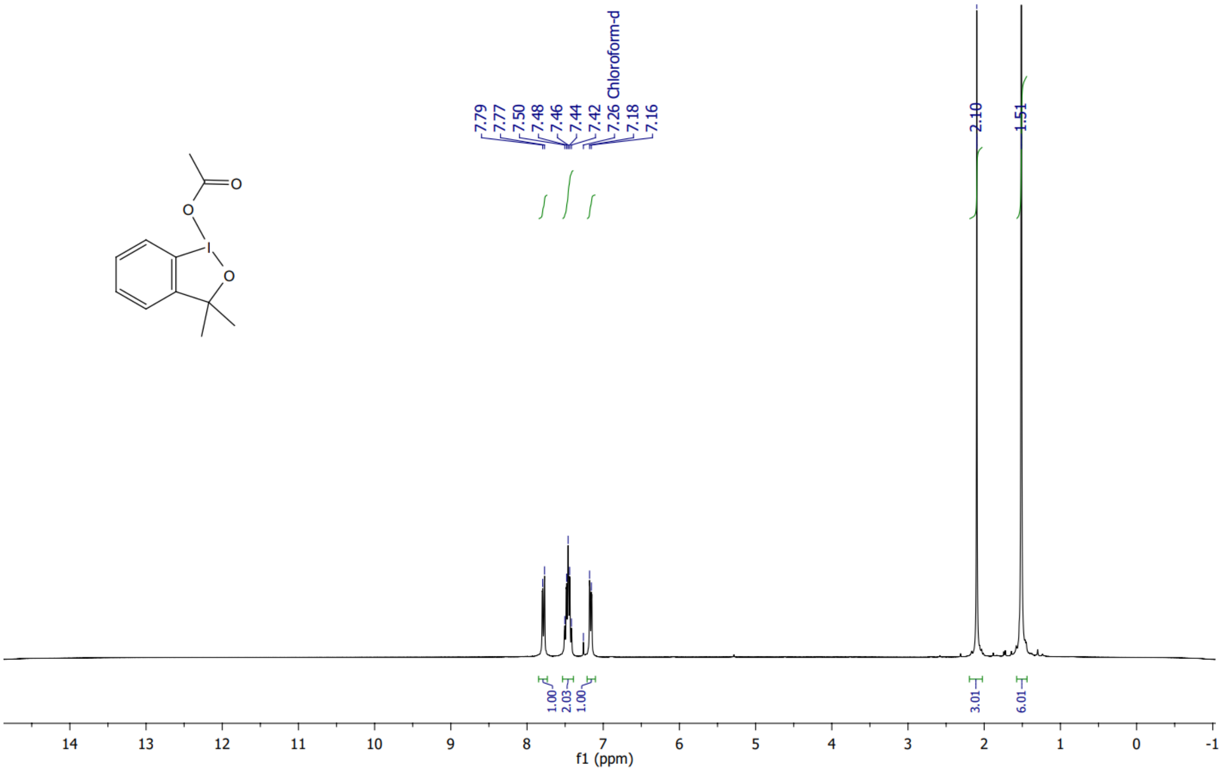

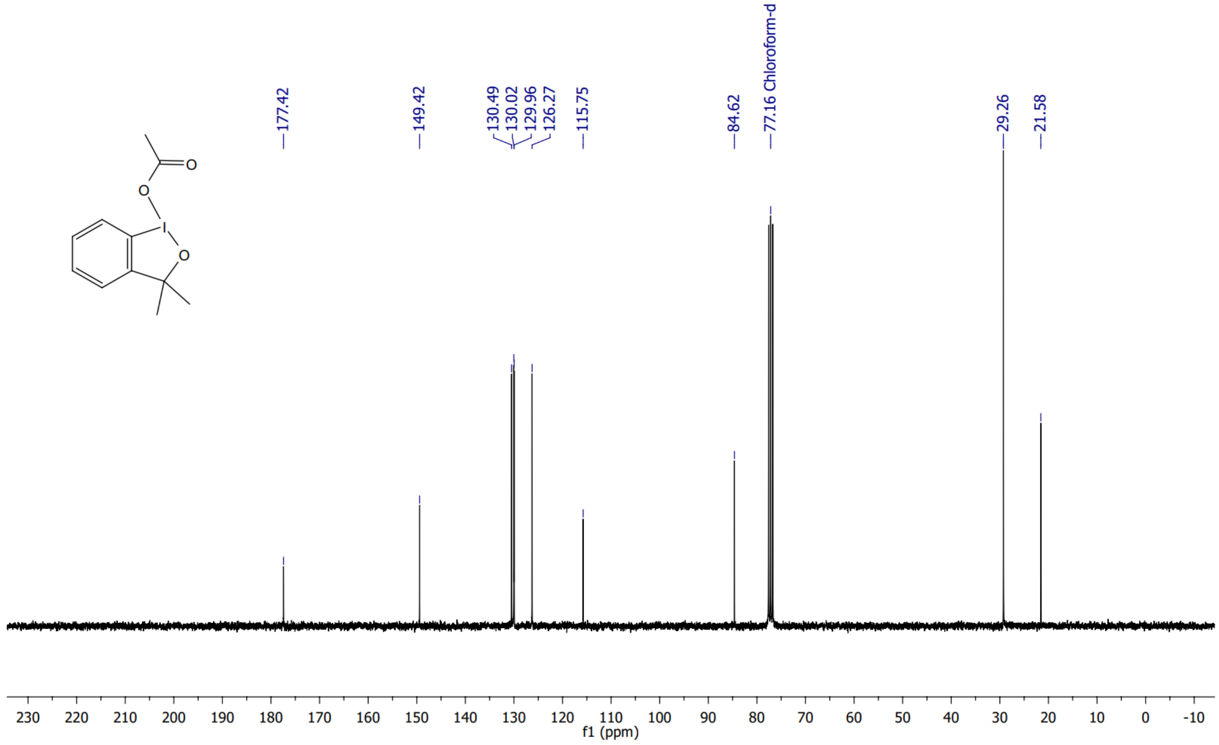

2-Iodobenzene-1,3-diol (26)
^{1}H NMR (300 MHz, MeOD)

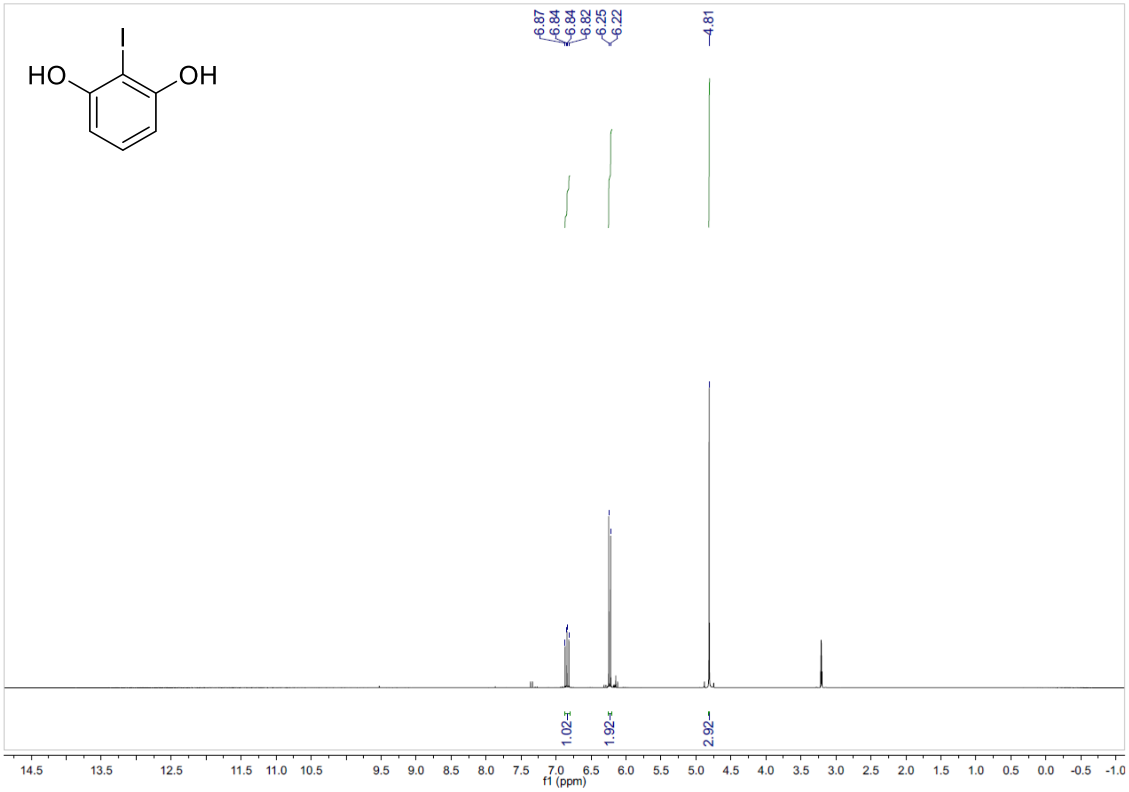

^{13}C NMR (75 MHz, MeOD)

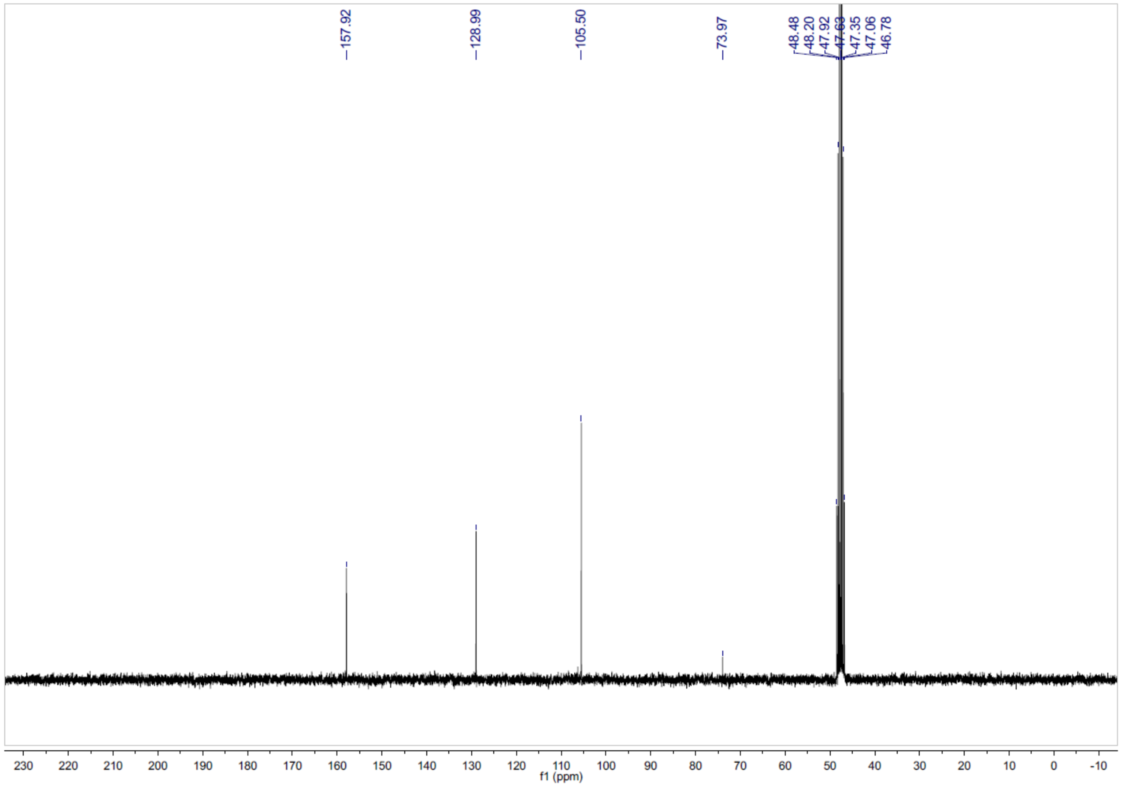

Dimethyl 2,2'-((2-iodo-1,3-phenylene)bis(oxy))(2R,2'R)-dipropionate (27)

^{1}H NMR (300 MHz, $CDCl_3$)

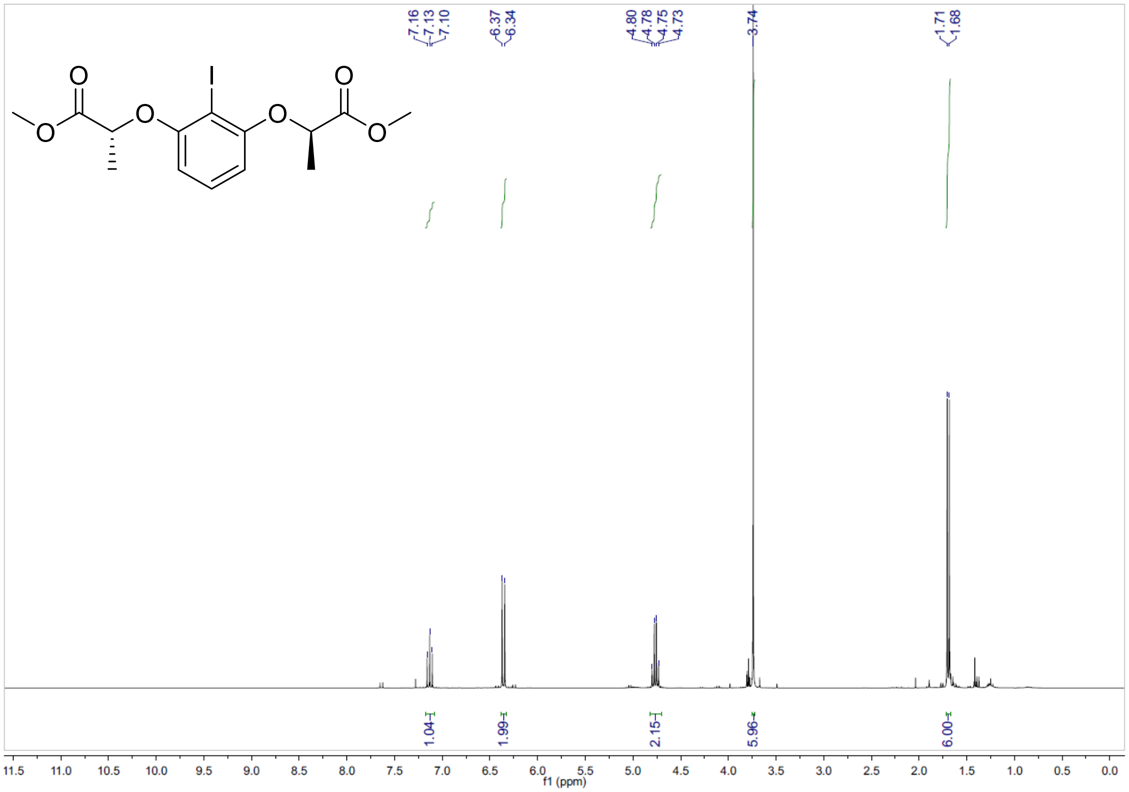

^{13}C NMR (75 MHz, $CDCl_3$)

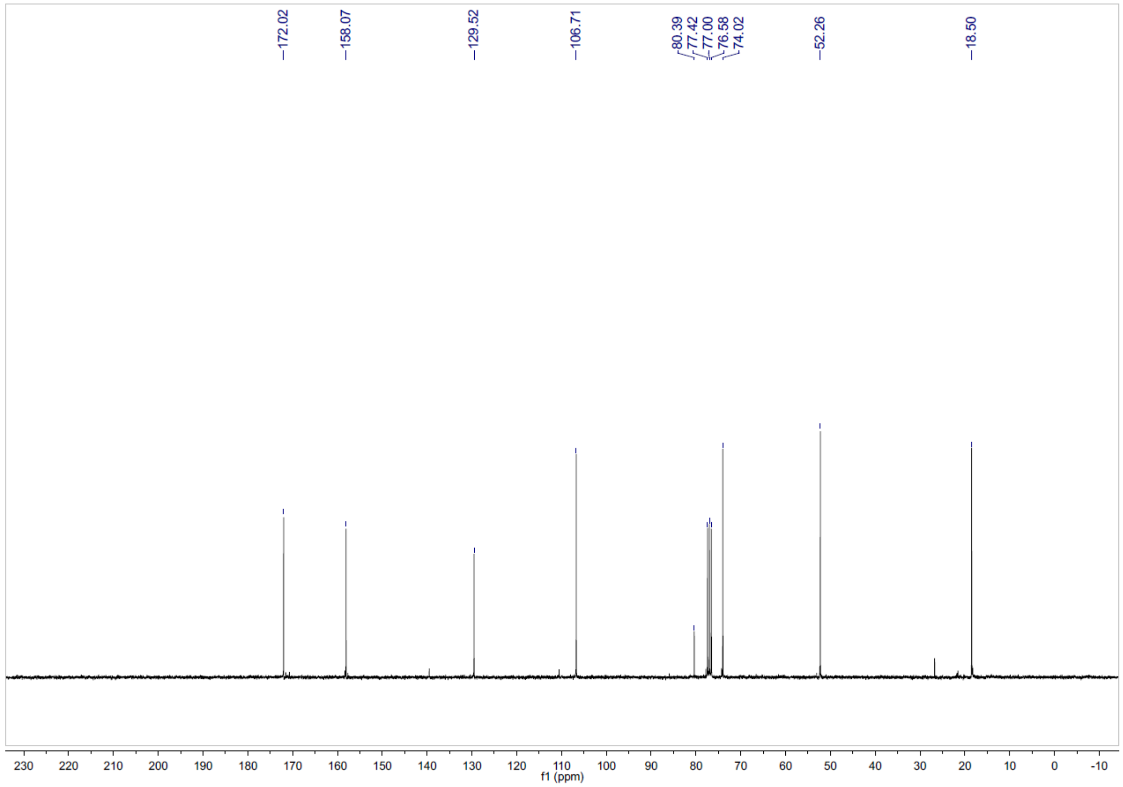

Dimethyl 2,2'-((2-iodo-1,3-phenylene)bis(oxy))(2R,2'R)-bis(3-phenylpropanoate) (28)

^{1}H NMR (400 MHz, $CDCl_3$)

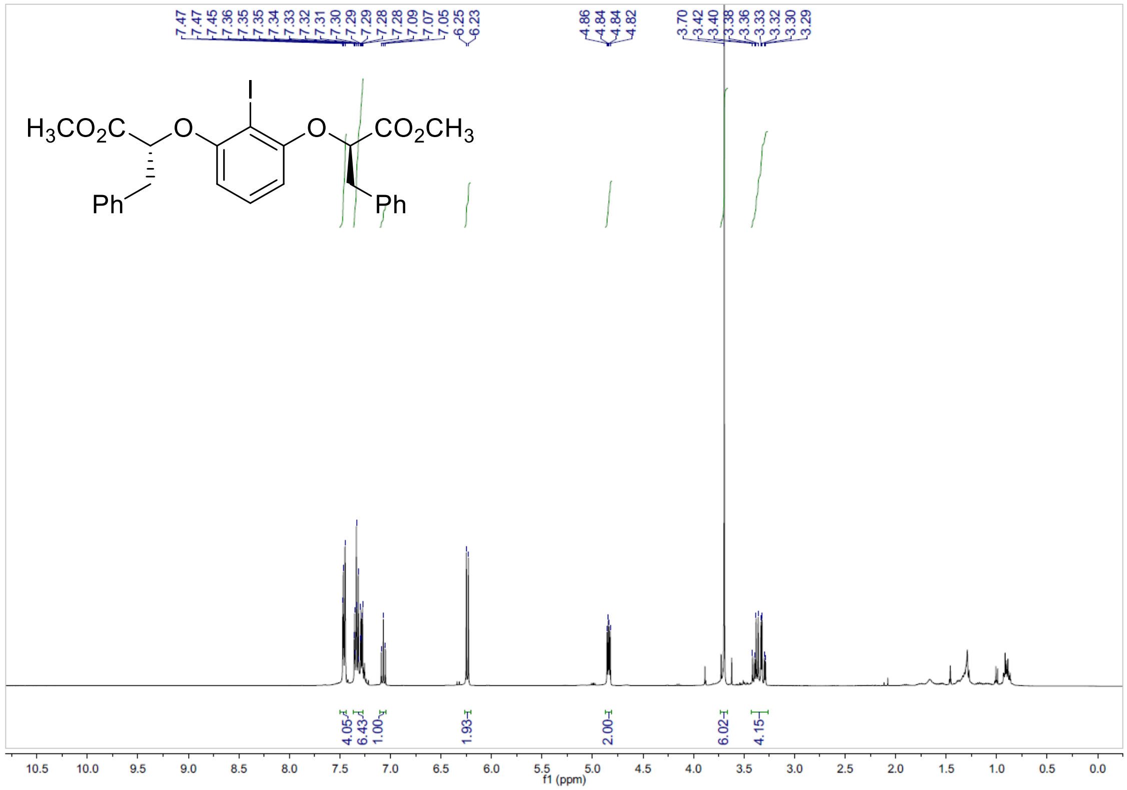

^{13}C NMR (101 MHz, $CDCl_3$)

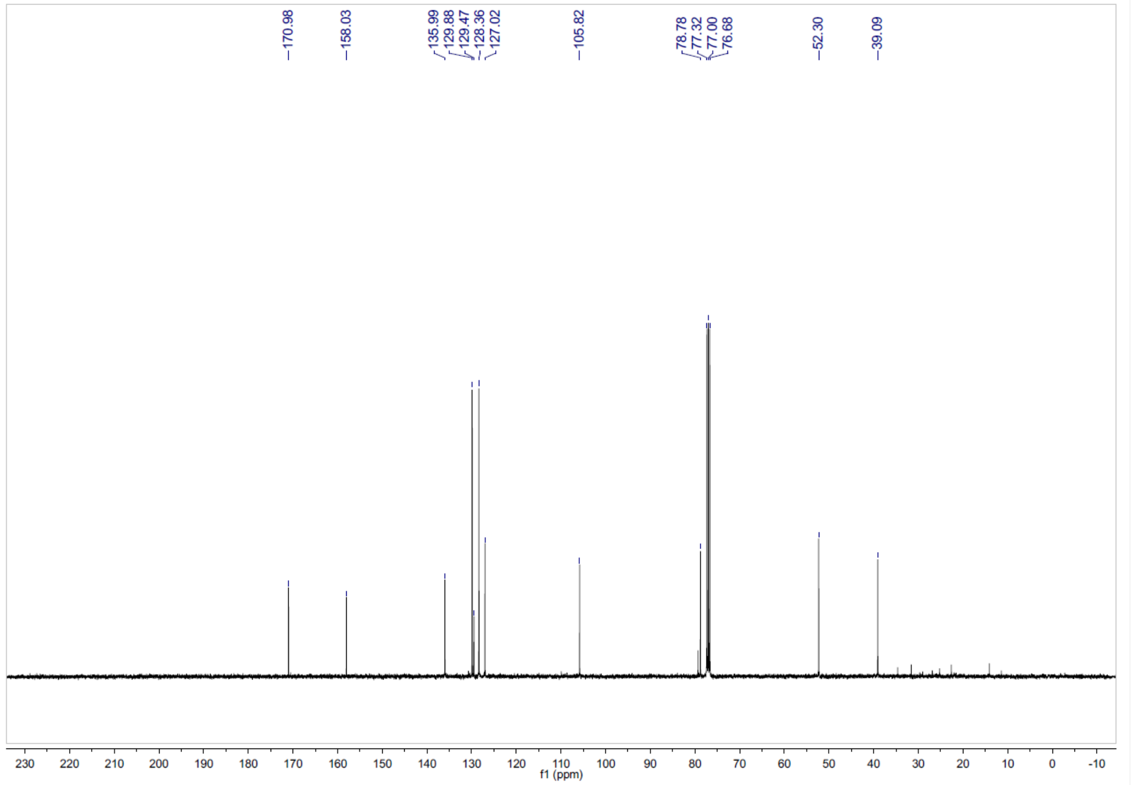

(2R,2'R)-2,2'-((2-iodo-1,3-phenylene)bis(oxy))dipropionic acid (29)

^{1}H NMR (500 MHz, CD$_3$OD)

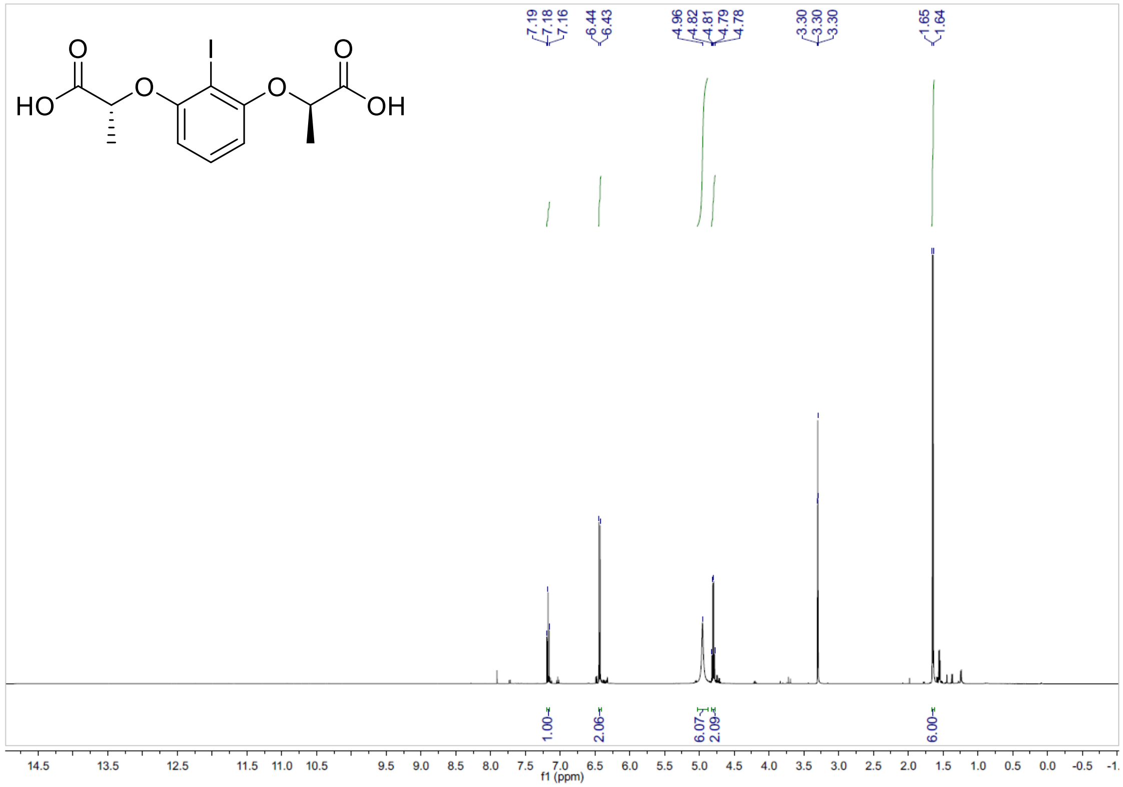

^{13}C NMR (126 MHz, CD$_3$OD)

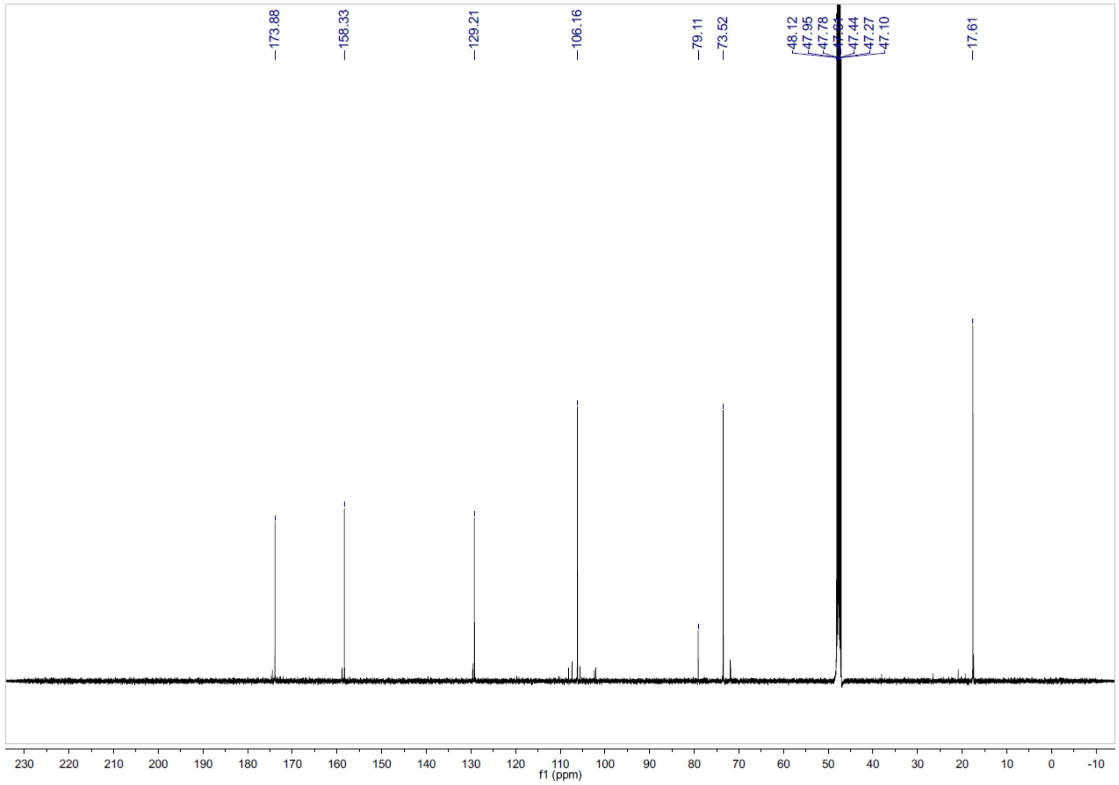

Dimesityl 2,2'-((2-iodo-1,3-phenylene)bis(oxy))(2R,2'R)-dipropionate (31)
^{1}H NMR (500 MHz, CDCl$_3$)

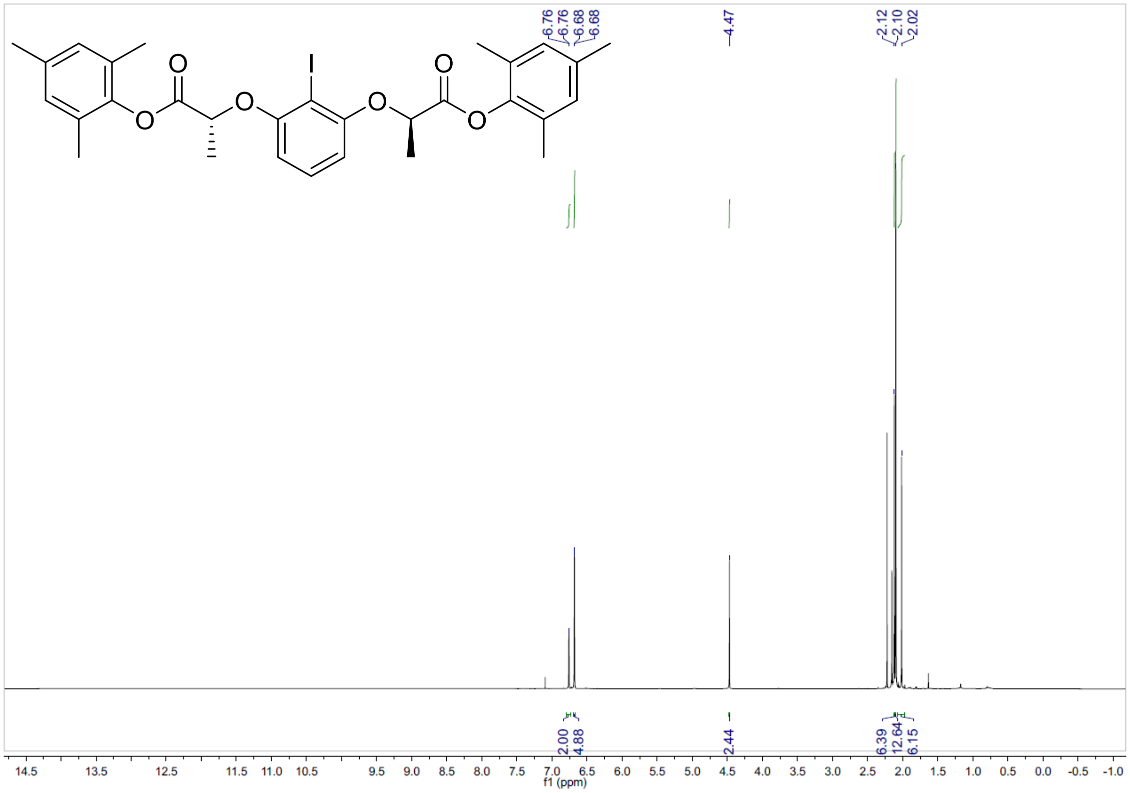

^{13}C NMR (126 MHz, CDCl$_3$)

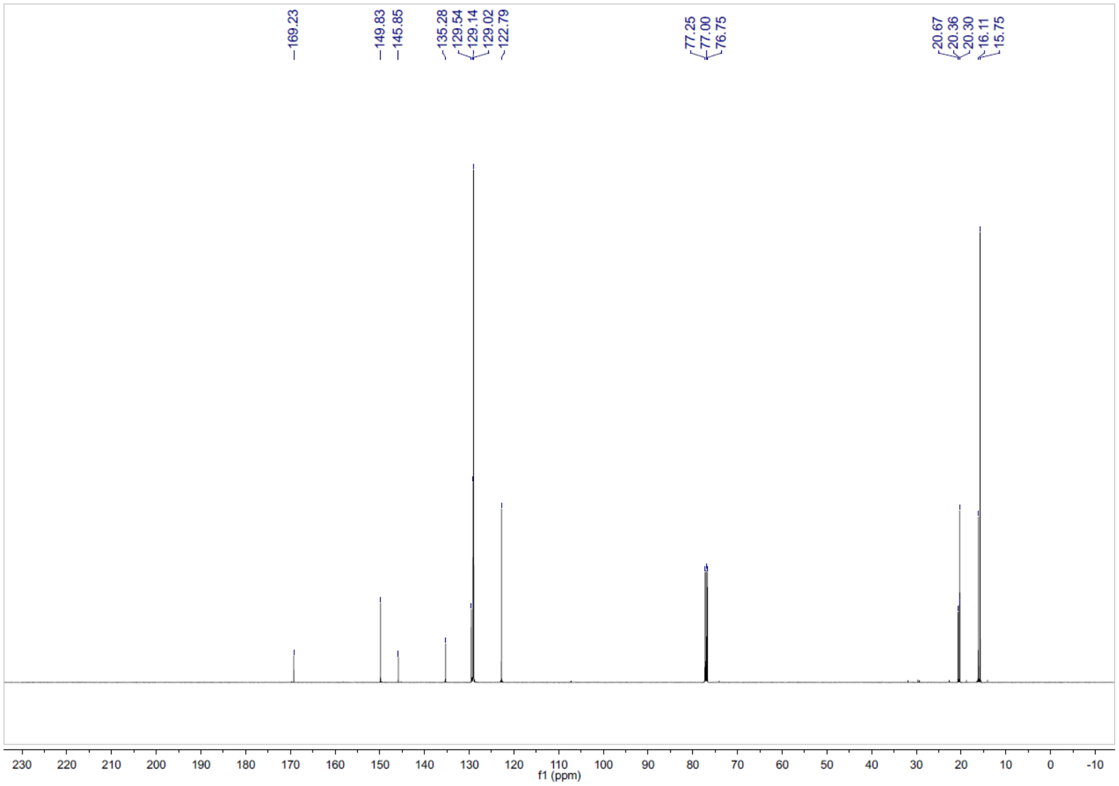

Bis((1S,2R,5S)-2-isopropyl-5-methylcyclohexyl) 2,2'-((2-iodo-1,3-phenylene)bis(oxy))(2R,2'R)-dipropionate (32)

^{1}H NMR (400 MHz, $CDCl_3$)

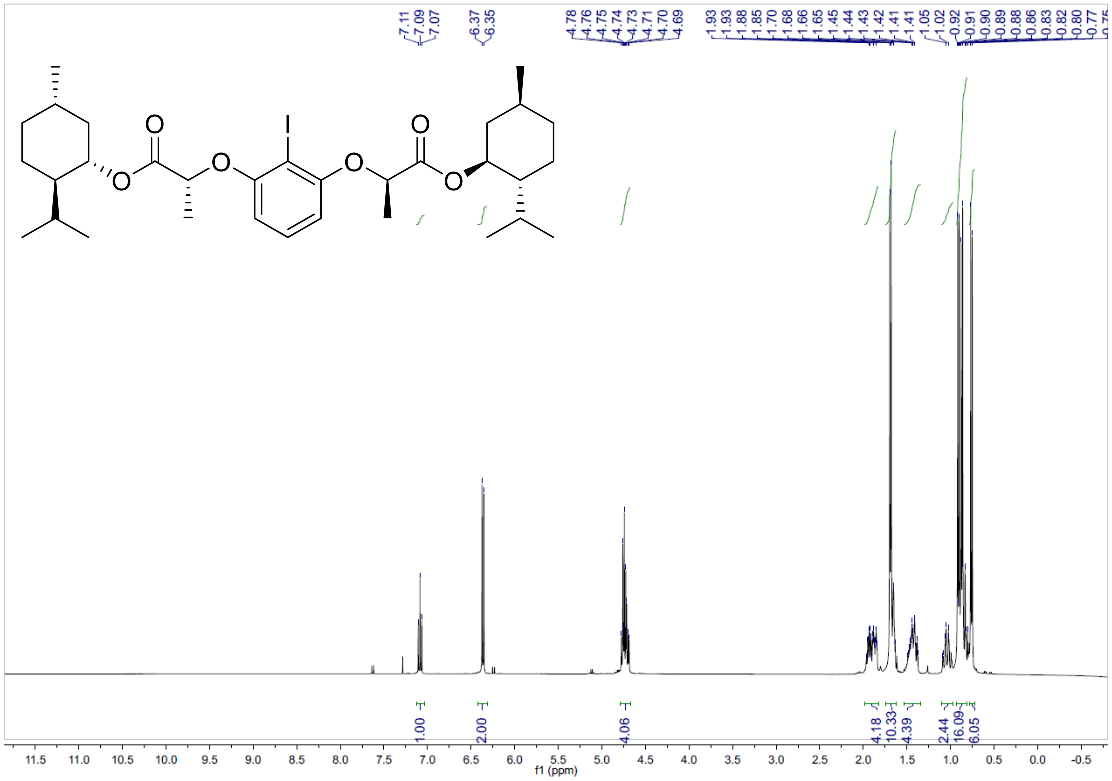

^{13}C NMR (101 MHz, $CDCl_3$)

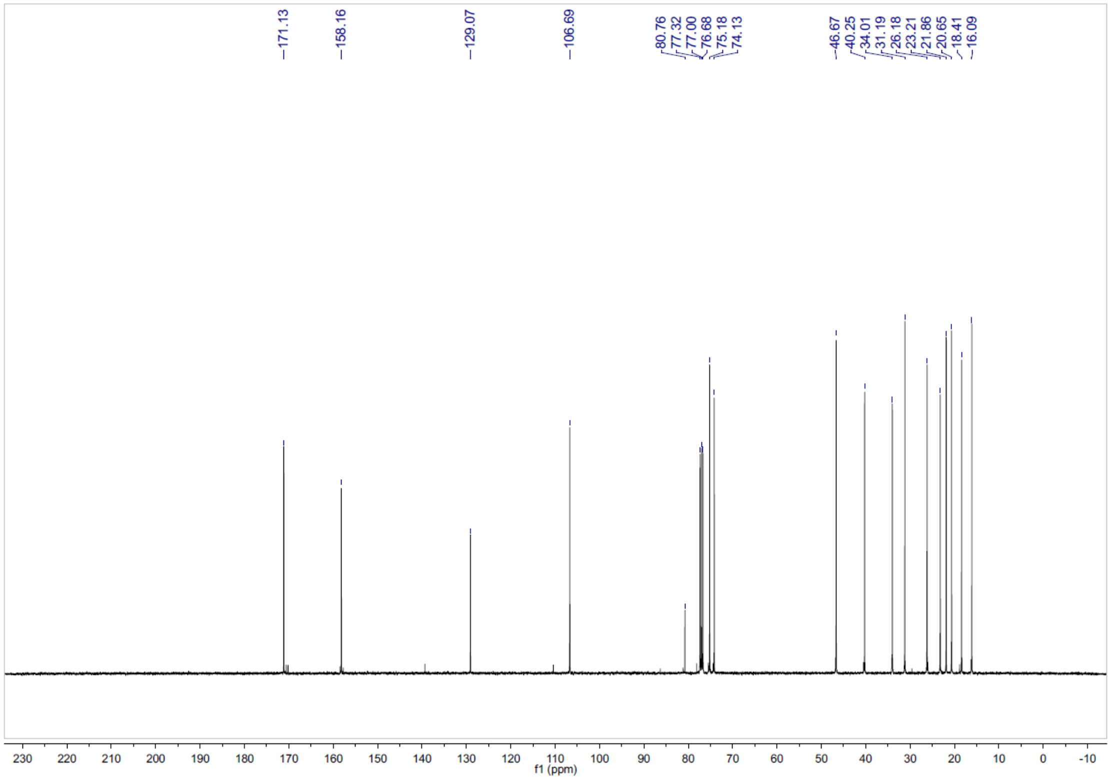

Bis((S)-1-phenylethyl) 2,2'-((2-iodo-1,3-phenylene)bis(oxy))(2R,2'R)-dipropionate (33)

^{1}H NMR (500 MHz, $CDCl_3$)

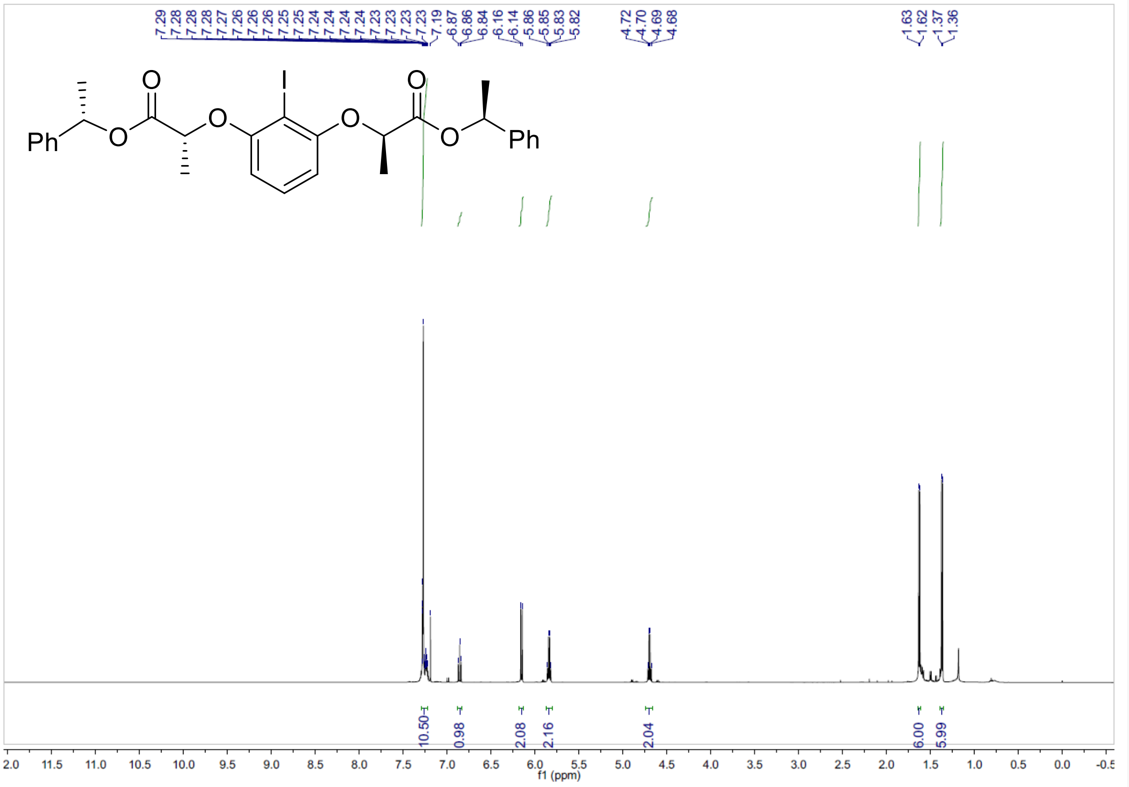

^{13}C NMR (126 MHz, $CDCl_3$)

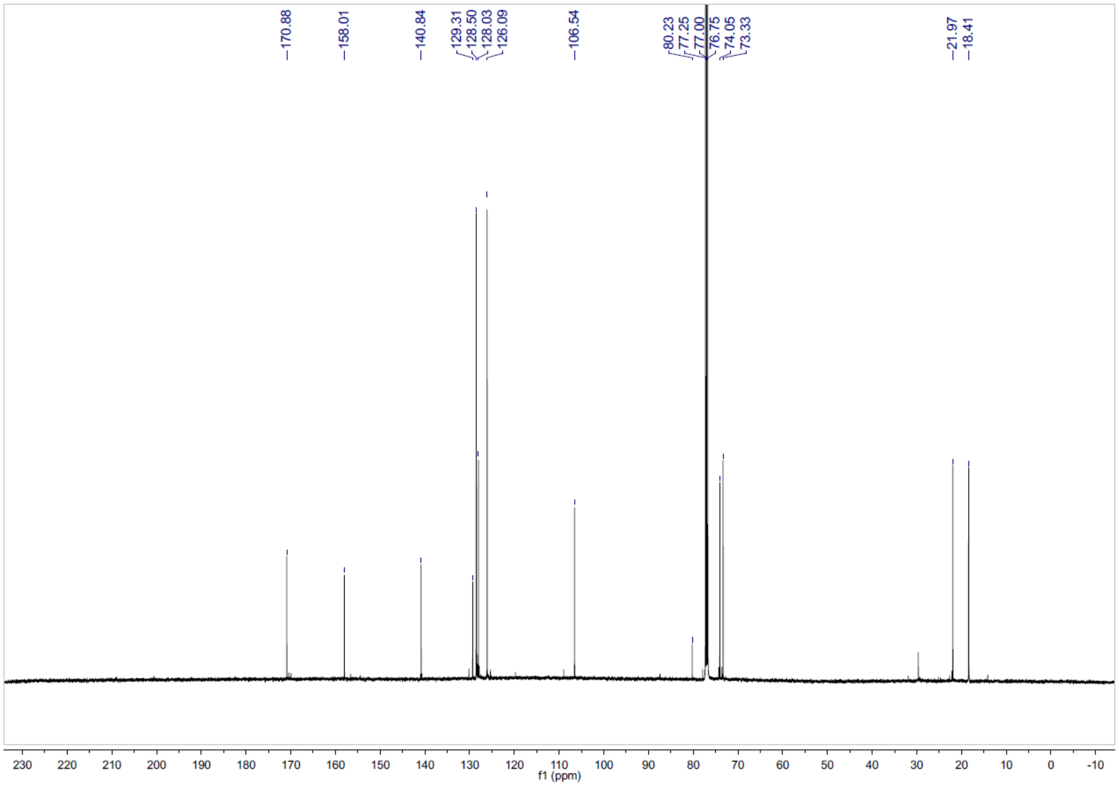

Dimesityl 2,2'-((2-iodo-1,3-phenylene)bis(oxy))(2R,2'R)-bis(3-phenylpropanoate) (34)
^{1}H NMR (400 MHz, CDCl3)

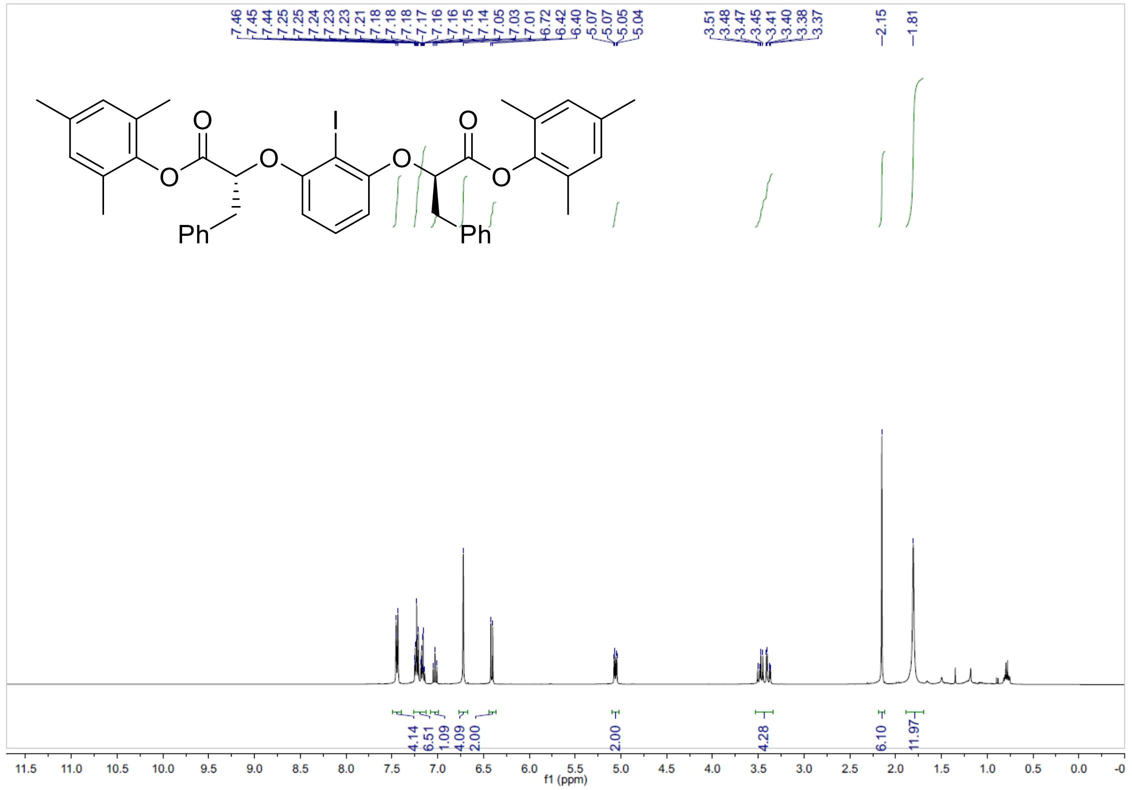

^{13}C NMR (101 MHz, CDCl3)

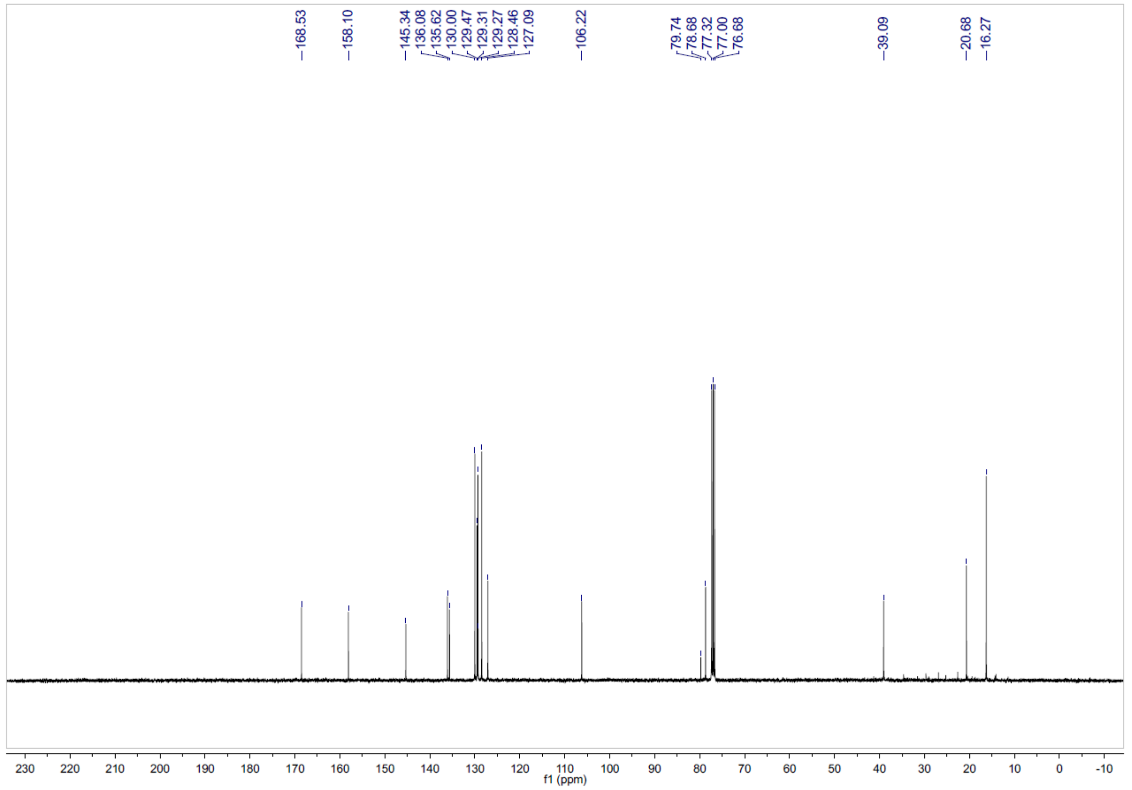

(2R,2'R)-2,2'-((2-iodo-1,3-phenylene)bis(oxy))bis(N,N-diphenylpropanamide) (35)

^{1}H NMR (300 MHz, $CDCl_3$)

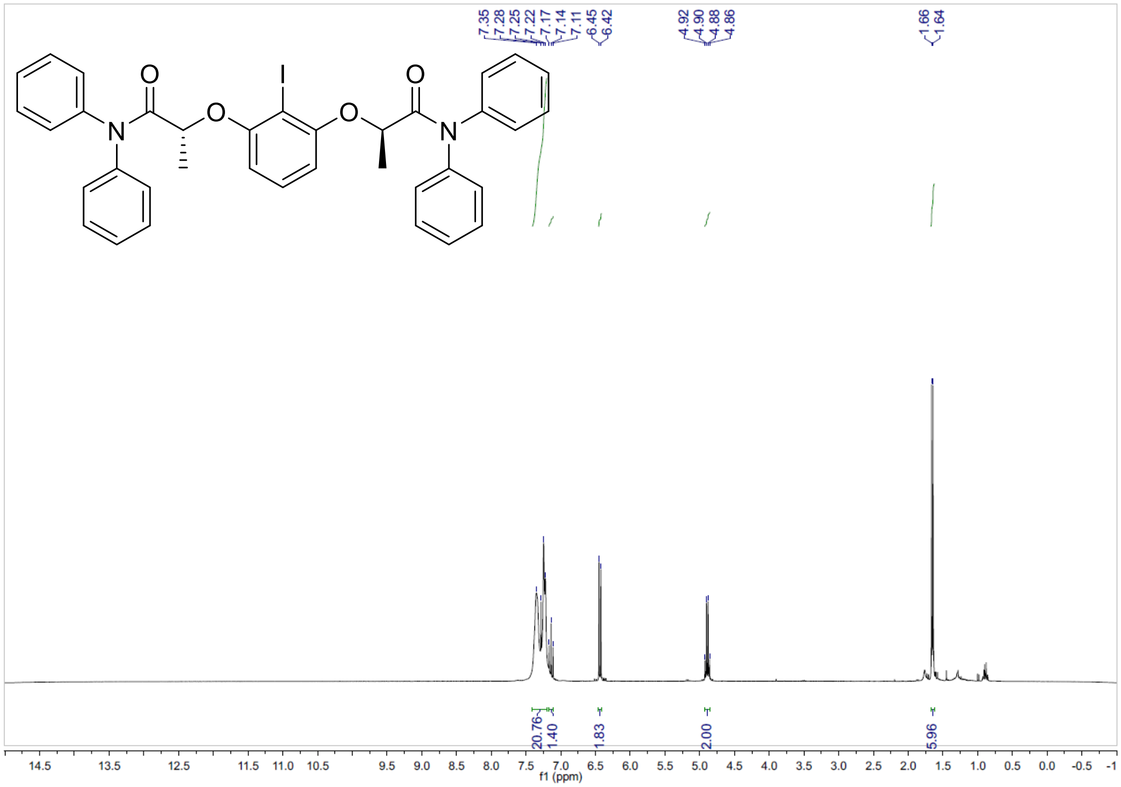

^{13}C NMR (75 MHz, $CDCl_3$)

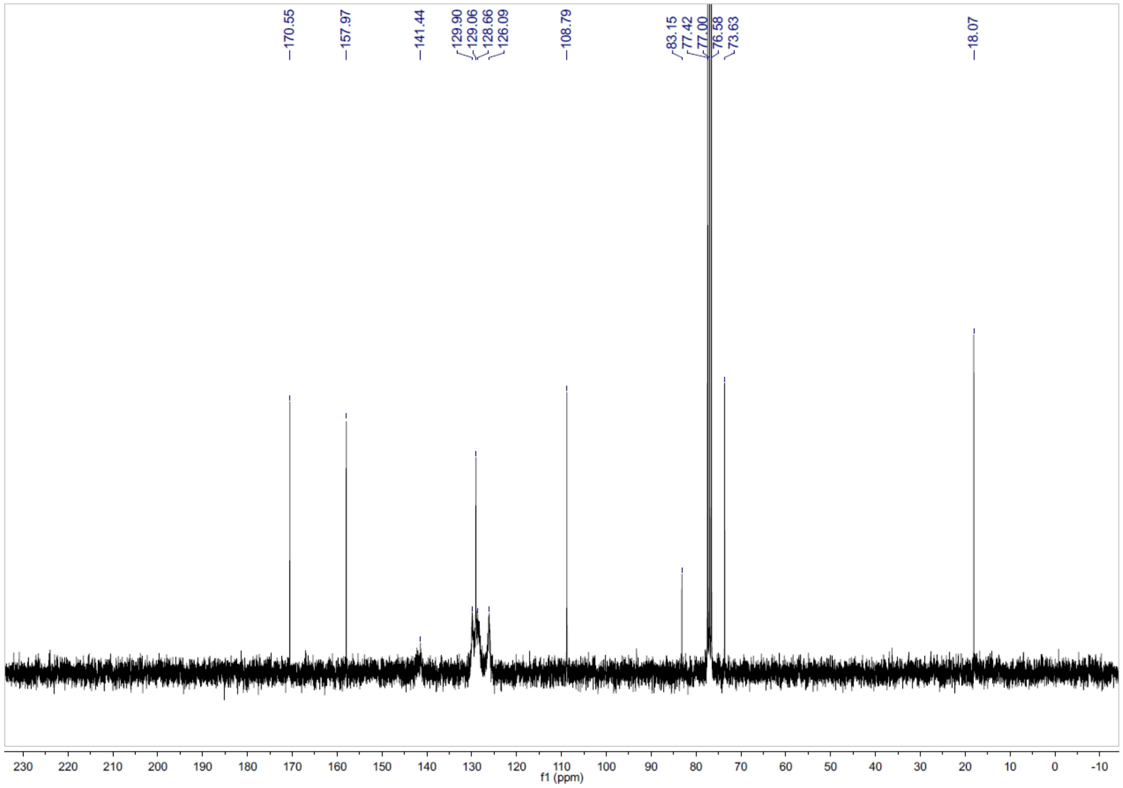

(2R,2'R)-2,2'-((2-iodo-1,3-phenylene)bis(oxy))bis(N-((S)-1-phenylethyl)propanamide) (36)

^{1}H NMR (400 MHz, $CDCl_3$)

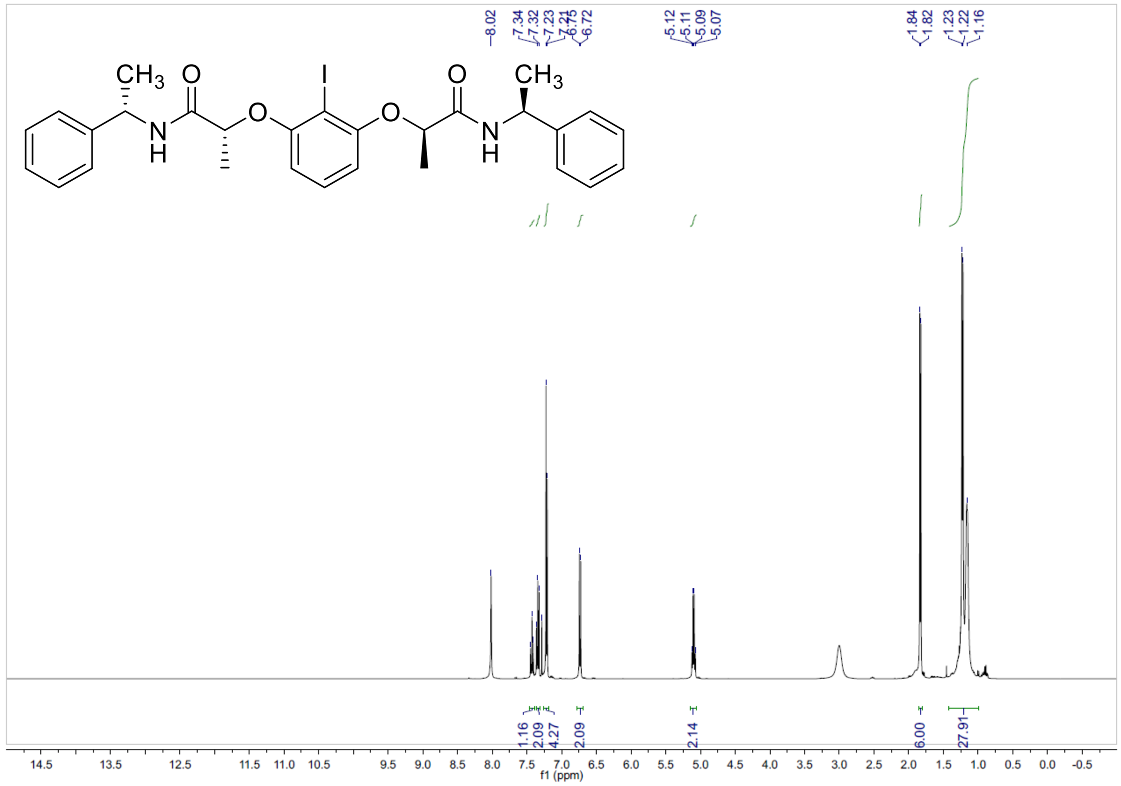

^{13}C NMR (101 MHz, $CDCl_3$)

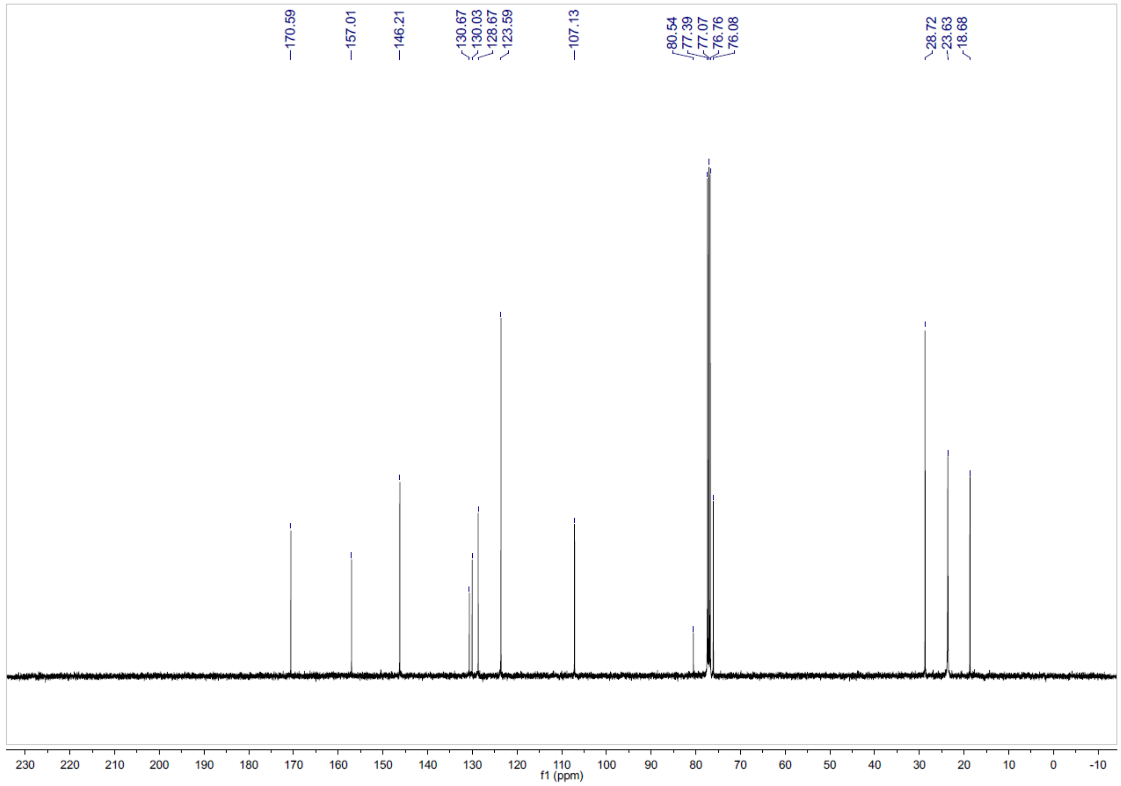

Dimethyl 2,2'-((2-(diacetoxy-l3-iodaneyl)-1,3-phenylene)bis(oxy))(2S,2'S)-dipropionate (27-OAc)

^{1}H NMR (500 MHz, CDCl3)

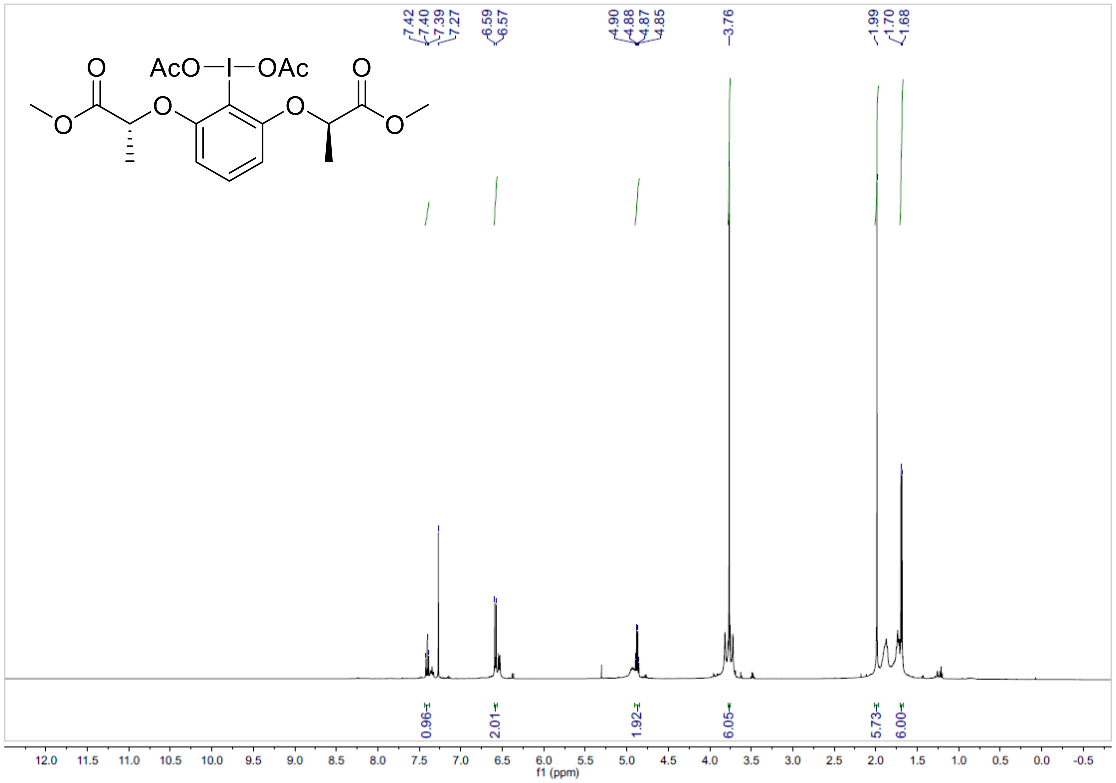

^{13}C NMR (126 MHz, CDCl3)

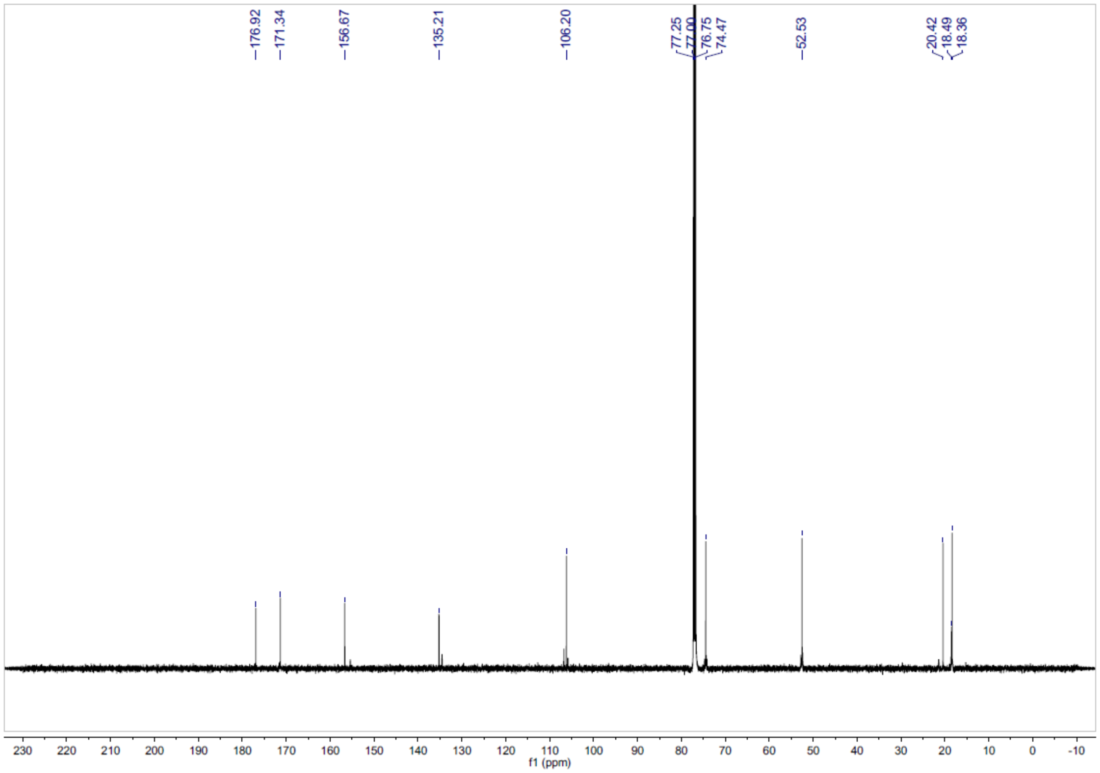

Dimethyl 2,2'-((2-(diacetoxy-l3-iodaneyl)-1,3-phenylene)bis(oxy))(2R,2'R)-bis(3-phenylpropanoate) (28-OAc)

^{1}H NMR (500 MHz, $CDCl_3$)

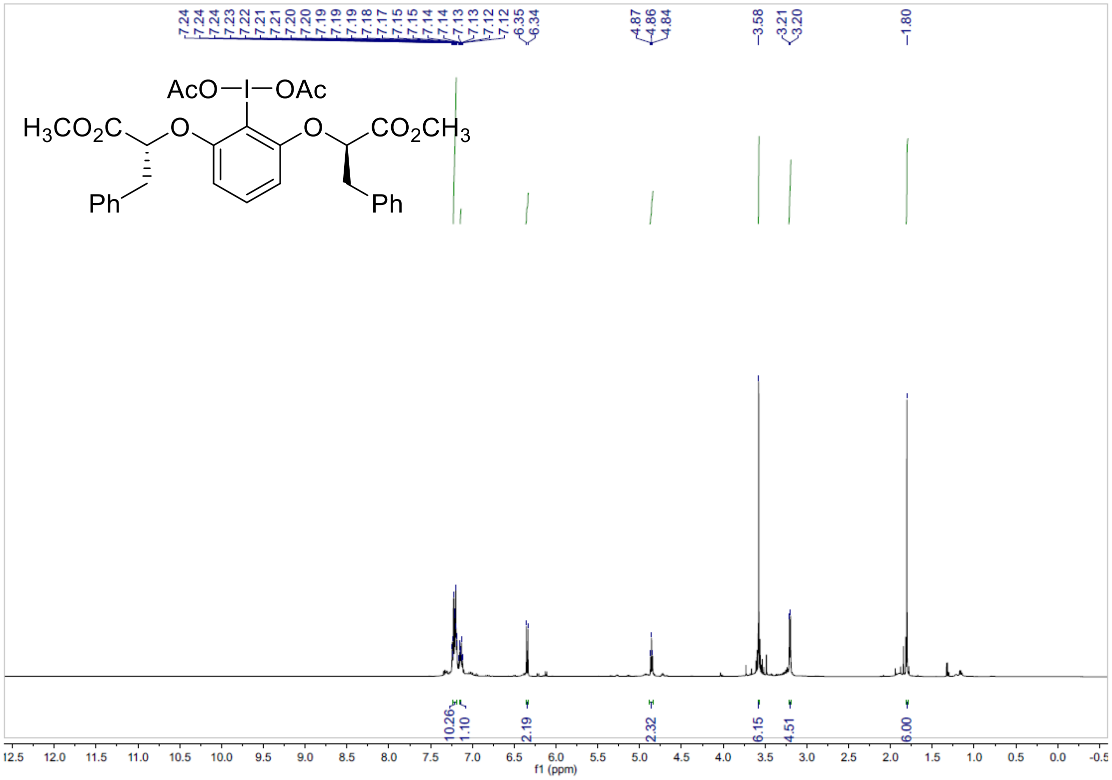

^{13}C NMR (126 MHz, $CDCl_3$)

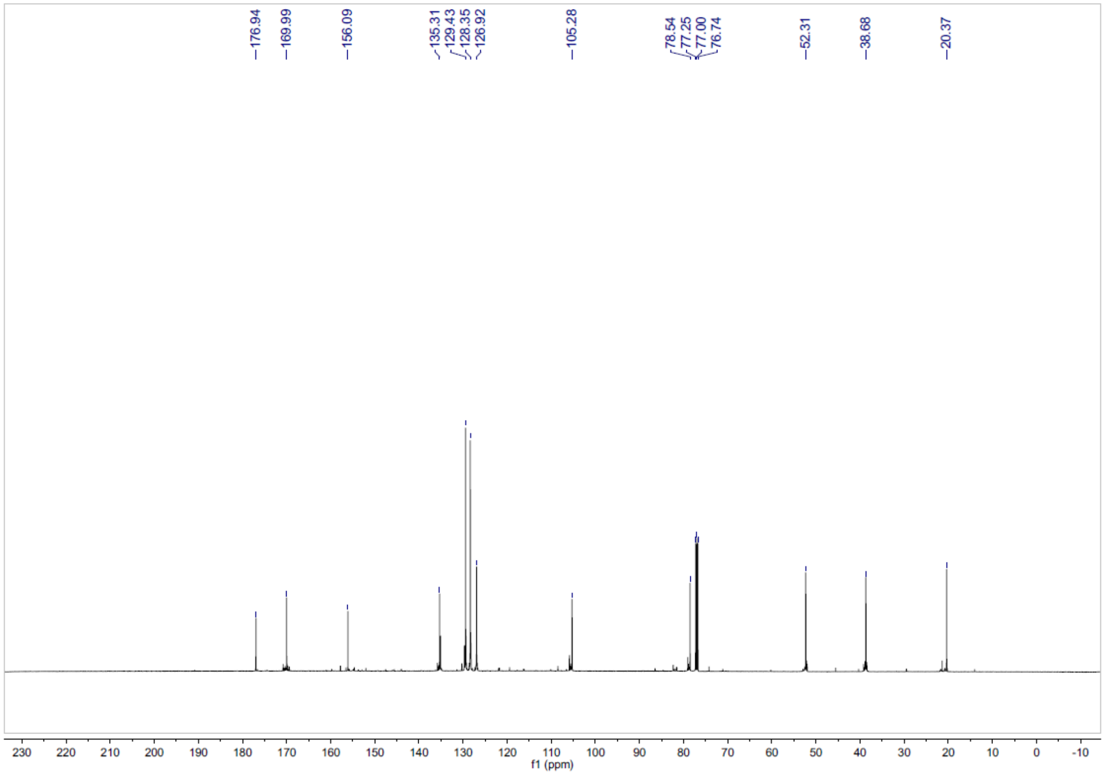

Dimesityl 2,2'-((2-(diacetoxy-l3-iodaneyl)-1,3-phenylene)bis(oxy))(2S,2'S)-dipropionate (31-OAc)

^{1}H NMR (400 MHz, $CDCl_3$)

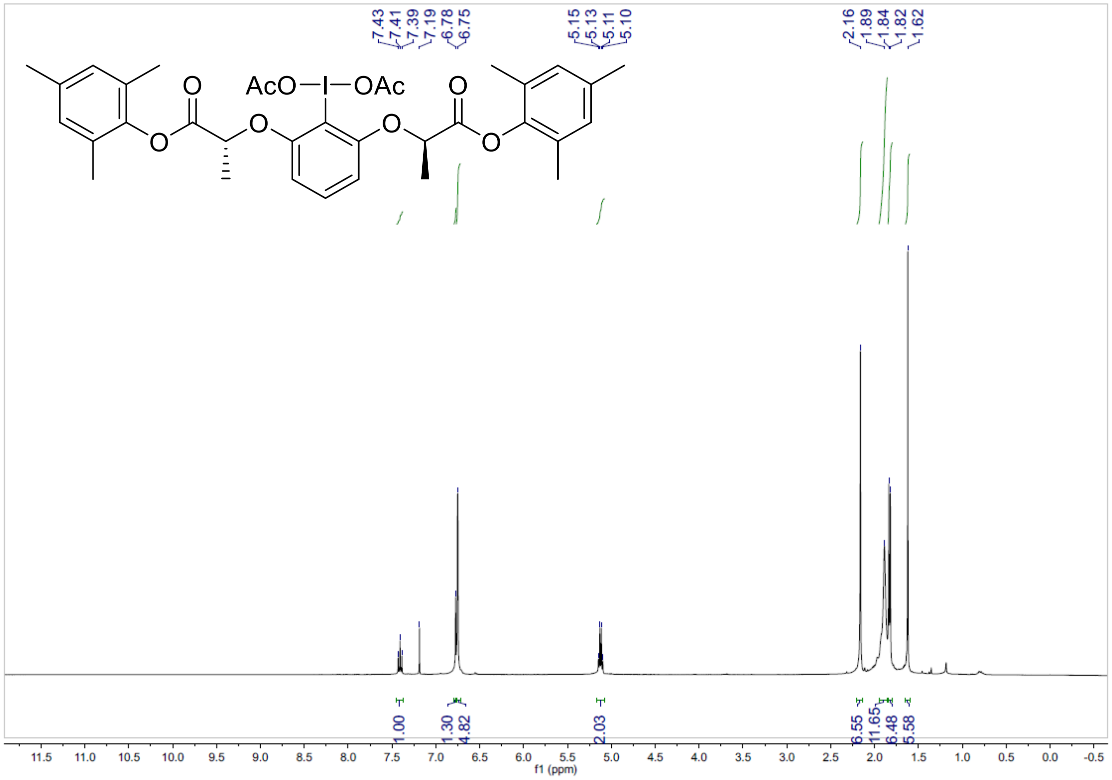

^{13}C NMR (101 MHz, $CDCl_3$)

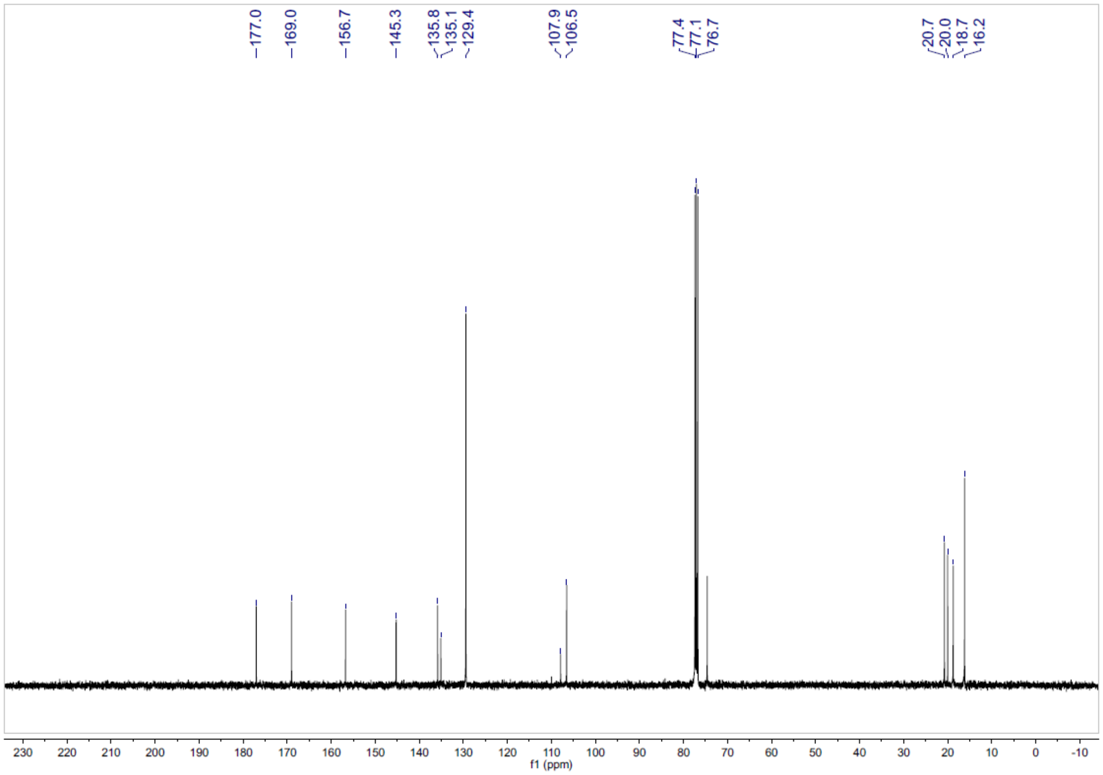

Bis((1S,2R,5S)-2-isopropyl-5-methylcyclohexyl) 2,2'-((2-(diacetoxy-l3-iodaneyl)-1,3-phenylene)bis(oxy))(2R,2'R)-dipropionate (32-OAc)

^{1}H NMR (400 MHz, CDCl$_3$)

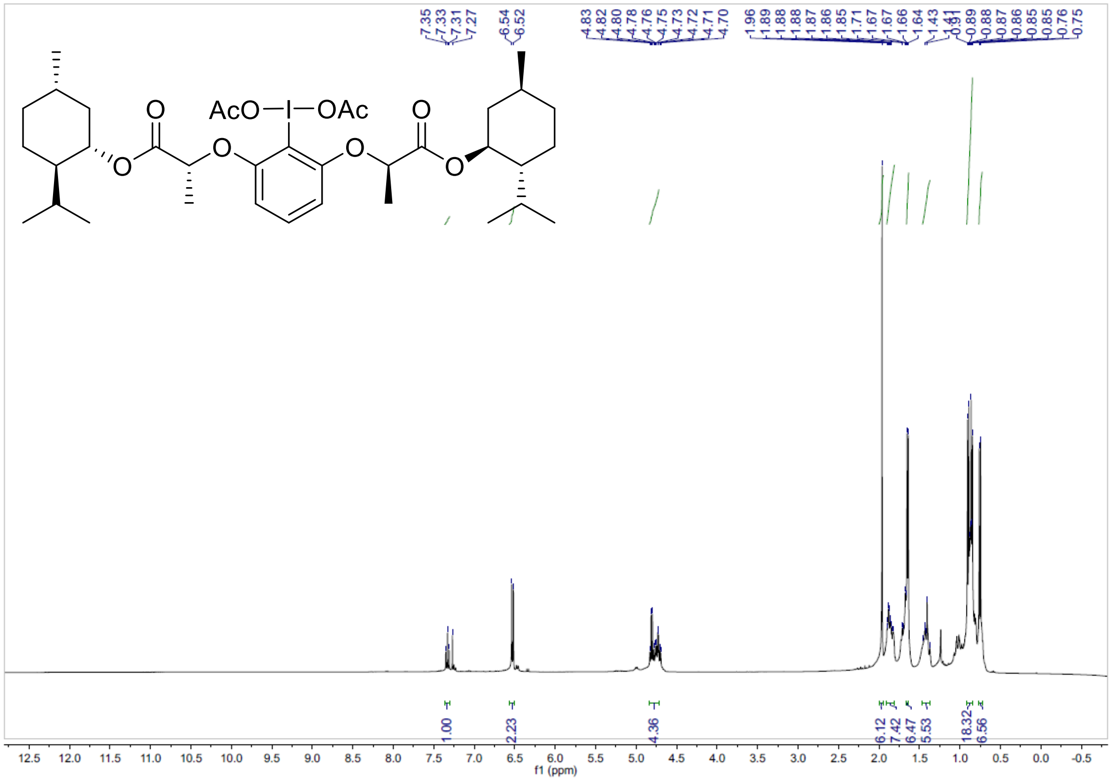

^{13}C NMR (101 MHz, CDCl$_3$)

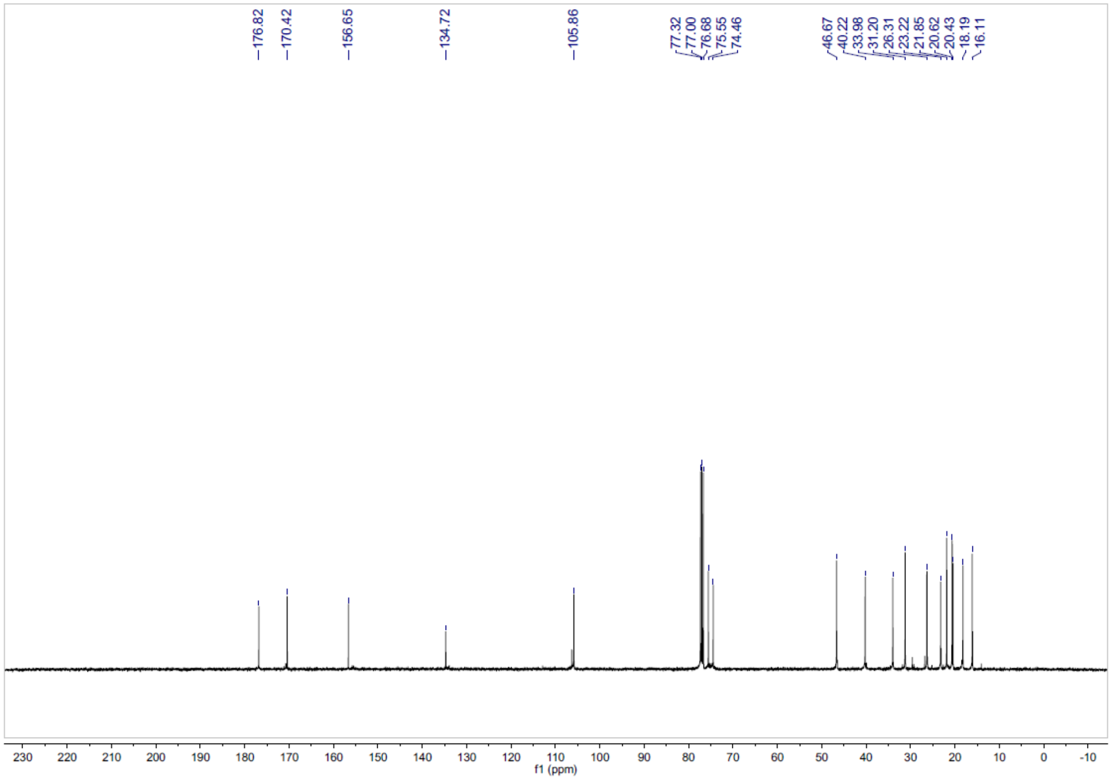

Bis((S)-1-phenylethyl) 2,2'-((2-(diacetoxy-l3-iodaneyl)-1,3-phenylene)bis(oxy))(2R,2'R)-dipropionate (33-OAc)

^{1}H NMR (500 MHz, CDCl$_3$)

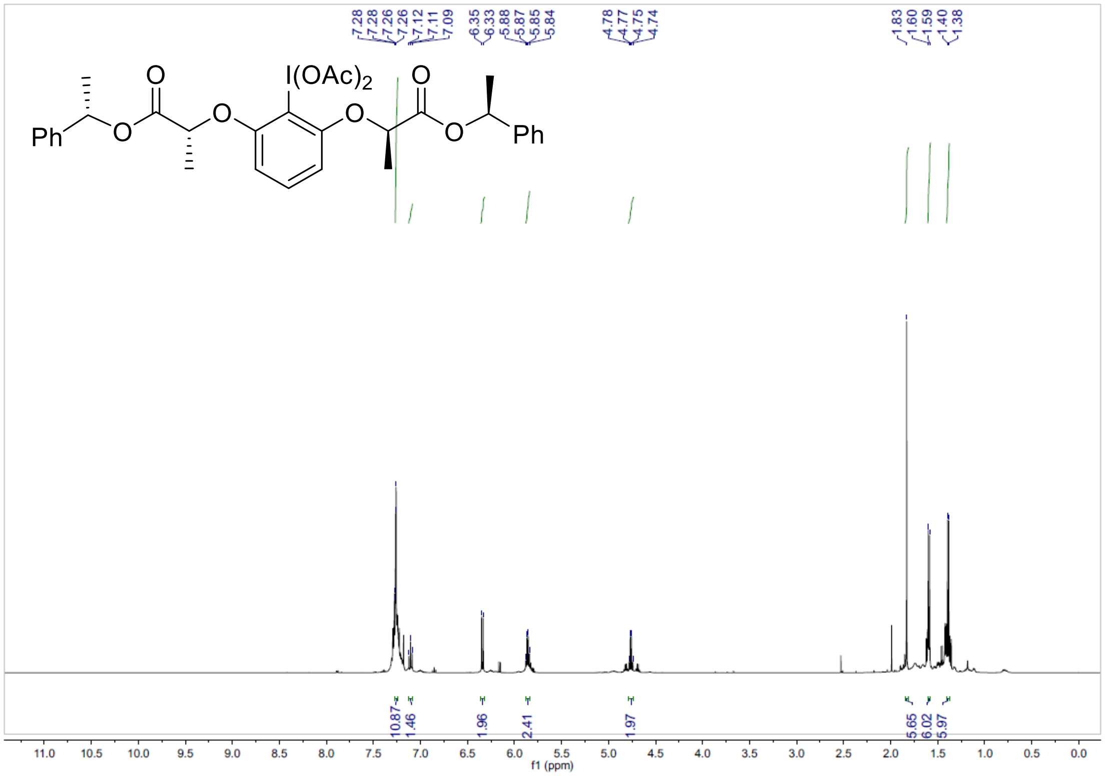

^{13}C NMR (126 MHz, CDCl$_3$)

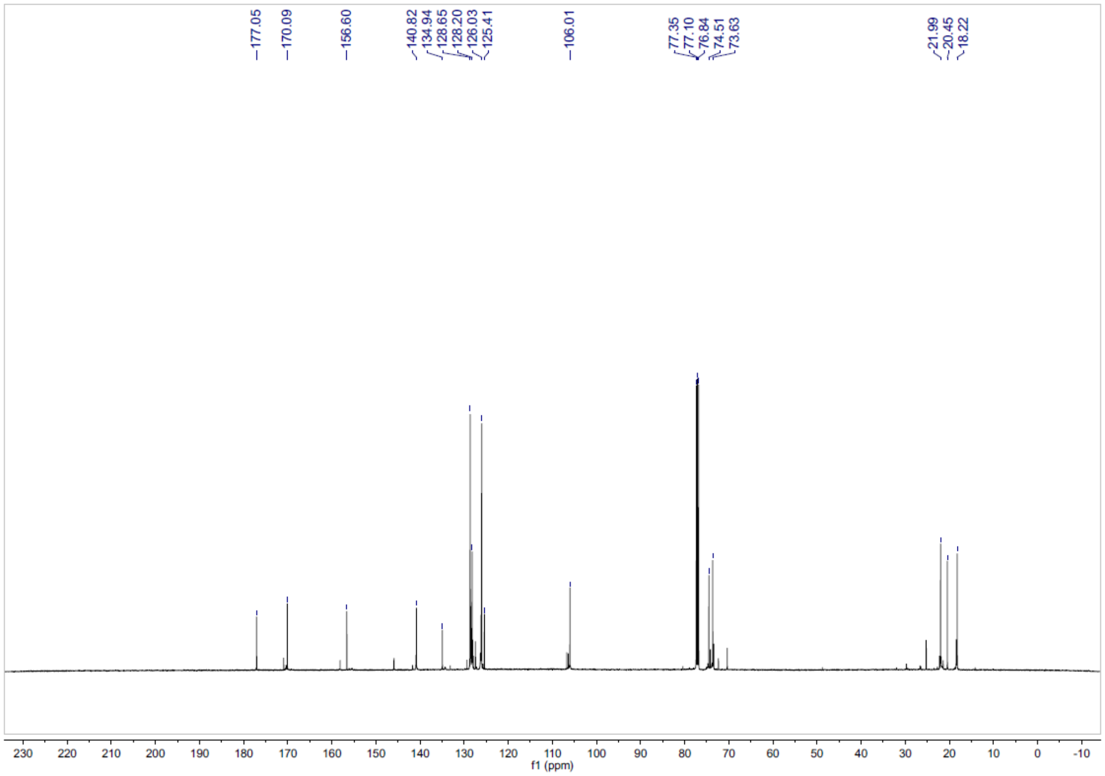

Dimesityl 2,2'-((2-(diacetoxy-l3-iodaneyl)-1,3-phenylene)bis(oxy))(2R,2'R)-bis(3-phenylpropanoate) (34-OAc)

^{1}H NMR (400 MHz, CDCl$_3$)

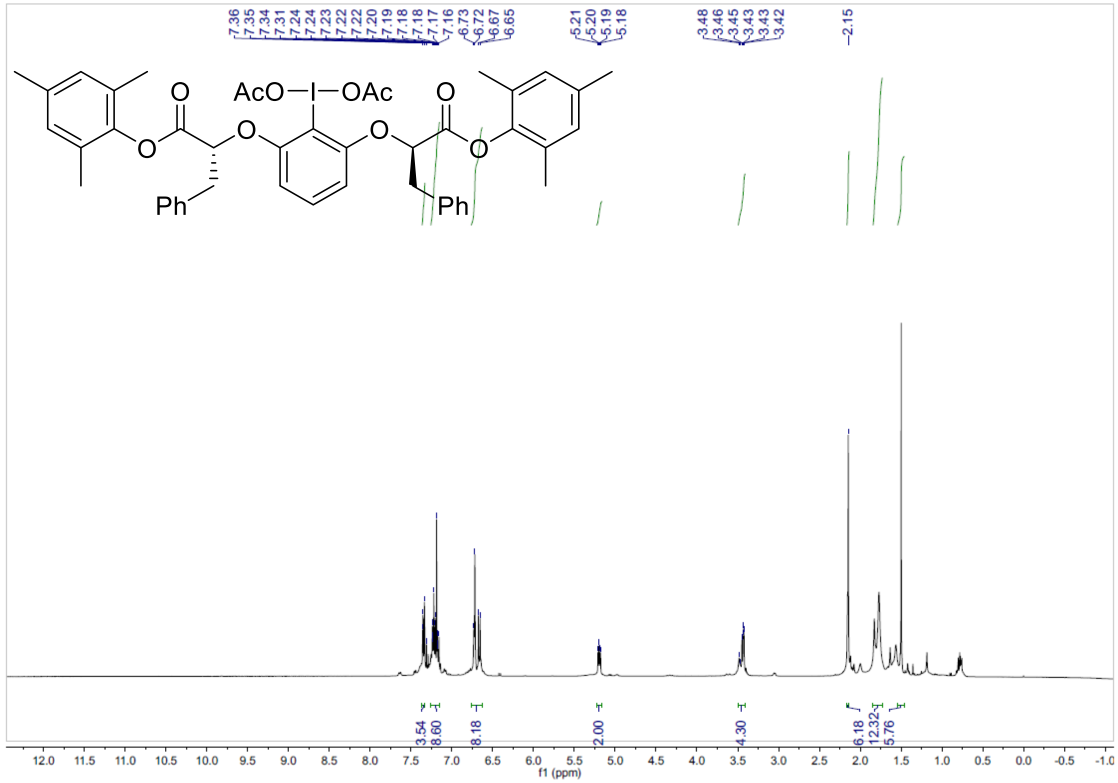

^{13}C NMR (101 MHz, CDCl$_3$)

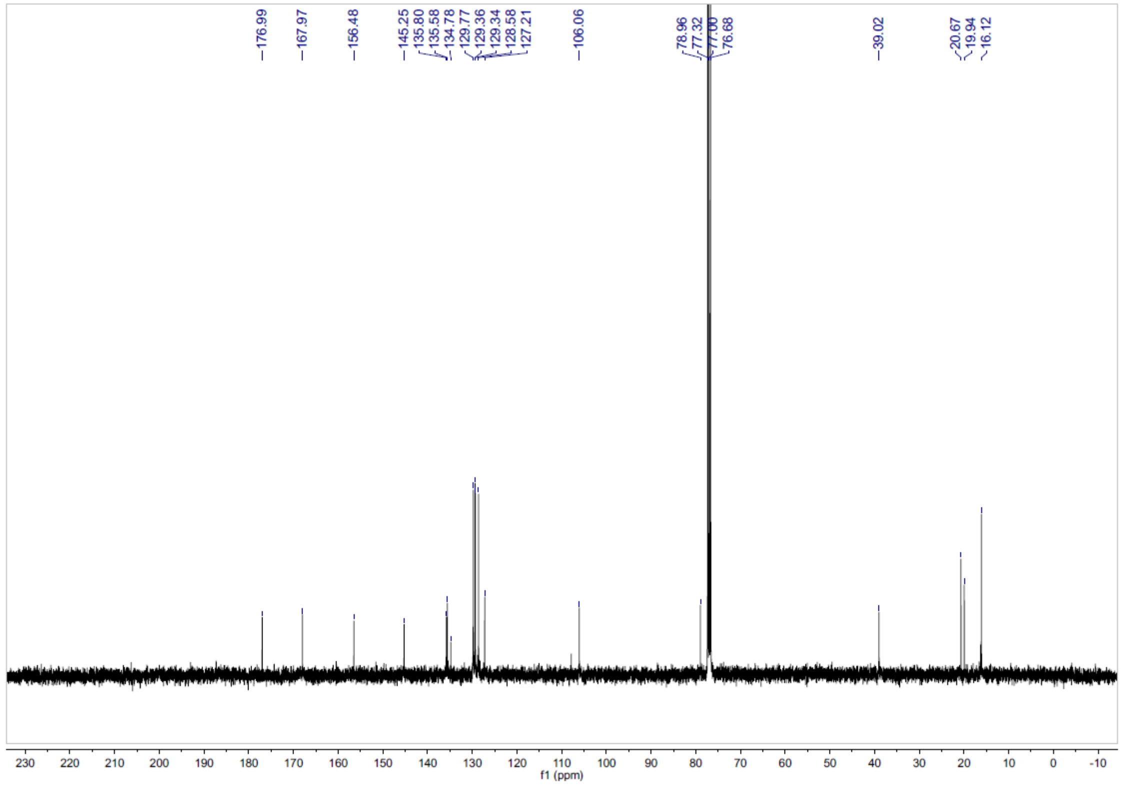

(2,6-bis(((S)-1-(diphenylamino)-1-oxopropan-2-yl)oxy)phenyl)-l3-iodanediyl diacetate (35-OAC)

^{1}H NMR (400 MHz, CDCl₃)

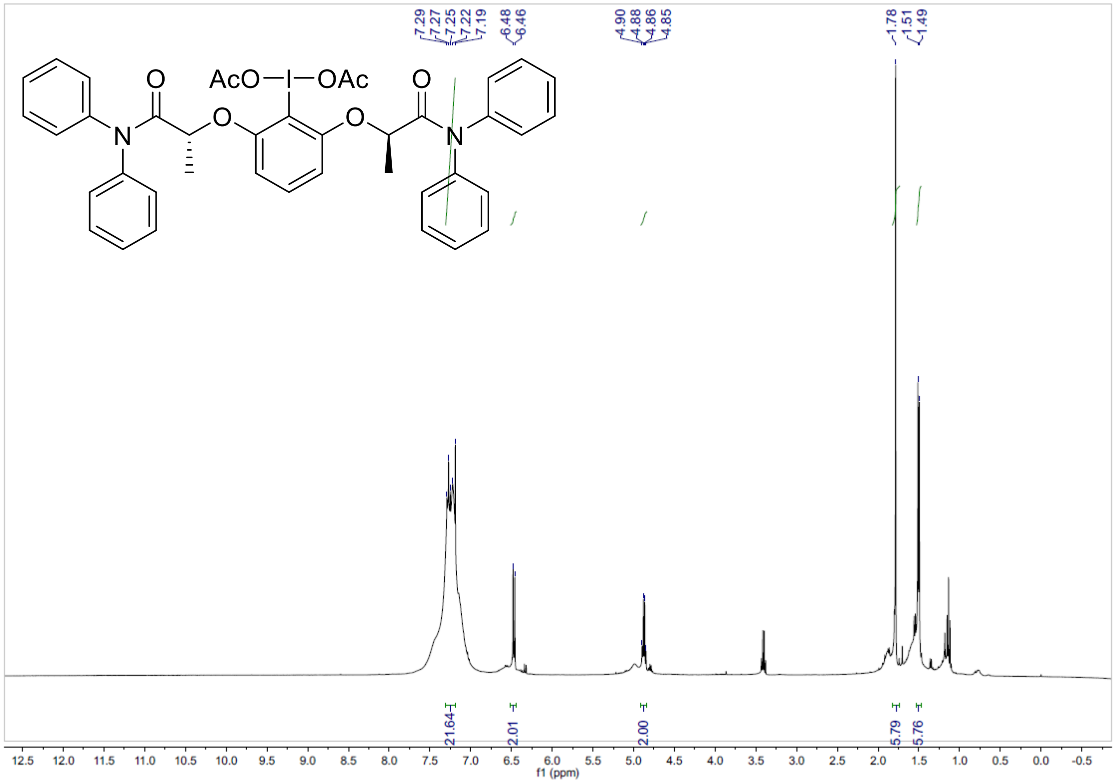

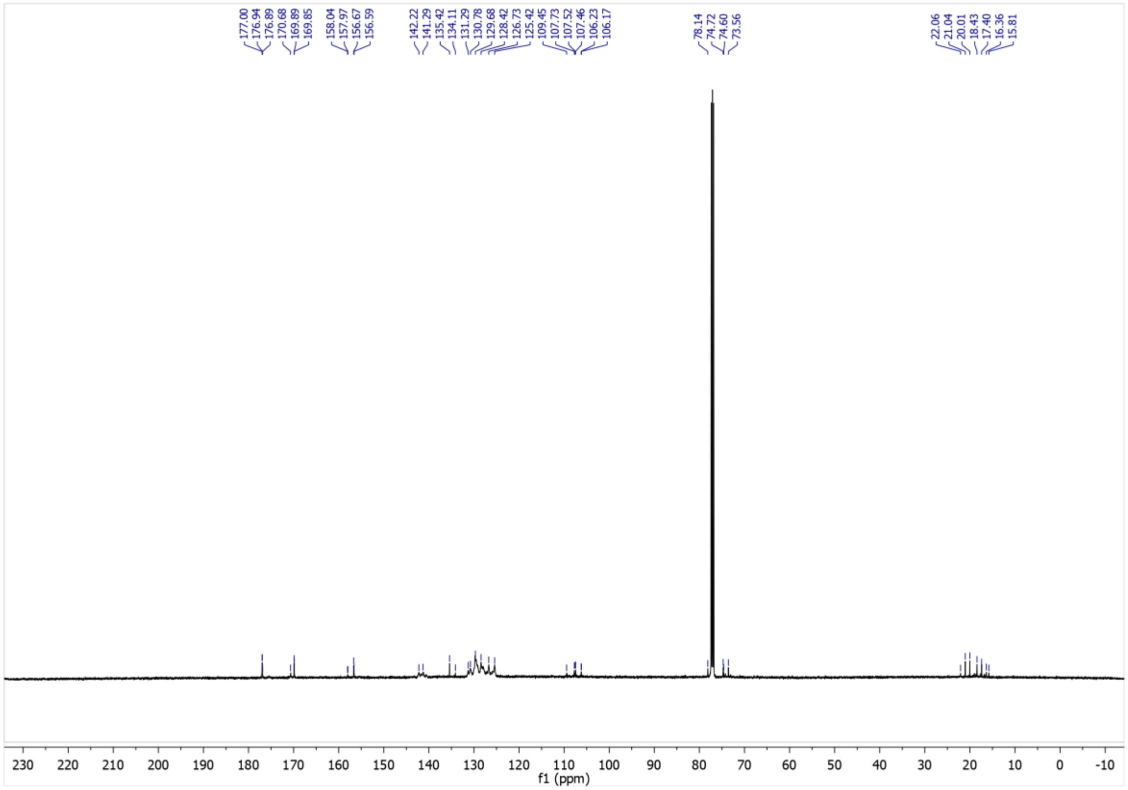

(2,6-bis(((R)-1-oxo-1-(((S)-1-phenylethyl)amino)propan-2-yl)oxy)phenyl)-l3-iodanediyl diacetate (36-OAC)

^{1}H NMR (500 MHz, CDCl3)

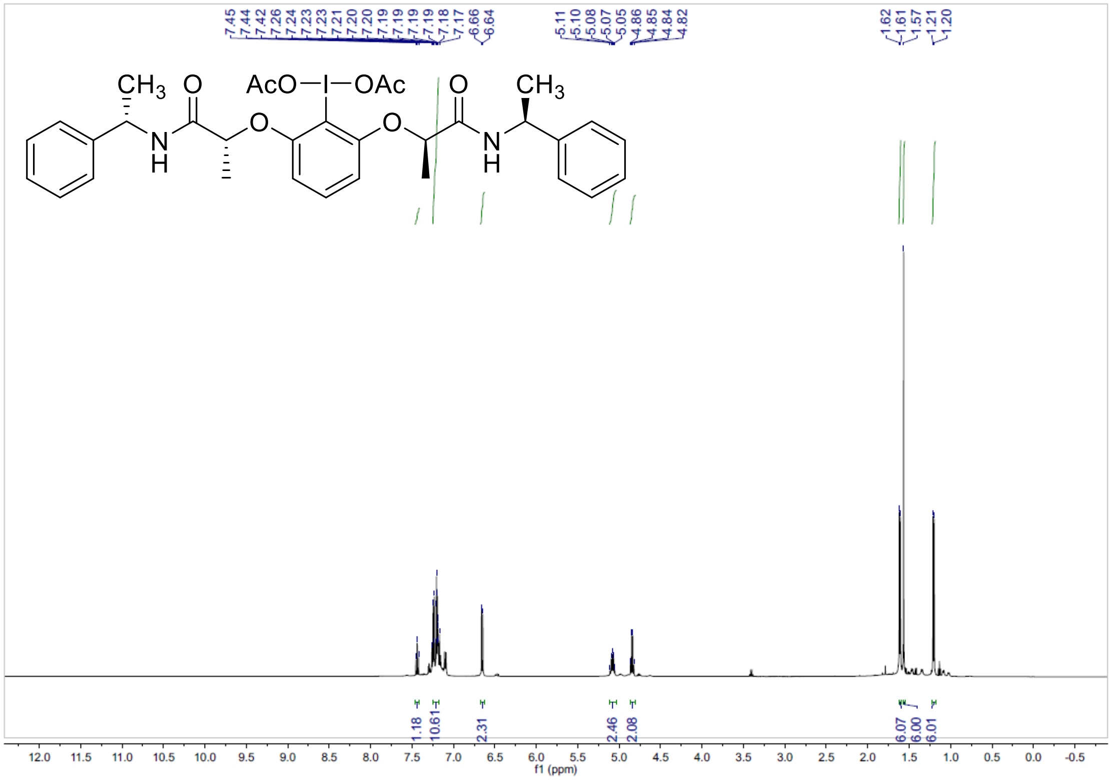

^{13}C NMR (126 MHz, CDCl3)

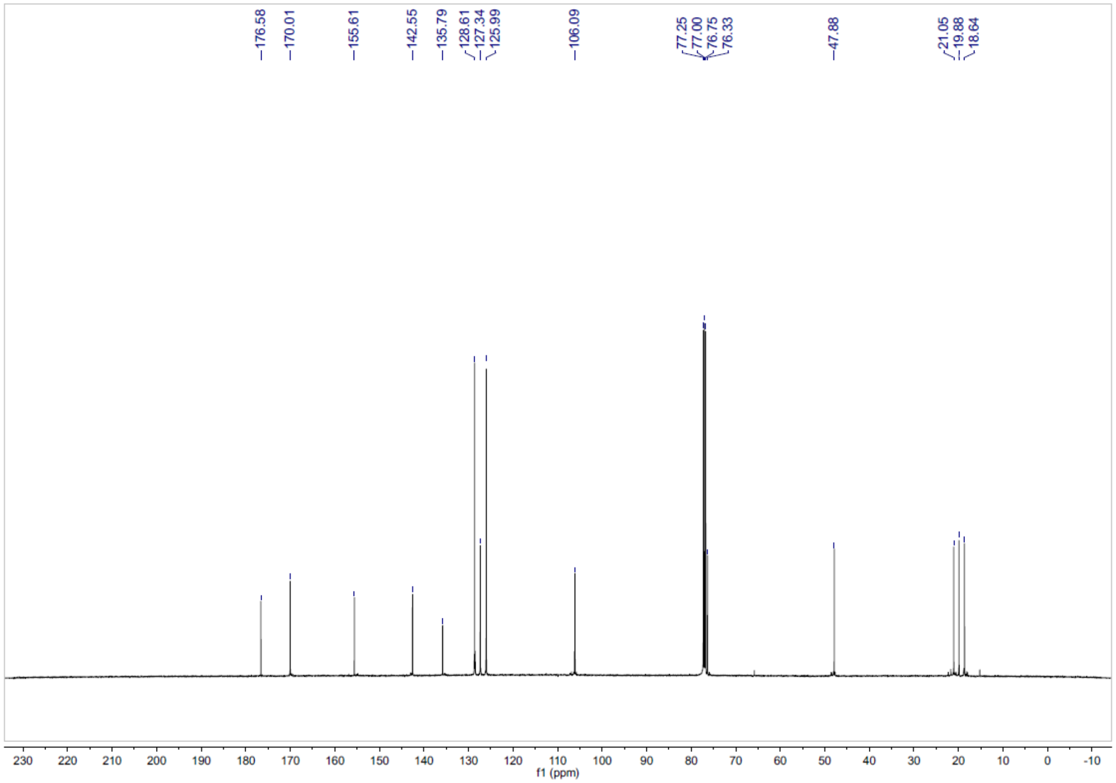

2. *Kinetic Analysis*

2.1 Standard kinetic procedure: Reaction sampling

566 mg (0.176 M, 1.1 equiv.) of PIDA was dissolved in 10 mL of acetonitrile by stirring at 27 °C in a capped, temperature controlled 25 mL vial. Once fully dissolved, 166 uL (0.16 M, 1 equiv.) of BnOH was added as well as 100 µL of 1,2-dichloroethane (internal standard). A 100 µL aliquot was then taken for ^{1}H analysis in 0.7 mL of $CDCl_3$. The kinetic experiments were initiated by adding 25 mg (0.016 M, 10 mol%) of TEMPO. 100 µL aliquots were taken over a 2-hour period. The aliquots were quenched using 0.5 mL of a 0.26 M aqueous sodium thiosulfate solution. The sample was extracted using 1 mL of $CDCl_3$ and passed through a plug of magnesium sulfate to dry. ^{1}H NMR spectra were analysed using Mestrenova 11 software, all signals integrated were subjected to linear correction, and all resonance were set relative to the proton signal of 1,2-dichloroethane (δ 3.6 ppm, s, 2H). All experiments were carried out in triplicate with an error of ca. 5%.

2.2 Kinetic procedure: In situ NMR

56.6 mg (0.176 M, 1.1 equiv.) of PIDA was dissolved in 1.0 mL of acetonitrile-d_3 by stirring at 27 °C in a capped, temperature controlled 25 mL vial. Once fully dissolved, 16.6 µL (0.16 M, 1 equiv.) of BnOH was added as well as 10.0 µL of 1,2-dichloroethane (internal standard). The sample was transferred to an NMR tube and an initial ^{1}H spectra was taken. Kinetic experiments were started by adding 2.5 mg (0.016 M, 10 mol%) of TEMPO and in-situ ^{1}H analysis was carried out over a 2-hour period. ^{1}H NMR spectra were analysed using Mestrenova 11 software, all signals integrated were subjected to linear correction, and all resonance were set relative to the proton signal of 1,2-dichloroethane (δ 3.6 ppm, s, 2H).

2.3 Standard reaction conditions for kinetic investigation

All reactions were conducted in a closed reaction vessel at 27 °C unless otherwise stated. Kinetic orders were investigated through change of concentration of one component at a time (Table S1A). No water was added to the reaction vessel unless otherwise stated. Changes were made to the original conditions of Piancatelli *et al.*[12] (Table S1B) to ensure a homogeneous reaction mixture that was amenable to sampling through removal of the low boiling point solvent.

Table S1: (A) Standard conditions used in this work to determine reaction orders and (B) Conditions used by Piancatelli *et al.*[12]

(A)

**Experiment**	**A**	**B**	**C**	**D**
**[BnOH] / M**	0.16	0.16	0.12	0.16
**[PIDA] / M**	0.176	0.16	0.176	0.176
**[TEMPO] / M**	0.016	0.016	0.016	0.032
**MeCN / mL**	10	10	10	10

(B)

**Experiment**	**A**
**[BnOH] / M**	1
**[PIDA] / M**	1.1
**[TEMPO] / M**	0.1
**CH_2Cl_2 / mL**	1

2.4 Kinetic Plots

Reaction orders were determined using the variable time normalization analysis (VTNA) procedure of Burés (Figure S1).[13]

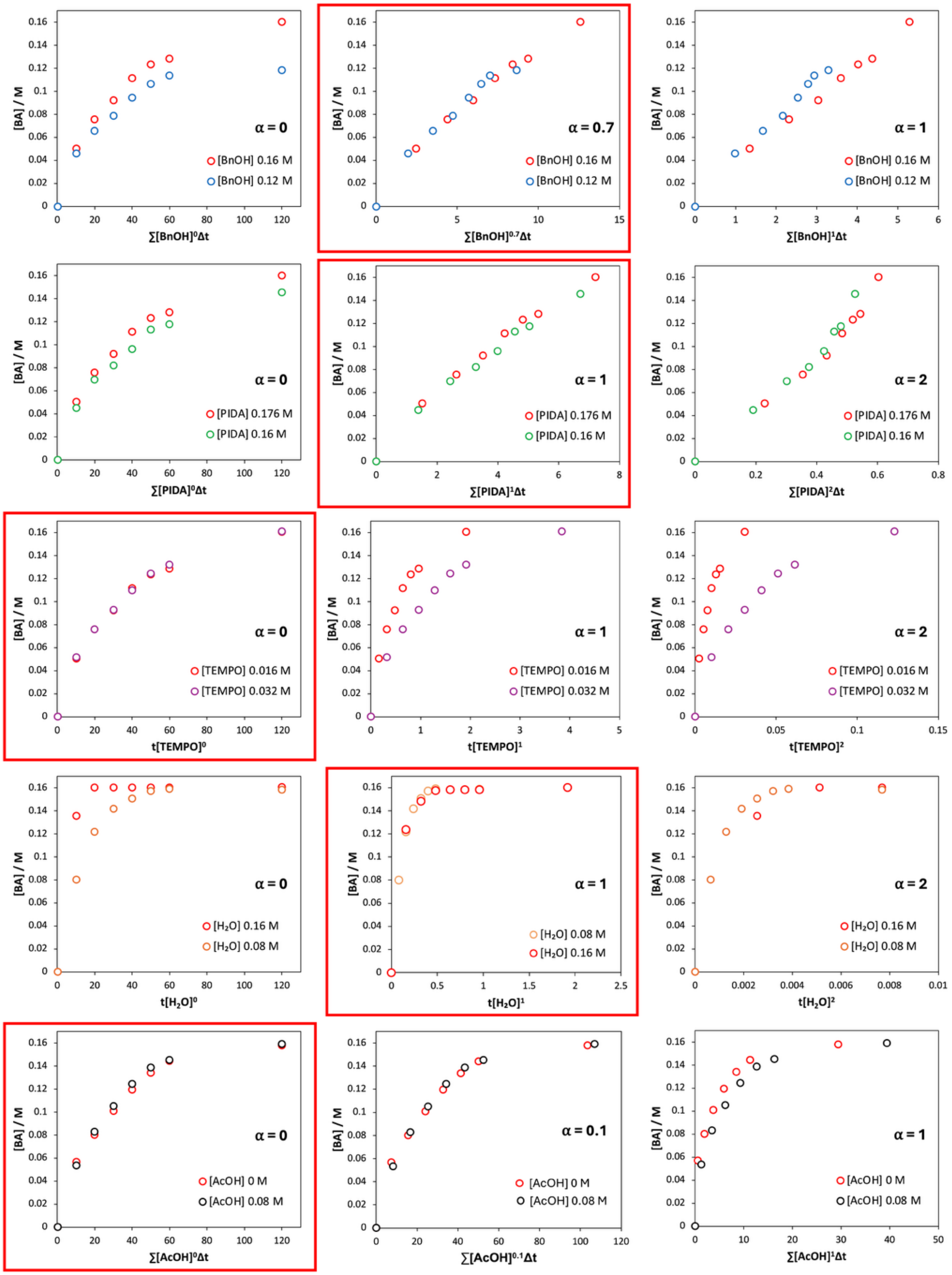

Figure S1: VTNA plots at different orders for each reagent showing overlay, or lack thereof. Red squares denote overlay. Standard conditions used throughout.

2.5 Varying [TEMPO]

A series of reactions were carried out where the concentration of TEMPO was varied (Figure S2). At significantly low loading of 0.1 mol%, where it might be expected to see a change in rate-determining step between dual catalytic cycles,[14] TEMPO undergoes deactivation, and the reaction does not reach completion.

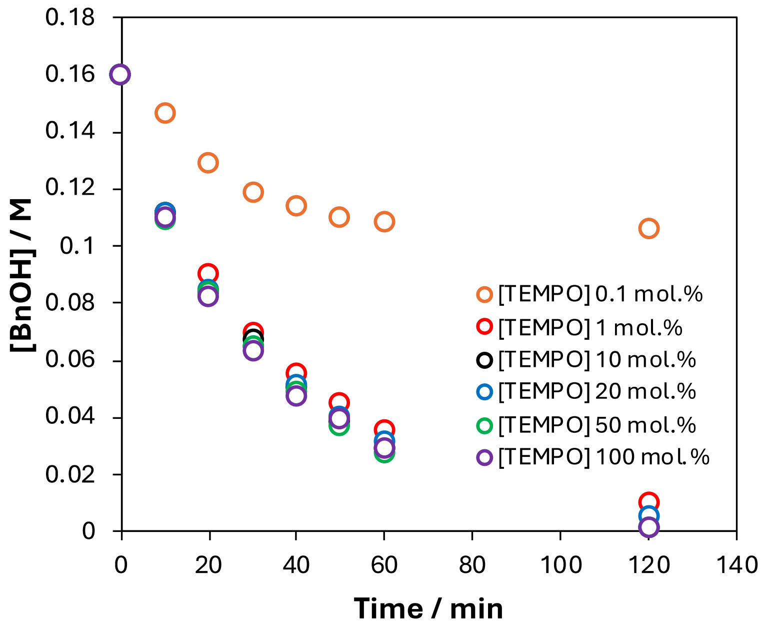

Figure S2: Reaction time-course data at varying [TEMPO], standard conditions.

3. Standard reaction under Schlenk conditions

When run under dry Schlenk conditions, the reaction is slower than when open to atmosphere (Figure S3A). To confirm this is due to the lack of water, rather than a lack of oxygen, the reaction was performed again under Schlenk conditions but with an atmosphere of oxygen, no change in the rate was observed (Figure S3B).

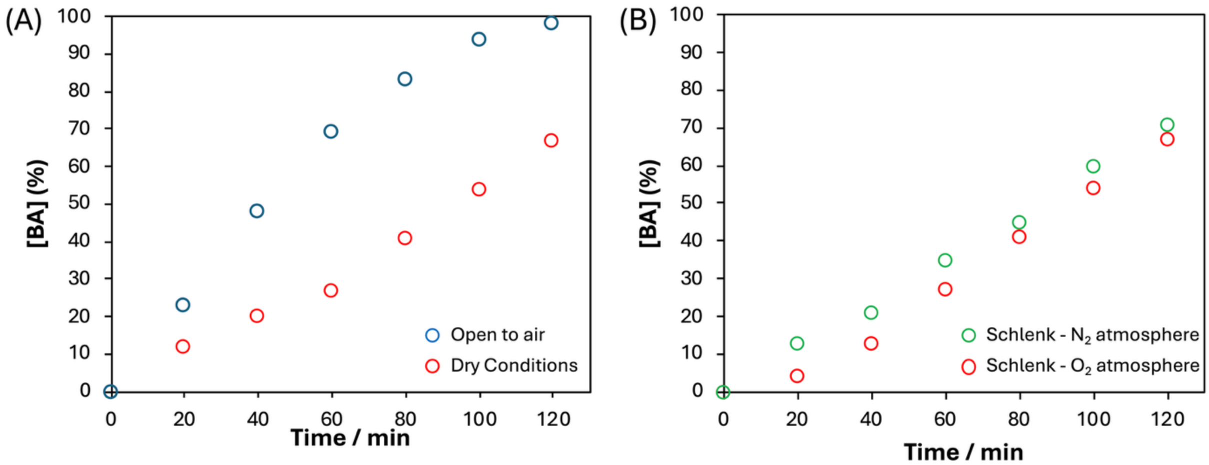

Figure S3: Yield of benzaldehyde vs time for: A) dry (Schlenk) conditions compared to open to air, standard (wet) conditions; B) reactions in inert (N_2) and O_2 atmosphere. Standard conditions used.

4. *Reactions with increasing [H_2O]*

It was observed that the reaction does not saturate in the presence of high concentrations of water (Figure S4).

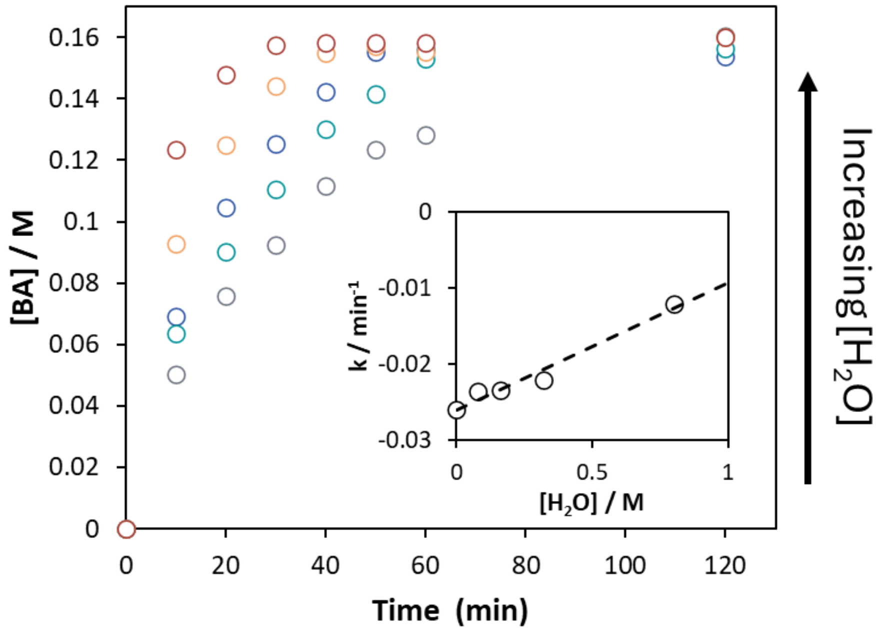

Figure S4: [Benzaldehyde] vs Time for increasing concentrations of H_2O (0, 50, 100, 200, 500 mol%). Standard conditions. Insert plot: A plot of rate constant *k* vs [H_2O] showing a straight line fit indicating first order kinetics in water.

That the reaction does not saturate with water rules out an off-cycle activator role for water in the context of the mechanism in Figure 8 or Scheme 5 in the main paper. The first order rate constant, *k*, was plotted against [H_2O] showing a good straight line fit indicating 1st order kinetics as demonstrated in Figure 1 and S1. It may be possible that PIDA sits off-cycle and requires activation to an on-cycle species (Scheme S1). PIDA is present in super-stoichiometric quantities (1.1 equiv.), while an activator, such as water or TEMPO would typically be present catalytic quantities. In the mechanism of Scheme S1, if [PIDA] > [Activator] then [Activator]^1. This rules out TEMPO as the activator but could fit with water as the activator. However, if the concentration of activator is increased so that [Activator] > [PIDA] then [Activator]^0. That the reaction remains first order in water at [H_2O] = 5 x [PIDA] rules out the off-cycle mechanism of Scheme S1.

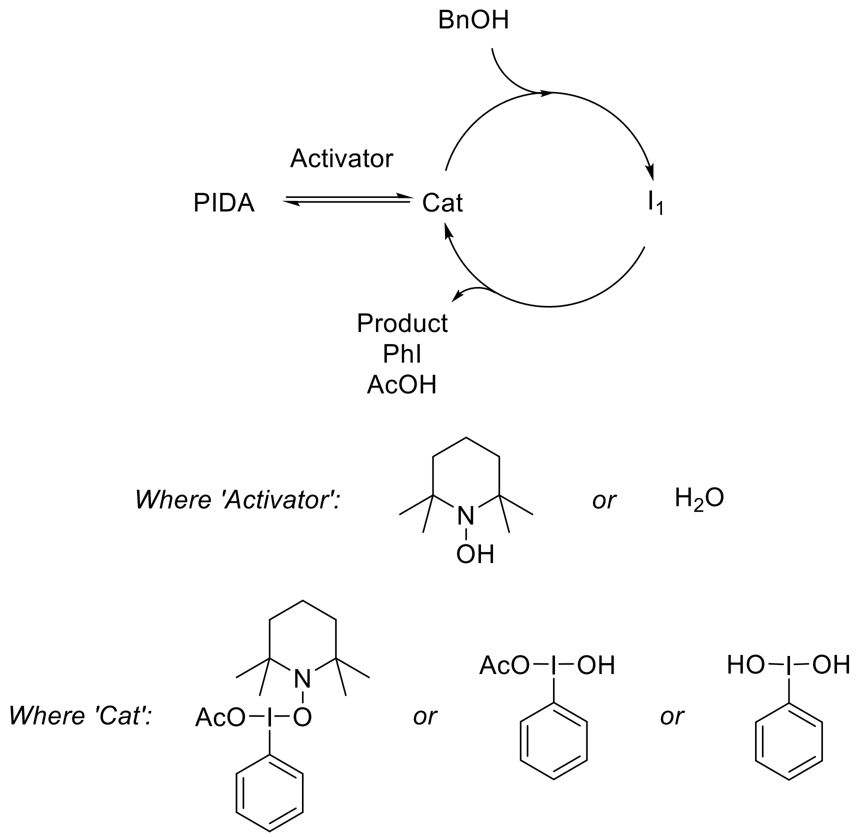

Scheme S1: Possible model mechanism involving a species which activates PIDA in an off-cycle step to an on-cycle active catalyst species.

The reaction was also carried out in the presence of 0.16 M $H_2{}^{18}O$. ^{18}O was not found in the reaction product, as determined by HRMS of the crude reaction mixture. Ruling out water as the oxygen source in the product aldehyde.

5. *Different alcohol VTNA*

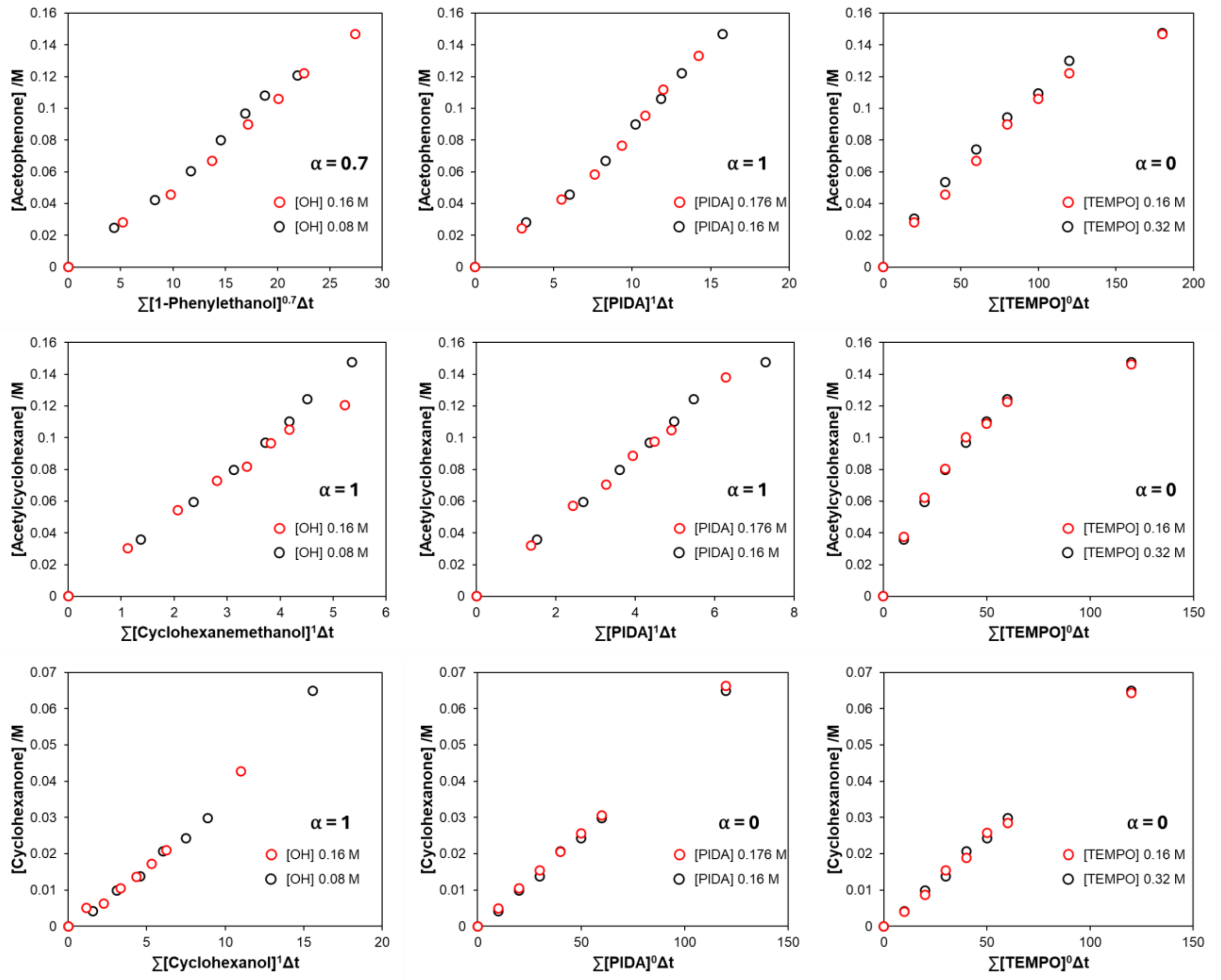

Figure S5: VTNA plots at different orders for each reagent for various alcohols corresponding to Figure 3 in the main body showing overlay.

6. ***Mono-alkoxy PIDA species***

Mixing PIDA and an alcohol results in the immediate formation of a mono-alkoxy iodine species which can be observed by ^{1}H NMR. This species can be monitored during the reaction using *in situ* ^{1}H NMR and, for benzyl alcohol, disappears within the first 20-30 minutes (Figure S6).

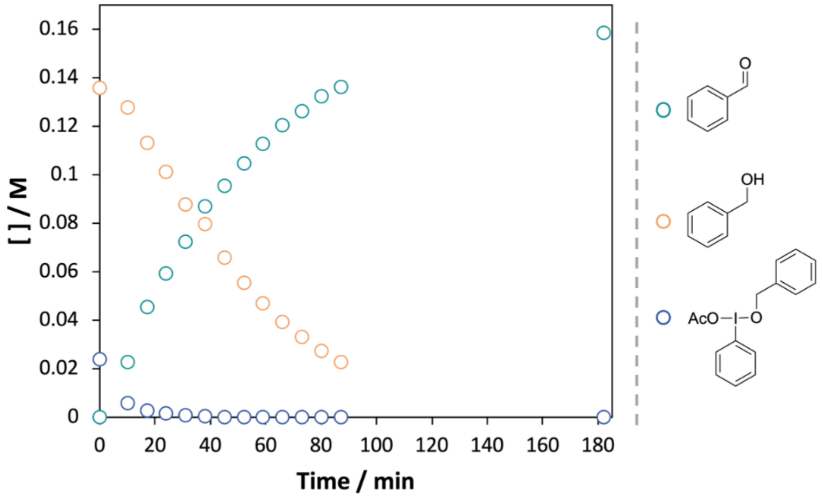

Figure S6: Kinetic profiles showing the concentration of benzaldehyde, benzyl alcohol and a mono-alkoxy PIDA species over time as determined using *in situ* ^{1}H NMR.

The mono-alkoxy species is present in different concentrations for different alcohols (Figure S7). We note that this species is present in higher concentrations for benzyl alcohol and 1-phenyl ethanol, both displaying 0.7 order in alcohol, than for cyclohexane methanol, which displays first order kinetics in alcohol. Although qualitative, these observations fit with the possibility of different experimental turnover determining intermediates expressed by our rate law.

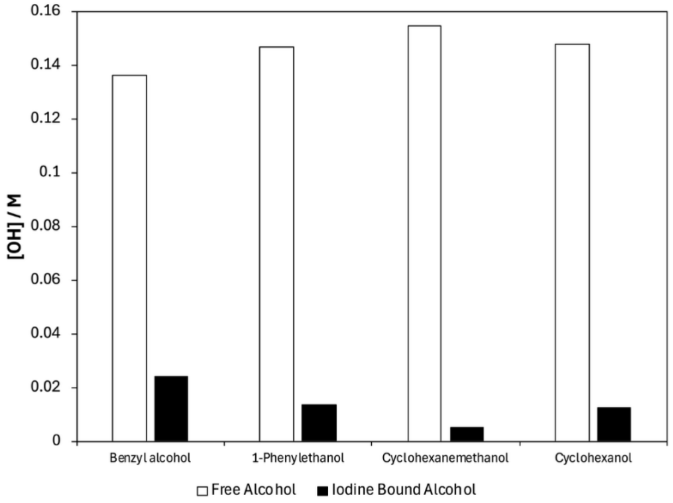

Figure S7: Relative abundance of free and iodine bound alcohol for mixtures of PIDA and different alcohols. [Alcohol] = 0.16M, [PIDA] = 0.176M

7. Stoichiometric acetic acid formation

At the end of the reaction, two equivalents of acetic acid are formed (Figure S8). It may be expected that a radical pathway involving an acetoxy radical would result in the decomposition of the acetoxy radical, this is not observed.

(top) Benzaldehyde, δH (600 MHz, 25.0 °C, $CDCl_3$): 10.04 (1H, s, CHO)

(bottom) Acetic acid, δH (600 MHz, 25.0 °C, $CDCl_3$): 2.01 (3H, s, CH_3)

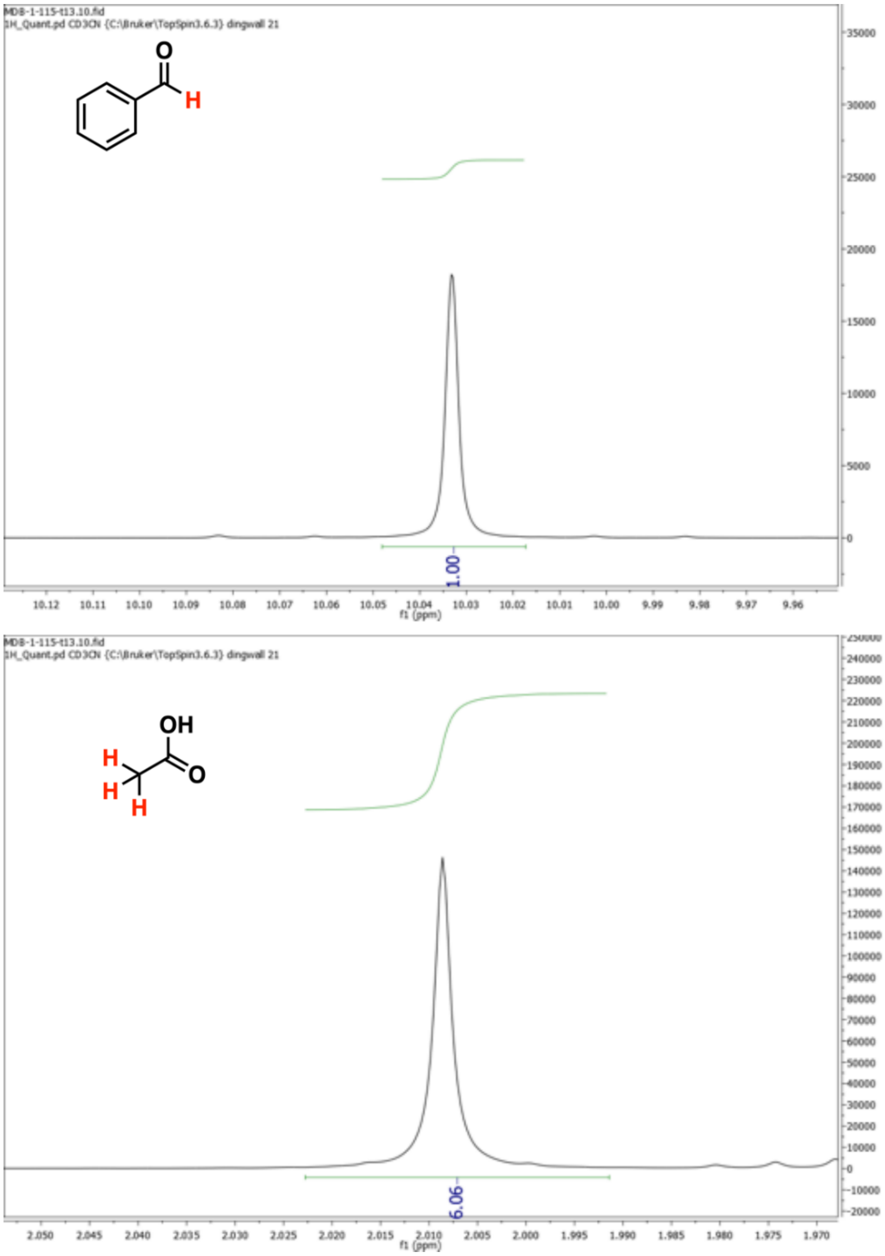

Figure S8: ^{1}H NMR data showing two equivalents of acetic acid forming for every one equivalent of benzaldehyde formed.

8. *Ligand tethered hypervalent iodine species*

Hypervalent iodine species, such as PIDA, have the potential to undergo two ligand exchanges. 3,3-dimethyl-1λ³-benzo[d][1,2]iodaoxol-1(3*H*)-yl acetate (DMB), was synthesized as a hypervalent iodine compounds with only one acetate ligand available for ligand exchange. The standard reaction was carried out with DMB in place of PIDA. DMB is poorly soluble in acetonitrile, and the reaction was conducted at 50 °C to ensure a homogeneous reaction mixture. A comparative reaction with PIDA was also conducted at 50 °C (Figure S9). The reaction does not proceed with DMB, highlighting the requirement for two labile ligands on the hypervalent iodine moiety.

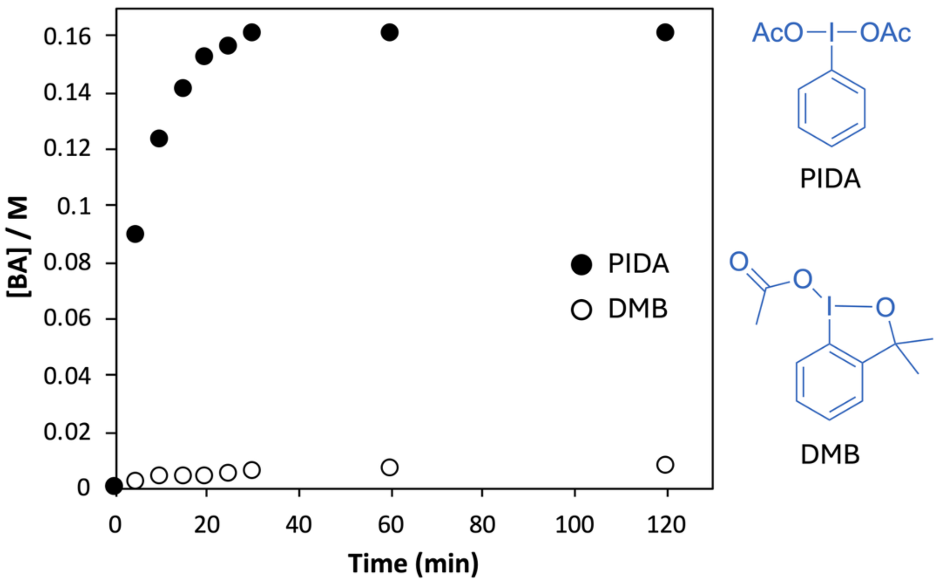

Figure S9: The comparative formation of benzaldehyde for PIDA and DMB at 50 °C, monitored by ^{1}H NMR. Standard conditions.

9. Derivation of rate equation

A summary of on- and off-cycle steps as shown in Scheme 5 in the main text. A rate equation can be derived using the quasi-equilibria assumption. CAT is used in place of PIDA.

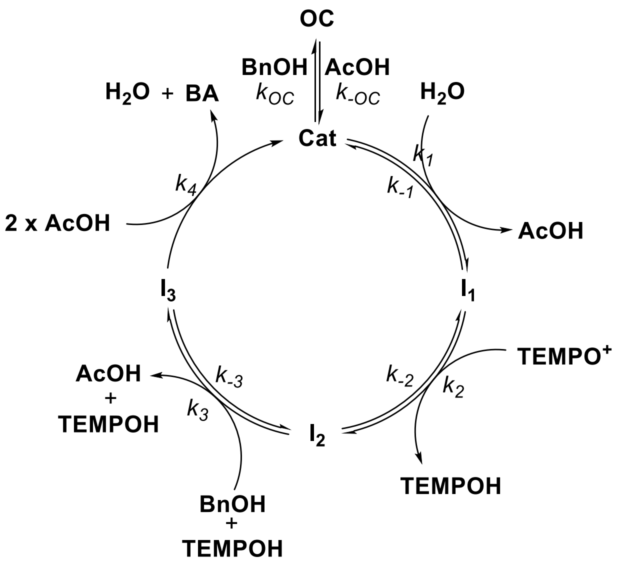

On-Cycle

(1) CAT + H_2O ⇌ I_1 + AcOH (k_1 and k_{-1})

(2) I_1 + $TEMPO^+$ ⇌ I_2 + TEMPOH (k_2 and k_{-2})

(3) I_2 + BnOH + TEMPOH ⇌ I_3 + AcOH + TEMPOH (k_3 and k_{-3})

(4) I_3 + 2AcOH → I_4 + BA + H_2O (k_4)

Off-Cycle

5) CAT + BnOH ⇌ OC + AcOH (k_{OC} and k_{-OC})

Forward reactions

$$r_1 = k_1[CAT][H_2O]$$

$$r_2 = k_2[I_1][TEMPO^+]$$

$$r_3 = k_3[I_2][BnOH][TEMPOH]$$

$$r_4 = k_4[I_3][AcOH]^2$$

$$r_{OC} = k_{OC}[CAT][BnOH]$$

Reverse reactions

$$r_{-1} = k_{-1}[I_1][AcOH]$$

$$r_{-2} = k_{-2}[I_2][TEMPOH]$$

$$r_{-3} = k_{-3}[I_3][AcOH][TEMPOH]$$

$$r_{-OC} = k_{-OC}[OC][AcOH]$$

Intermediate 1 (I_1)

$$r_1 = r_{-1}$$

$$k_1[CAT][H_2O] = k_{-1}[I_1][AcOH]$$

$$[I_1] = K_1 \frac{[H_2O][CAT]}{[AcOH]}$$

Intermediate 2 (I_2)

$$r_2 = r_{-2}$$

$$k_2[I_1][TEMPO+] = k_{-2}[I_2][TEMPOH]$$

$$[I_2] = K_2 \frac{[TEMPO^+]}{[TEMPOH]}[I_1]$$

$$[I_2] = K_1K_2 \frac{[H_2O][CAT]}{[AcOH]} \frac{[TEMPO^+]}{[TEMPOH]}$$

Intermediate 3 (I_3)

$$r_3 = r_{-3}$$

$$k_3[I_2][BnOH][TEMPOH] = k_{-3}[I_3][AcOH][TEMPOH]$$

$$k_3[I_2][BnOH] = k_{-3}[I_3][AcOH]$$

$$[I_3] = K_3 \frac{[BnOH]}{[AcOH]}[I_2]$$

$$[I_3] = K_1K_2K_3 \frac{[BnOH][H_2O][CAT]}{[AcOH]^2} \frac{[TEMPO^+]}{[TEMPOH]}$$

Off-Cycle Intermediate OC

$$r_{OC} = r_{-OC}$$

$$k_{OC}[CAT][BnOH] = k_{-OC}[OC][AcOH]$$

$$[OC] = K_{OC} \frac{[CAT][BnOH]}{[AcOH]}$$

Catalyst Mass Balance

Where Cat = PIDA. Assume that PIDA exists solely as free PIDA, Cat, and off-cycle mono-alkoxy complex, OC (see Figure S6).

$$[CAT]_T = [CAT] + [OC]$$

$$[CAT] = \frac{[CAT]_T}{1 + K_{OC}\frac{[BnOH]}{[AcOH]}}$$

Rate of product formation

$$r_4 = k_4[I_3][AcOH]^2$$

$$r_4 = k_4K_1K_2K_3\frac{[BnOH][H_2O][CAT]}{[AcOH]^2}\frac{[TEMPO^+]}{[TEMPOH]}[AcOH]^2$$

$$r_4 = k_4K_1K_2K_3[BnOH][H_2O][CAT]\frac{[TEMPO^+]}{[TEMPOH]}$$

$$r_4 = k_4K_1K_2K_3\frac{[BnOH][H_2O][CAT]_T}{1 + K_{OC}\frac{[BnOH]}{[AcOH]}}\frac{[TEMPO^+]}{[TEMPOH]}$$

Assuming [$TEMPO^+$] = [TEMPOH], these terms cancel out to leave the final expression.

$$rate = k_4K_1K_2K_3\frac{[BnOH][H_2O][CAT]_T}{1 + K_{OC}\frac{[BnOH]}{[AcOH]}}$$

Depending on the dominant turnover determining intermediate, the rate equation may simplify two ways. In situ NMR studies (Figure S6) show that speciation of PIDA changes over the course of the reaction, with *OC* present only for the first ca. 20 minutes, 17% conversion, of the reaction. In this regime with two turnover determining intermediates present, the rate equation remains the same as above, with a BnOH term in the numerator and denomination resulting in partial order overall in BnOH. AcOH should be positive order, our data suggests the order in AcOH is between 0 and 0.1 (Figure S1), with such a small order difficult to separate from experimental noise. After ca. 20 minutes, catalyst speciation has moved solely to PIDA, simplifying the rate equation, and removing any dependence on AcOH:

$$rate = k_4K_1K_2K_3[BnOH][H_2O][CAT]_T$$

10. Oxidative Kinetic Resolution Optimisation

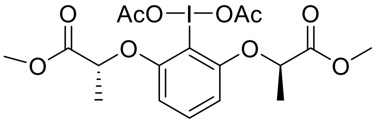

27-OAc

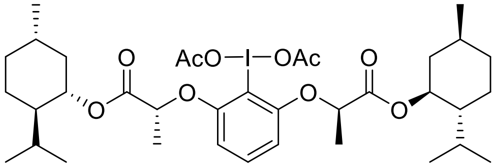

32-OAc

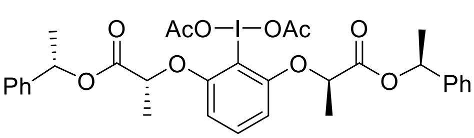

33-OAc

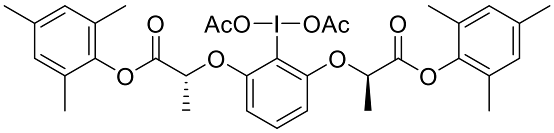

31-OAc

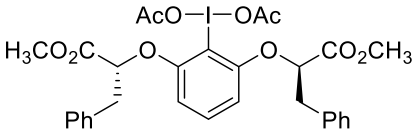

28-OAc

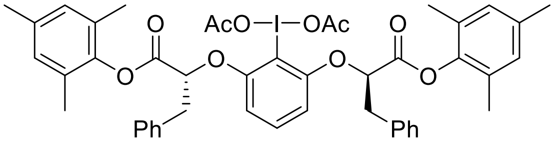

34-OAc

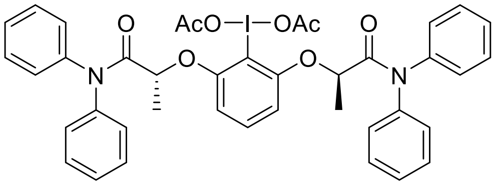

35-OAc

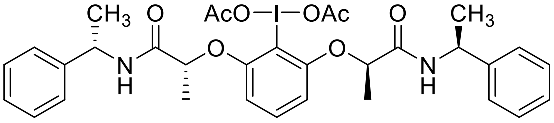

36-OAc

Figure S10: Synthesized chiral hypervalent iodine reagents.

Table S2: Optimisation of Oxidative kinetic resolution of 1-indanol

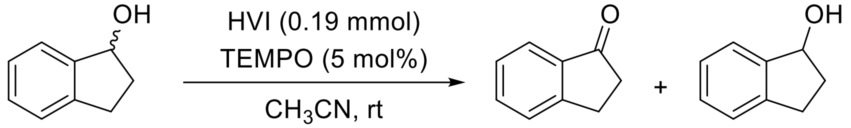

***rac*-1-indanol** (0.37 mmol) **1-indanone** **1-indanol**

Entry	effect of HVI	yield of **indanone**	recovered SM	*ee (%)*	s-factor
1	27-OAc	61%	36%	*rac*	-
2	32-OAc	38%	48%	*rac*	-
3	33-OAc	45%	44%	*13%*	1.6
4	31-OAc	46%	48%	15%	1.6
5	28-OAc	46%	44%	*rac*	-
**6**	**34-OAc**	**43%**	**45%**	**17%**	**1.8**
7	35-OAc	40%	45%	14%	1.7
8	36-OAc	46%	43%	*rac*	-

HPLC Traces

Entry 4, 31-OAc

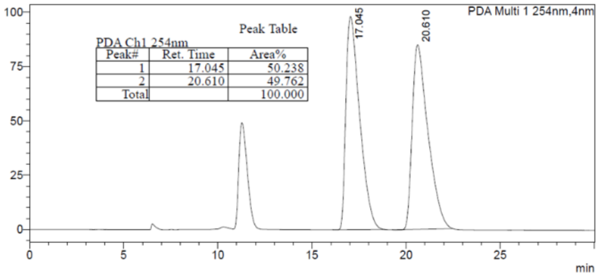

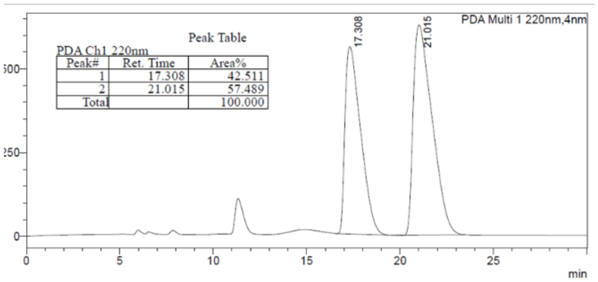

Entry 3, 33-OAc

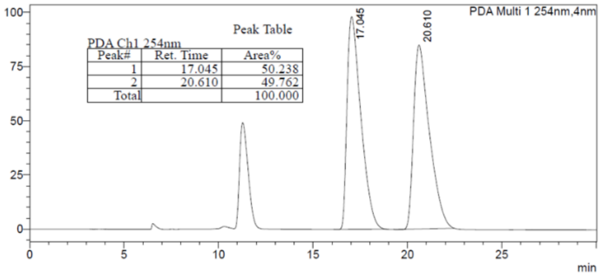

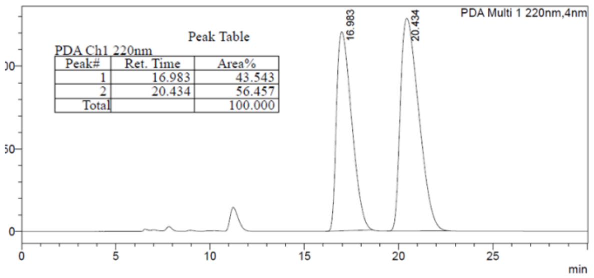

Entry 6, 34-OAc

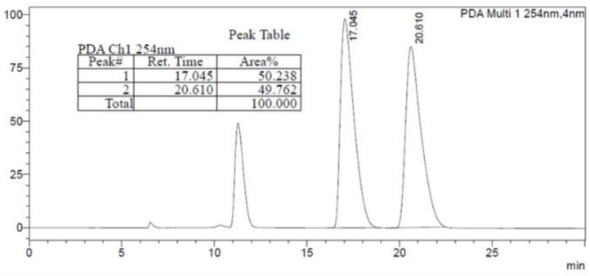

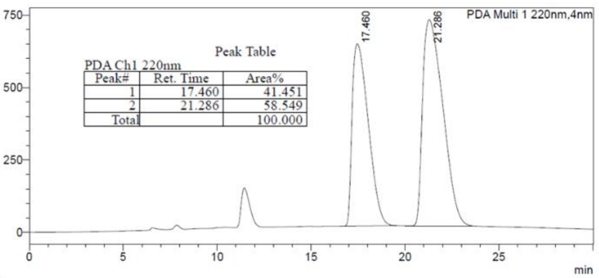

Entry 7, 35-OAc

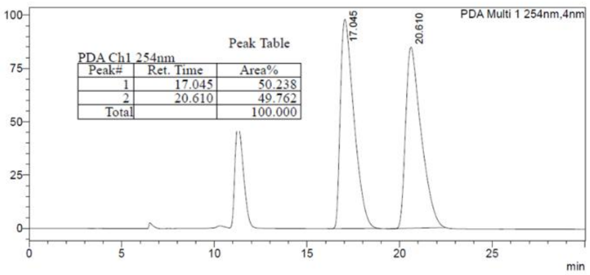

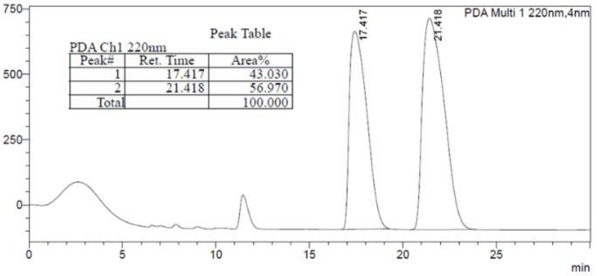

11. Density Functional Theory (DFT) calculations

11.1 Computational Details

All optimization, frequency, and single point calculations were performed with Gaussian 16, rev C.01. The M06-2X[15-16] functional was used for all geometry optimizations with the 6-31G(d,p) basis set on all non-metal atoms and the SDD[17] valence basis set and pseudopotential to describe I. Transition states on the potential energy surface were located using scans and a coordinate driving methodology. All optimized structures were confirmed as either minima or saddle points by the presence of zero or one imaginary harmonic frequency respectively. All transition states were further analyzed via intrinsic reaction coordinate (IRC) calculations to ensure intermediates connected to their corresponding transition state structures. On the basis of the optimized structures, single point refinements were made with the Def2TZVP[18] basis set for all atoms. Corrections for bulk solvation were included through a polarizable continuum model (PCM)[19] approach (acetonitrile). Free energies were determined from thermochemical corrections of the geometries applied to electronic energies. Considerable care was taken to identify low energy conformers of intermediates and transition states through extensive conformational sampling. All structures reported are the most energetically stable of those sampled. Non-covalent interaction (NCI) surfaces were calculated using promolecular densities in nciplot.[20] Structures with surfaces are displayed with PyMOL, rendered with the excellent Paton group display settings.[21] Other three dimensional structures are rendered with CYLview.[22]

11.2 Comparison of PIDA Ligand Exchange Pathways

Ligand exchange on hypervalent iodine at an acetate is accepted to occur by two possible pathways (Scheme S2).[23-24]

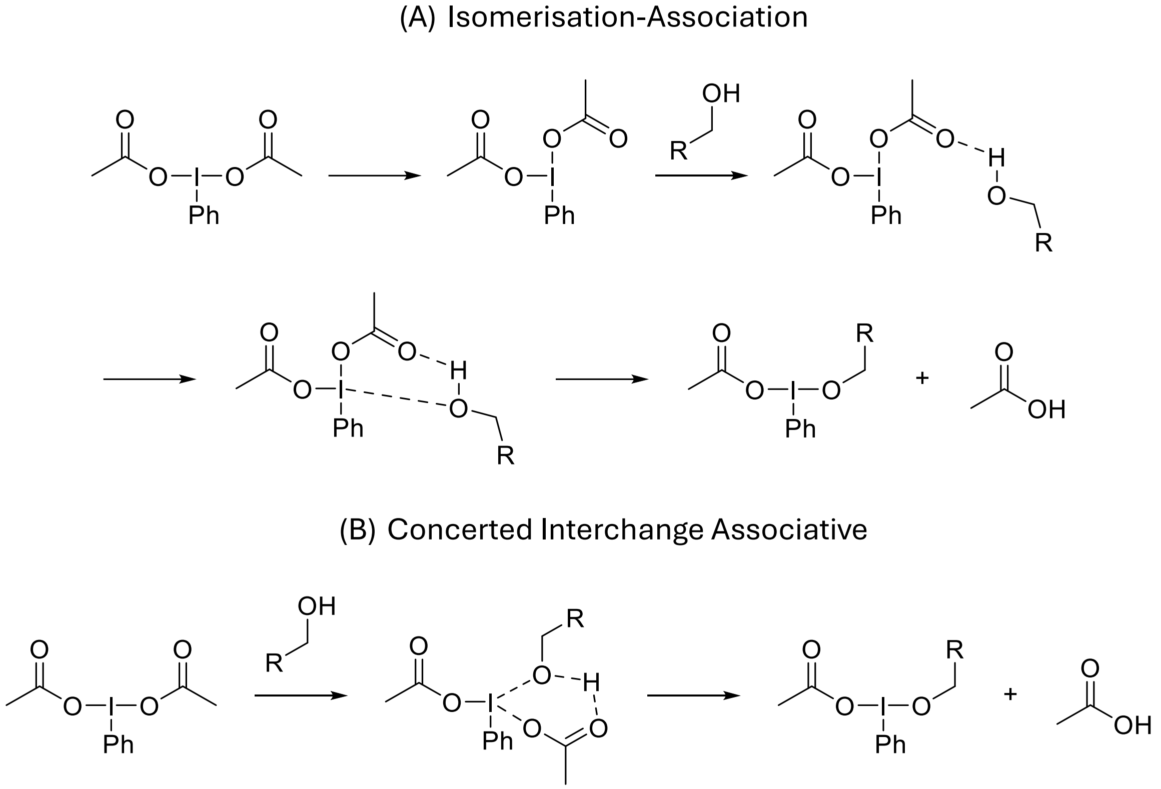

Scheme S2: Microscopic mechanisms for ligand exchange on hypervalent iodine at acetate: A) Isomerisation-association pathway; B) Concerted-interchange-associative pathway

PIDA can exchange an acetate ligand with any molecule bearing an O-H moiety. In the reaction under study, this includes benzyl alcohol, water, and hydroxylamine. Each potential exchange could form a new iodine(III) species which can in turn undergo a further ligand exchange at the remaining acetate, leading to significant potential complications. In all but a single case, exchange of benzyl alcohol on PIDA, isomerisation then association is more energetically favoured than the concerted pathway (Table S2). Further, isomerization is always higher in energy than association. As noted in the main body, we could not find hydrogen bond transition states which must come prior to association but estimated this to be at least isoenergetic to isomerization.[23]

Table S3: Comparison of the concerted interchange association pathway against isomerization and association for different substrates starting from different possible I(III) compounds. Energies are all relative to PIDA and are Gibbs free energies (kcal mol^{-1}) computed at M06-2X/Def2TZVP/PCM$_{acetonitrile}$//M06-2X/6-31G(d,p)/SDD(I).

Starting Material	Exchanging Ligand	Concerted Interchange Associative (kcal mol^{-1})	Isomerization (kcal mol^{-1})	Association (kcal mol^{-1})
	Benzyl alcohol	23.8		19.5
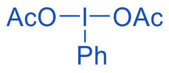 (0.0)	H_2O	24.8	24.1	19.8
	Hydroxylamine	25.4		20.3
	Benzyl alcohol	22.7		15.3
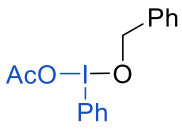 (2.0)	H_2O	20.4	18.5	N/A
	Hydroxylamine	22.7		15.2
	Benzyl alcohol	23.6		19.2
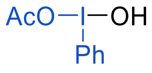 (3.5)	H_2O	24.7	21.2	18.6
	Hydroxylamine	24.8		19.4
	Benzyl alcohol	19.8		16.9
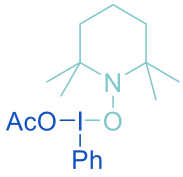 (-0.8)	H_2O	19.7	N/A	N/A
	Hydroxylamine	22.1		N/A

11.3 Simplified Potential Energy Surface

Figure S11 shows the potential energy surface of Figure 10 highlighting key transition states and intermediates, all minor structures not important to the kinetics or mechanistic arguments have been greyed out. Important energetic spans which determine the computational predicted turnover determining steps are highlighted in red. Key structures are described in Table S4.

Table S4: Key transition states and intermediates outlined in Figure S11

Structure	Role
**1**	PIDA, resting state, turnover determining intermediate
**TS1**	Isomerisation of PIDA, turnover determining step
**TS3**	Oxidation of I(III) to I(V) by oxoammonium
**TS5**	Hydrogen bond transition state for ligand exchange of alcohol on to I(V) compound, turnover determining step
$\mathbf{9_{OC}}$	I(V) resting state, turnover determining intermediate
**TS9**	Reoxidation of hydroxylamine to oxoammonium by I(V)
**TS10**	Oxidation of ligated alcohol by oxoammonium

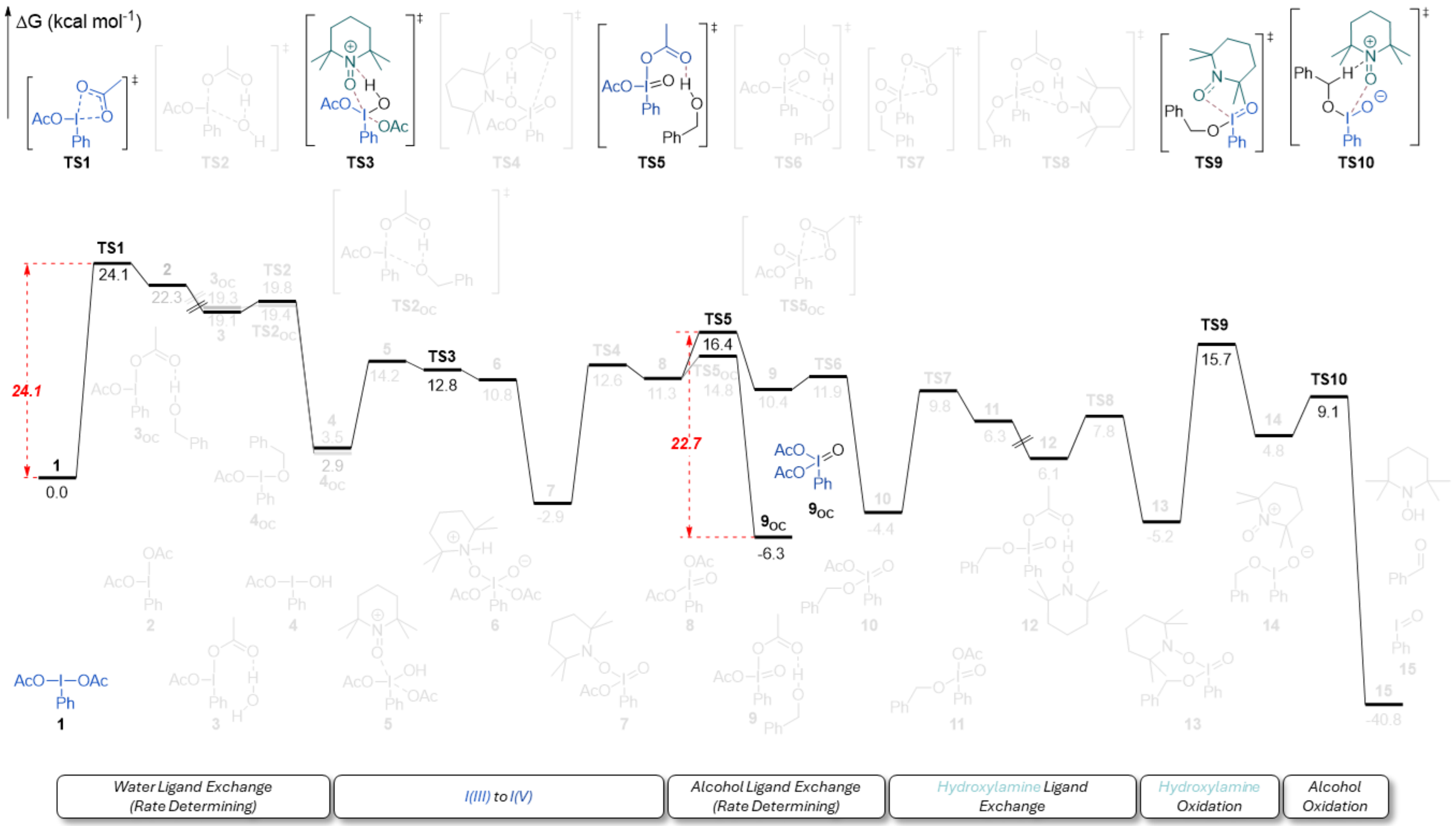

Figure S11: M06-2X/Def2TZVP/PCM$_{acetonitrile}$//M06-2X/6-31G(d,p)/SDD(I) computed Gibbs free energy (kcal mol^{-1}) profile of redox relay I(III)-I(V)-I(III) mechanism displaying only meaningful or important structures.

11.3 TS5 Hydrogen Bonds

TS5 is the formation of a hydrogen-bond between the apical acetate on hypervalent iodine and an approaching benzyl alcohol. Figure S12 shows the distance between the iodine and alcohol of the oxygen, too long for a covalent bond. The noncovalent interaction surface shows a region of hydrogen bond interaction as a blue surface between the H of the alcohol and acetate group with little to no surface between the iodine and the alcohol oxygen.

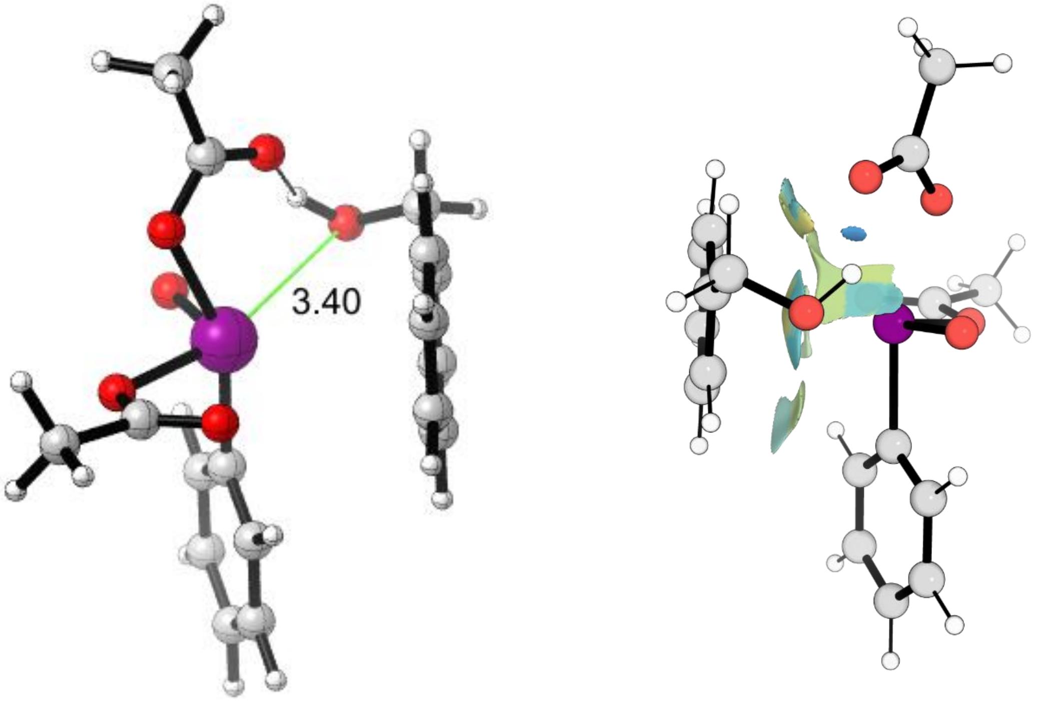

Figure S12: Structure showing distance between the iodine and alcohol oxygen, noncovalent interactions cutoffs 0.01 and 1. The color spectrum ranges from blue (strongly attractive) to green (weekly attractive) to yellow (mildly repulsive) to red (strongly repulsive).

11.4 Comparison of Alcohol Oxidation Transition States

TS10 is shown in Figure S13. During the DFT investigation, different possible alcohol oxidation transition states were calculated from various I(V), Figure S14, or I(III), Figure S15, compounds. The lowest energy transition state detailed in the main body has an energy barrier of 9.1 kcal mol^{-1}, Figure S13.

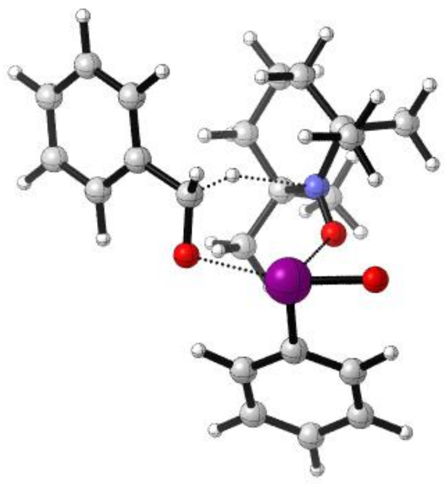

9.1 kcal mol^{-1}

Figure S13: Structure of **TS10**. Computed at M06-2X/Def2TZVP/PCM$_{acetonitrile}$//M06-2X/6-31G(d,p)/SDD(I).

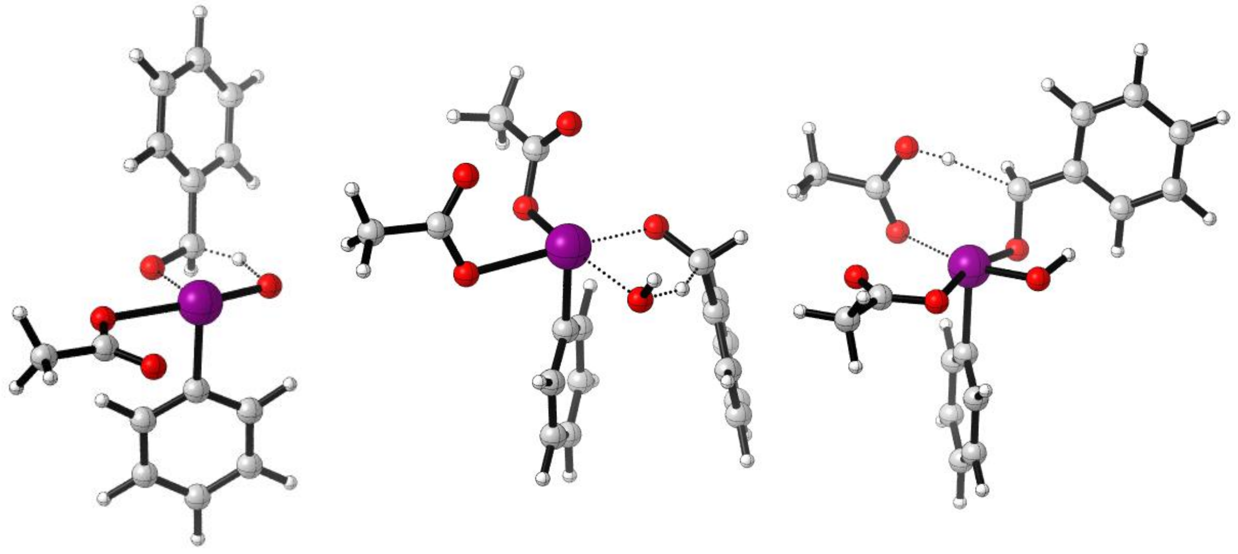

17.9 kcal mol^{-1} 46.4 kcal mol^{-1} 52.1 kcal mol^{-1}

Figure S14: Alcohol oxidation from I(V) compounds. Energies are all relative to PIDA and are Gibbs free energies (kcal mol^{-1}) computed at M06-2X/Def2TZVP/PCM$_{acetonitrile}$//M06-2X/6-31G(d,p)/SDD(I).

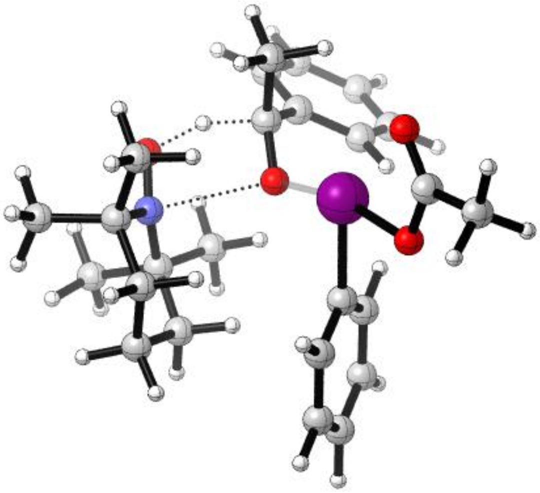

21.3 kcal mol^{-1}

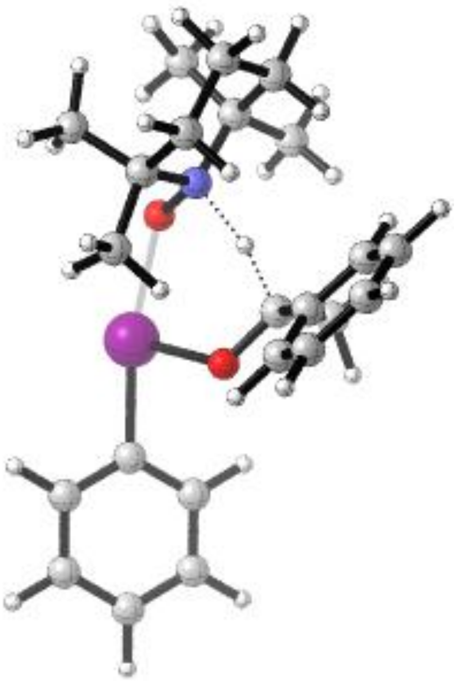

57.0 kcal mol^{-1}

Figure S15: Alcohol oxidation from I(III) compounds. Energies are all relative to PIDA and are Gibbs free energies (kcal mol^{-1}) computed at M06-2X/Def2TZVP/$PCM_{acetonitrile}$//M06-2X/6-31G(d,p)/SDD(I).

11.5 Oxidation of Hydroxylamine by PIDA via Direct Ligand Exchange Between PIDA and Hydroxylamine

A second catalytic cycle resulting in reduction of PIDA and oxidation of the hydroxylamine to the oxoammonium is required. Ligand exchange between PIDA and TEMPO (Figure S14) is only slightly higher in energy than ligand exchange between PIDA and water (Figure 10), and ultimately leads to the same conclusion.

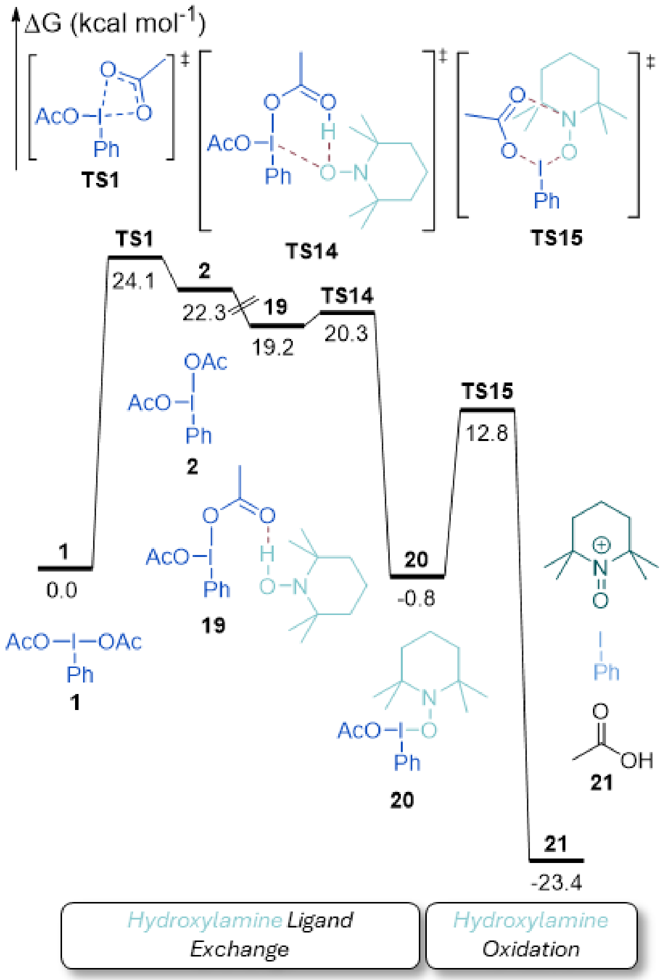

Figure S16: M06-2X/Def2TZVP/PCM$_{acetonitrile}$//M06-2X/6-31G(d,p)/SDD(I) computed Gibbs free energy (kcal mol^{-1}) profile of oxidation of hydroxylamine to oxoammonium by PIDA through direct ligand exchange between PIDA and hydroxylamine.

11.6 Catalytic Pathway for Different Alcohols

Figure 3 in the main body of the paper compares the rate equations for different alcohols (benzyl alcohol, 1-phenyl ethanol, cyclohexane methanol, cyclohexanol), showing a change for cyclohexanol and suggesting that this substrate may proceed via a different mechanism. We investigated and compared the catalytic barriers for three key transition states (TS6, TS10, and TS13). The shallow nature of the PES surrounding TS5 (H-bond forming transition state) did not allow us to locate a hydrogen-bonding transition state for these additional alcohols, and here we use TS6 (ligand exchange) as a proxy. For all the alcohols studied, very little difference is found comparing the barriers for TS6 (ligand exchange) and TS13 (oxidation of hydroxylamine to oxoammonium), which remain within 0.8 and 0.4 kcal mol^{-1} of each other respectively (Figure S17). However, in TS10 (oxidation of the alcohol by the oxoammonium) the barrier for oxidation of cyclohexanol, 18.7 kcal mol^{-1}, is 3.3 kcal mol^{-1} higher than for benzyl alcohol. This demonstrates that it is the oxidation of the sterically encumbered cyclohexanol that is more challenging than less bulky substrates. This significant outlier now means $TS10_{cyclohexanol}$ has a larger barrier than $TS6_{cyclohexanol}$. Without an investigation of the full catalytic cycle, it is not possible to comment on whether this higher barrier causes a change in rate determining step but we suggest this will be the root cause.

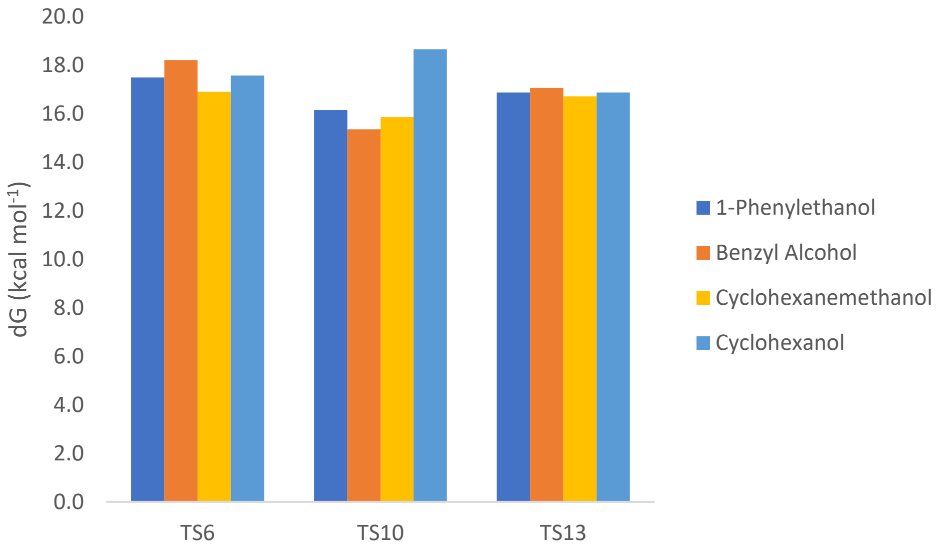

Figure S17: M06-2X/Def2TZVP/$PCM_{acetonitrile}$//M06-2X/6-31G(d,p)/SDD(I) computed Gibbs free energy (kcal mol^{-1}) catalytic barriers, relative to off-cycle intermediates, for three important transition states and four model alcohols.

12. DFT Cartesian Coordinates

12.1 Starting Materials and Products

Acetic acid dimer

Energy: -287408.6418048

C	3.38739	0.08121	0.00000
H	3.75850	-0.44727	-0.88063
H	3.75851	-0.44737	0.88057
H	3.73529	1.11164	0.00005
C	1.88548	0.04910	0.00001
O	1.38889	-1.16282	0.00001
H	0.38130	-1.12859	-0.00002
O	1.20768	1.07179	0.00000
C	-3.38736	-0.08125	0.00001
H	-3.75847	0.44708	0.88074
H	-3.73519	-1.11169	-0.00019
H	-3.75852	0.44746	-0.88046
C	-1.88547	-0.04910	0.00000
O	-1.38897	1.16285	-0.00001
H	-0.38142	1.12849	-0.00002
O	-1.20763	-1.07176	-0.00002

Benzaldehyde

Energy: -216759.0143332

C	2.20741	-0.25337	-0.00002
C	1.73262	1.05605	-0.00003
C	0.36124	1.29067	0.00000
C	-0.52852	0.21696	0.00004
C	-0.05064	-1.09617	0.00004
C	1.31747	-1.32932	0.00001
H	3.27696	-0.43850	-0.00005
H	2.42902	1.88787	-0.00006
H	-0.02466	2.30738	-0.00001
H	-0.77024	-1.90878	0.00008
H	1.69679	-2.34588	0.00002
C	-1.98941	0.47121	0.00007
H	-2.27748	1.54417	0.00019
O	-2.82892	-0.39780	-0.00010

Benzyl alcohol

Energy: -217511.1771595

C	-1.87548	0.99812	-0.01165
C	-0.51838	1.29712	-0.00470
C	0.43363	0.27546	0.00990
C	0.01079	-1.05112	0.01538
C	-1.35061	-1.35067	0.00666
C	-2.29608	-0.33086	-0.00615
H	-2.60584	1.80101	-0.02323
H	-0.19227	2.33502	-0.01144
H	0.75442	-1.83937	0.02433
H	-1.67167	-2.38768	0.00979
H	-3.35527	-0.56708	-0.01293
C	1.90086	0.63157	0.02778
H	2.11782	1.19556	0.94825
H	2.11463	1.30172	-0.81912
O	2.67568	-0.54471	-0.04276
H	3.60443	-0.29920	0.00308

Water

Energy: -47931.6451306

O	0.00000	0.00000	0.11780
H	0.00000	0.76017	-0.47122
H	0.00000	-0.76017	-0.47122

Iodobenzene

Energy: -152427.1406631

C	3.34662	0.00001	0.00000
C	2.64870	1.20411	0.00000
C	1.25539	1.21250	0.00000
C	0.57513	-0.00003	0.00000
C	1.25541	-1.21252	0.00000
C	2.64875	-1.20408	0.00000
H	4.43139	0.00005	0.00000
H	3.18563	2.14716	0.00000
H	0.71144	2.15011	0.00000
H	0.71153	-2.15016	0.00001
H	3.18566	-2.14714	-0.00001
I	-1.55860	0.00000	0.00000

Iodosobenzene

Energy: -199516.9145915

C	3.45659	0.21267	-0.00001
C	2.93066	-1.07757	-0.00010
C	1.55027	-1.27260	-0.00007
C	0.74243	-0.14746	0.00003
C	1.22651	1.14650	0.00011
C	2.60969	1.31889	0.00009
H	4.53210	0.35464	-0.00001

H	3.59220	-1.93763	-0.00026
H	1.13250	-2.27486	-0.00018
H	0.51709	1.97342	0.00007
H	3.02472	2.32151	0.00016
I	-1.39736	-0.27263	0.00004
O	-1.72943	1.61622	-0.00027

1 (PIDA)

Energy: -439007.1008037

C	0.00229	4.26451	0.00033
C	-0.96205	3.57024	0.72488
C	-0.97107	2.17714	0.73158
C	0.00081	1.51029	-0.00009
C	0.97343	2.17632	-0.73152
C	0.96590	3.56943	-0.72442
H	0.00287	5.34938	0.00049
H	-1.71379	4.10891	1.29190
H	-1.72866	1.62685	1.27546
H	1.73048	1.62536	-1.27550
H	1.71824	4.10747	-1.29123
I	-0.00033	-0.62530	-0.00047
O	-2.13135	-0.29933	-0.09743
C	-2.73662	-1.46828	-0.11990
O	-2.12687	-2.52383	-0.08568
C	-4.24153	-1.36791	-0.19333
O	2.1242	-2.52609	0.08653
C	2.73504	-1.47116	0.12042
O	2.13098	-0.30157	0.09717
C	4.24001	-1.37231	0.19447
H	-4.52461	-0.82187	-1.09541
H	-4.6731	-2.36695	-0.20423
H	-4.6116	-0.80403	0.66531
H	4.61095	-0.80636	-0.66241
H	4.6707	-2.37175	0.20302
H	4.52314	-0.829	1.09821

Oxoammonium

Energy: -303250.8288950

C	-1.24730	1.32253	-0.62695
C	-1.35263	-0.07136	0.01287
N	0.00000	-0.76995	-0.12946
C	1.35263	-0.07136	0.01287
C	1.24730	1.32253	-0.62695
C	0.00000	2.09885	-0.21969
H	-1.26997	1.20867	-1.71717
H	-2.15957	1.85712	-0.34564
H	2.15957	1.85712	-0.34564
H	1.26997	1.20867	-1.71717
H	0.00000	2.31482	0.85348
H	0.00000	3.06731	-0.72508
C	2.38910	-0.94195	-0.68876
H	2.54297	-1.89254	-0.17700
H	2.11345	-1.13485	-1.72824
H	3.33097	-0.38927	-0.68202
C	1.64396	-0.01055	1.52537
H	1.04464	0.73186	2.04975
H	1.5124	-0.989	1.99382
H	2.69468	0.27238	1.62416
C	-1.64396	-0.01055	1.52537
H	-1.5124	-0.989	1.99382
H	-1.04464	0.73186	2.04975
H	-2.69468	0.27238	1.62416
C	-2.3891	-0.94195	-0.68876
H	-2.11345	-1.13485	-1.72824
H	-2.54297	-1.89254	-0.177
H	-3.33097	-0.38927	-0.68202
O	0	-1.9373	-0.32622

Hydroxylamine

Energy: -303791.1177343

C	-1.24456	1.42366	-0.45994
C	-1.28491	-0.05425	-0.04330
N	0.00000	-0.65973	-0.45706
C	1.28491	-0.05424	-0.04330
C	1.24456	1.42367	-0.45994
C	-0.00001	2.14931	0.04314
H	-1.25781	1.46459	-1.55609
H	-2.15982	1.91013	-0.10424
H	2.15981	1.91014	-0.10423
H	1.25781	1.46460	-1.55609
H	-0.00001	2.20134	1.13847
H	-0.00001	3.18431	-0.31288
C	2.37862	-0.76652	-0.84622
H	2.48001	-1.80641	-0.53019
H	2.12079	-0.75553	-1.90777
H	3.33766	-0.25878	-0.70442
C	1.62152	-0.18508	1.45528
H	1.02225	0.46747	2.09158

H	1.50224	-1.21563	1.80713
H	2.67217	0.07649	1.61097
C	-1.62152	-0.18509	1.45528
H	-1.50224	-1.21563	1.80713
H	-1.02225	0.46747	2.09158
H	-2.67217	0.07649	1.61097
C	-2.37862	-0.76653	-0.84622
H	-2.12079	-0.75554	-1.90776
H	-2.48001	-1.80642	-0.53019
H	-3.33766	-0.25879	-0.70442
O	0.00001	-2.03876	-0.17072
H	0.00002	-2.13165	0.7968

12.2 Cycle 1: Alcohol Oxidation

TS1

Energy: -438981.2030029

C	4.33091	-1.22811	0.12592
C	3.70246	-0.58118	1.18808
C	2.34135	-0.29694	1.12518
C	1.62072	-0.66132	-0.01176
C	2.24390	-1.30930	-1.07679
C	3.60522	-1.59302	-1.00531
H	5.39179	-1.44991	0.18071
H	4.27214	-0.29665	2.06651
H	1.84621	0.22132	1.93981
H	1.67459	-1.59276	-1.95723
H	4.09682	-2.09695	-1.83082
I	-0.49144	-0.27282	-0.10576
O	-0.14543	1.60027	-0.83184
C	0.25402	2.51227	0.08102
O	0.49164	2.25399	1.23197
C	0.35799	3.87182	-0.56144
H	0.73597	4.58076	0.17247
H	1.0219	3.82105	-1.42595
H	-0.62958	4.17579	-0.91353
O	-2.87202	0.26265	-0.23534
C	-3.254	-0.88908	0.14545
O	-2.45043	-1.82047	0.41469
C	-4.74244	-1.12337	0.28548
H	-4.9439	-2.16907	0.51416
H	-5.12674	-0.48731	1.08655
H	-5.24398	-0.82905	-0.63843

2

Energy: -438984.1172083

C	4.59877	0.25890	-0.04974
C	3.80909	0.78566	0.97007
C	2.44687	0.50207	1.01945
C	1.88783	-0.32225	0.04558
C	2.67126	-0.85150	-0.97796
C	4.03211	-0.55780	-1.02422
H	5.65909	0.48712	-0.08550
H	4.25184	1.42551	1.72628
H	1.81940	0.93408	1.79099
H	2.22842	-1.48941	-1.73749
H	4.64569	-0.96526	-1.82097
I	-0.17409	-0.92093	0.16062
O	-0.61838	0.70798	-0.94271
C	-0.61945	1.87687	-0.24044
O	-0.12426	2.00977	0.84382
C	-1.33051	2.93787	-1.03352
O	-2.86665	0.78186	0.71524
C	-3.19239	-0.26502	0.17226
O	-2.36452	-1.18754	-0.23528
C	-4.6458	-0.61321	-0.09896
H	-2.39558	2.79053	-0.83676
H	-1.01724	3.91708	-0.67572
H	-1.14605	2.82859	-2.10179
H	-4.79502	-0.72896	-1.17475
H	-5.29196	0.1729	0.28893
H	-4.88786	-1.56885	0.37025

3

Energy: -486934.1423342

C	2.46101	0.00488	1.06852
C	3.84928	-0.09493	1.11375
C	1.78589	-0.42394	-0.07156
C	4.54861	-0.61969	0.02958
H	4.38369	0.23788	1.99741
C	2.47777	-0.94525	-1.16133
C	3.86593	-1.04467	-1.10724
H	5.63025	-0.69693	0.07010
H	4.41194	-1.45020	-1.95266
H	1.94324	-1.27048	-2.04910
H	1.90816	0.42500	1.90246
O	-0.15948	1.58398	-0.78559
C	0.01743	2.51567	0.17417

C	-0.04300	3.89541	-0.42620
O	0.19921	2.25785	1.33615
H	0.64608	3.96521	-1.26923
H	-1.05356	4.06775	-0.80226
H	0.20719	4.62777	0.3387
I	-0.35224	-0.31159	-0.12142
O	-2.61733	0.47746	-0.30443
C	-3.32449	-0.53711	0.00155
O	-2.85907	-1.66471	0.28034
O	-0.44087	-2.78649	0.6674
H	-1.41448	-2.65849	0.69646
C	-4.82615	-0.32284	0.02086
H	-5.07008	0.41478	0.78897
H	-5.14744	0.08658	-0.93892
H	-5.34244	-1.25929	0.22713
H	-0.28719	-3.48516	0.02243

3_{OC}

Energy: -656513.3950667

C	-1.68690	2.37500	1.03021
C	-1.76363	3.76555	1.03146
C	-1.21153	1.72255	-0.10425
C	-1.36722	4.48969	-0.09047
H	-2.13399	4.28254	1.91052
C	-0.81953	2.43863	-1.23255
C	-0.89573	3.82948	-1.22207
H	-1.42725	5.57313	-0.08345
H	-0.59075	4.39394	-2.09705
C	2.23355	0.32121	-0.55685
C	3.70263	0.28362	-0.20889
C	4.12833	0.59059	1.08377
C	4.64809	-0.02767	-1.18537
C	5.48513	0.58960	1.39238
H	3.38649	0.81735	1.84271
C	6.00594	-0.02100	-0.87916
H	4.3196	-0.28421	-2.18944
C	6.42697	0.28737	0.41155
H	5.80846	0.82412	2.40156
H	6.73375	-0.26823	-1.6453
H	7.48468	0.28549	0.65408
H	-0.45831	1.9193	-2.11582
H	-2.00658	1.80121	1.89379
O	-2.96141	-0.52483	-0.7052
C	-3.88354	-0.40805	0.27145
C	-5.25961	-0.68091	-0.2768
O	-3.62259	-0.1227	1.41238
H	-5.44406	-0.05021	-1.14784
H	-5.30723	-1.72275	-0.60006
H	-5.99688	-0.49035	0.50048
I	-1.03512	-0.4101	-0.09933
O	-1.41073	-2.80646	-0.22359
C	-0.25808	-3.28639	0.01474
O	0.76546	-2.59409	0.23186
O	1.40739	-0.03497	0.54404
H	1.47075	-1.01127	0.65851
H	2.03231	-0.33832	-1.41273
H	1.93743	1.33962	-0.83597
C	-0.14687	-4.7987	0.05599
H	-0.54721	-5.15054	1.01075
H	-0.74807	-5.24026	-0.73959
H	0.89543	-5.10425	-0.02743

TS2

Energy: -486933.7145647

C	2.46425	-0.15871	1.09009
C	3.84047	-0.36580	1.13481
C	1.76760	-0.50557	-0.06401
C	4.50279	-0.91111	0.03759
H	4.39436	-0.09862	2.02852
C	2.41980	-1.04614	-1.16847
C	3.79616	-1.25083	-1.11318
H	5.57533	-1.07054	0.07863
H	4.31370	-1.67216	-1.96854
H	1.86533	-1.30520	-2.06544
H	1.94036	0.28152	1.93149
O	-0.09844	1.67351	-0.78267
C	0.23374	2.57501	0.16280
C	0.21904	3.96703	-0.41212
O	0.50430	2.28558	1.30082
H	0.85511	4.00815	-1.29782
H	-0.79997	4.21021	-0.71968
H	0.56569	4.6688	0.34378
I	-0.34628	-0.22918	-0.12877
O	-2.74047	0.46437	-0.29051
C	-3.432	-0.54358	0.0205
O	-2.97987	-1.68288	0.32558
O	-0.63016	-2.5561	0.60536
H	-1.63219	-2.35163	0.57777

C	-4.94101	-0.37779	0.05391
H	-5.26339	-0.34986	1.09831
H	-5.24161	0.54466	-0.44062
H	-5.41532	-1.24184	-0.414
H	-0.47228	-3.2477	-0.04736

TS2$_{oc}$

Energy: -656513.8196459

C	0.66158	2.47319	-1.47304
C	0.53256	3.85901	-1.43014
C	1.01652	1.78762	-0.31507
C	0.76478	4.54307	-0.23958
H	0.25338	4.40194	-2.32696
C	1.24341	2.45948	0.88363
C	1.11972	3.84682	0.91313
H	0.66607	5.62324	-0.20970
H	1.29790	4.38166	1.84014
C	-1.98983	0.09571	0.61757
C	-3.49327	0.06617	0.47767
C	-4.09648	0.29053	-0.75920
C	-4.29267	-0.15286	1.59963
C	-5.48405	0.29893	-0.87008
H	-3.46862	0.44563	-1.63027
C	-5.67976	-0.13705	1.49055
H	-3.82643	-0.34314	2.5632
C	-6.27883	0.08861	0.25373
H	-5.94597	0.46845	-1.83757
H	-6.29262	-0.31191	2.369
H	-7.36039	0.0938	0.16547
H	1.52376	1.90653	1.77444
H	0.47799	1.93446	-2.39733
O	3.14926	-0.28138	-0.29737
C	3.69314	-0.17752	0.93069
C	5.18457	-0.37398	0.86162
O	3.05831	0.04492	1.93116
H	5.38694	-1.40003	0.5475
H	5.61331	0.29608	0.11483
H	5.61572	-0.18556	1.84277
I	1.12383	-0.34524	-0.36749
O	1.62766	-2.77958	-0.31563
C	0.48503	-3.31673	-0.35261
O	-0.60837	-2.69234	-0.43866
O	-1.33038	-0.2428	-0.59669
H	-1.29235	-1.24452	-0.65696
H	-1.66978	-0.58399	1.41898
H	-1.65586	1.10753	0.88071
C	0.41441	-4.83226	-0.30763
H	0.09444	-5.19543	-1.28778
H	1.38396	-5.26089	-0.05893
H	-0.34115	-5.13885	0.41781

4

Energy: -343240.9838689

O	0.60969	-2.63107	-0.11946
C	2.46769	-0.50329	0.38553
C	3.68239	0.18051	0.40191
C	1.33375	0.19049	-0.00697
C	3.74285	1.51946	0.02852
H	4.58093	-0.34294	0.71140
C	1.35737	1.52553	-0.38187
C	2.58512	2.18572	-0.36214
H	4.69215	2.04445	0.04264
H	2.62765	3.22943	-0.65546
I	-0.51455	-0.91516	-0.03388
H	0.44767	2.03840	-0.66333
H	2.40021	-1.55065	0.65699
O	-1.37371	1.10376	0.12399
C	-2.67986	1.00486	0.09046
C	-3.39525	2.33249	0.20645
O	-3.26717	-0.06183	-0.02218
H	-3.1181	2.8121	1.1475
H	-3.07781	2.99037	-0.60536
H	-4.47159	2.1753	0.16558
H	0.87532	-2.79437	-1.03452

4$_{oc}$

Energy: -512821.9447857

O	-0.87863	-1.04990	-0.37455
C	-0.47016	1.76074	-0.70872
C	-0.77771	3.12005	-0.68383
C	0.71858	1.35024	-0.12476
C	0.09164	4.02907	-0.08883
H	-1.70405	3.46016	-1.13434
C	1.60641	2.22873	0.47797
C	1.27665	3.58328	0.48880
H	-0.15511	5.08540	-0.07370
H	1.95642	4.28801	0.95616
I	1.14542	-0.76222	-0.15678

C	-1.65063	-0.85925	0.78664
H	-1.35597	0.06263	1.31792
C	-3.11595	-0.74660	0.42702
C	-3.55653	-0.93944	-0.88019
C	-4.04597	-0.44118	1.42381
C	-4.91225	-0.82611	-1.18653
H	-2.82879	-1.18236	-1.64647
C	-5.39722	-0.33055	1.11872
H	-3.70607	-0.28752	2.44566
C	-5.83484	-0.5222	-0.19136
H	-5.24672	-0.97886	-2.20798
H	-6.11031	-0.09196	1.90154
H	-6.88953	-0.43492	-0.43153
H	2.53254	1.87269	0.90868
H	-1.14673	1.03788	-1.15196
O	3.20892	-0.04278	0.03004
C	4.00955	-1.07888	0.10984
C	5.4706	-0.71128	0.23825
O	3.61028	-2.23368	0.08029
H	5.77165	-0.11564	-0.62596
H	5.61552	-0.09721	1.12958
H	6.07246	-1.61605	0.30156
H	-1.51004	-1.69353	1.4927

5

Energy: -789910.5128488

I	0.68947	-0.46357	-0.78587
C	1.83522	0.68856	0.62486
C	1.95739	0.17006	1.90050
C	2.48798	1.82278	0.17701
C	2.74773	0.88135	2.80151
H	1.48754	-0.76708	2.16799
C	3.28591	2.50248	1.09357
H	2.38270	2.16853	-0.84147
C	3.40373	2.04114	2.40175
H	2.86001	0.50623	3.81264
H	3.80721	3.39901	0.77693
H	4.02234	2.58224	3.11014
H	-0.82561	-1.41392	0.79471
O	-1.24256	0.30526	-0.56955
N	-2.06523	-0.38681	0.30754
C	-2.51515	0.56330	1.37895
C	-3.08956	-1.13972	-0.48873
C	-3.44142	-0.22573	2.31354
C	-3.17876	1.82977	0.82385
C	-1.26171	0.99418	2.14113
C	-3.99267	-1.86724	0.51737
C	-2.34242	-2.17926	-1.33335
C	-3.89855	-0.24899	-1.44384
C	-4.57385	-0.93596	1.57778
H	-2.83924	-0.97375	2.84674
H	-3.83263	0.46716	3.06607
H	-2.5394	2.27156	0.05507
H	-4.17597	1.6565	0.41914
H	-3.275	2.55523	1.63698
H	-0.70269	0.12332	2.4967
H	-0.62046	1.6129	1.50629
H	-1.55509	1.58546	3.01308
H	-4.78647	-2.37269	-0.04356
H	-3.40079	-2.6481	1.01343
H	-1.77703	-1.69812	-2.13829
H	-1.66579	-2.78652	-0.72582
H	-3.06882	-2.84697	-1.80392
H	-4.70958	0.28419	-0.94791
H	-3.24051	0.47891	-1.92449
H	-4.34589	-0.87463	-2.22164
H	-5.24864	-0.20494	1.11852
H	-5.17769	-1.51131	2.2859
O	0.65364	1.33279	-2.0545
C	0.0333	2.45514	-1.72625
O	-0.12164	2.86114	-0.58981
C	-0.47494	3.20827	-2.9386
H	0.31379	3.29278	-3.68822
H	-1.29326	2.63729	-3.38567
H	-0.83178	4.19305	-2.64048
O	0.1379	-1.69344	0.62468
O	3.01932	-2.27158	1.03226
C	3.27751	-2.20788	-0.15141
O	2.53825	-1.59979	-1.06577
C	4.51462	-2.84268	-0.75809
H	5.08598	-3.34769	0.01949
H	4.21851	-3.5529	-1.53289
H	5.12324	-2.07109	-1.23482

TS3

Energy: -789907.5618287

I	0.70230	-0.47118	-0.78041
C	1.84243	0.68050	0.63135

C	1.96532	0.12945	1.89352
C	2.48498	1.83083	0.21298
C	2.75486	0.81776	2.81259
H	1.48659	-0.81231	2.13261
C	3.28040	2.48942	1.14771
H	2.36820	2.20182	-0.79549
C	3.40455	1.99147	2.44197
H	2.87050	0.41796	3.81397
H	3.79402	3.39923	0.85720
H	4.02167	2.51664	3.16371
H	-1.16303	-1.12645	0.65756
O	-1.27297	0.34354	-0.64626
N	-2.04590	-0.36040	0.26144
C	-2.48990	0.57586	1.36988
C	-3.0884	-1.16841	-0.48787
C	-3.36348	-0.24261	2.32871
C	-3.19513	1.81533	0.8173
C	-1.22038	1.03707	2.08399
C	-3.92767	-1.9093	0.56123
C	-2.33737	-2.18552	-1.3543
C	-3.9443	-0.29178	-1.40769
C	-4.50009	-0.98513	1.63265
H	-2.72396	-0.97194	2.84445
H	-3.74649	0.44089	3.09354
H	-2.57504	2.27194	0.0415
H	-4.19392	1.61629	0.42983
H	-3.29534	2.53588	1.63382
H	-0.63182	0.18254	2.42829
H	-0.61663	1.66706	1.42344
H	-1.50544	1.62691	2.95943
H	-4.72338	-2.44532	0.03285
H	-3.29526	-2.66794	1.04196
H	-1.80647	-1.6843	-2.16951
H	-1.63077	-2.77712	-0.76708
H	-3.06601	-2.86371	-1.80536
H	-4.75433	0.21695	-0.88585
H	-3.31875	0.45179	-1.90626
H	-4.39684	-0.92997	-2.17153
H	-5.20846	-0.27524	1.19158
H	-5.06431	-1.57189	2.36328
O	0.66975	1.41284	-2.04213
C	0.03577	2.50951	-1.70155
O	-0.1326	2.90201	-0.55616
C	-0.49704	3.28208	-2.89496
H	0.27872	3.38463	-3.65581
H	-1.31834	2.71242	-3.33902
H	-0.85799	4.25962	-2.57761
O	0.02044	-1.59875	0.57716
O	2.93728	-2.36123	0.97537
C	3.24307	-2.2393	-0.19126
O	2.54789	-1.56835	-1.0989
C	4.49258	-2.85746	-0.78772
H	5.02486	-3.41764	-0.02037
H	4.21742	-3.51417	-1.6155
H	5.13063	-2.06845	-1.19181

6

Energy: -789908.4951737

I	0.75101	-0.56917	-0.71605
C	1.82523	0.74132	0.60480
C	1.95623	0.28140	1.90235
C	2.40837	1.89669	0.12013
C	2.69249	1.06839	2.78500
H	1.51827	-0.66691	2.19317
C	3.15227	2.65679	1.02043
H	2.28053	2.19402	-0.91120
C	3.28335	2.25011	2.34551
H	2.81435	0.74203	3.81206
H	3.61866	3.57436	0.67900
H	3.85931	2.85443	3.03895
H	-1.36811	-1.03735	0.76316
O	-1.27171	0.23846	-0.67543
N	-2.06436	-0.36793	0.27732
C	-2.51663	0.66707	1.30617
C	-3.10778	-1.24905	-0.40563
C	-3.4041	-0.06561	2.31934
C	-3.20341	1.85123	0.63066
C	-1.24208	1.17179	1.97846
C	-3.9594	-1.88406	0.70038
C	-2.33488	-2.3338	-1.16071
C	-3.93935	-0.44718	-1.40671
C	-4.53644	-0.86225	1.67719
H	-2.7745	-0.74772	2.90738
H	-3.79299	0.6837	3.01626
H	-2.56837	2.23088	-0.17418
H	-4.20211	1.62765	0.25615
H	-3.30124	2.64258	1.37896
H	-0.64955	0.33906	2.36863

H	-0.64625	1.75853	1.27221
H	-1.52226	1.81295	2.81859
H	-4.75319	-2.4611	0.21443
H	-3.33818	-2.60144	1.25392
H	-1.80984	-1.90535	-2.01919
H	-1.61595	-2.84322	-0.5138
H	-3.05021	-3.06821	-1.53916
H	-4.75793	0.10323	-0.94398
H	-3.29978	0.24958	-1.95233
H	-4.37756	-1.14729	-2.12287
H	-5.23492	-0.19101	1.16588
H	-5.11252	-1.37786	2.45076
O	0.64967	1.28211	-2.12854
C	-0.03474	2.36202	-1.87988
O	-0.22375	2.84879	-0.76913
C	-0.62436	3.00728	-3.12473
H	0.12779	3.06644	-3.91339
H	-1.43552	2.37242	-3.49369
H	-1.01482	3.99679	-2.88898
O	0.11517	-1.62129	0.67526
O	3.0943	-2.18347	1.09596
C	3.36641	-2.14348	-0.08247
O	2.62058	-1.57046	-1.02286
C	4.62176	-2.75521	-0.67036
H	5.20149	-3.22659	0.12177
H	4.34978	-3.48986	-1.43097
H	5.21075	-1.97662	-1.1598

7

Energy: -646205.4956826

I	-1.17235	-0.71339	-0.00149
C	-0.86333	1.39429	0.05109
C	-0.85238	2.09056	1.24711
C	-0.83054	1.99814	-1.19005
C	-0.74100	3.47758	1.18749
H	-0.93991	1.58181	2.20130
C	-0.71532	3.38677	-1.23046
H	-0.91052	1.39113	-2.08866
C	-0.66131	4.11885	-0.04731
H	-0.72505	4.05410	2.10608
H	-0.67846	3.89399	-2.18858
H	-0.57466	5.19960	-0.08564
O	-1.37669	-0.90282	-1.80147
O	0.80895	-1.25164	-0.00274
N	1.83966	-0.29542	0.04572
C	2.54035	-0.49159	1.34801
C	2.63679	-0.41724	-1.21161
C	3.73593	0.47227	1.38519
C	2.98211	-1.93852	1.62847
C	1.55903	-0.06913	2.44374
C	3.83485	0.53774	-1.09189
C	1.74389	0.07312	-2.35213
C	3.09024	-1.85068	-1.53331
C	4.65262	0.32359	0.17627
H	3.34959	1.49947	1.40942
H	4.27994	0.30583	2.32195
H	2.19018	-2.63456	1.34449
H	3.89392	-2.21497	1.09862
H	3.17878	-2.05628	2.69851
H	1.22955	0.9571	2.2685
H	0.68924	-0.73545	2.4742
H	2.04331	-0.11711	3.42312
H	4.45278	0.41914	-1.98895
H	3.45348	1.56692	-1.09417
H	0.85003	-0.54842	-2.45351
H	1.44013	1.10657	-2.16405
H	2.30014	0.04488	-3.29389
H	3.93522	-2.18048	-0.92735
H	2.26034	-2.54516	-1.38759
H	3.3998	-1.90262	-2.58119
H	5.12625	-0.66449	0.16958
H	5.46507	1.05514	0.22954
O	-3.19311	0.01463	0.18238
C	-4.0042	-1.02303	0.24745
O	-3.59858	-2.17513	0.237
C	-5.46422	-0.6536	0.32956
H	-5.75376	-0.16353	-0.60278
H	-6.06278	-1.54983	0.4822
H	-5.62143	0.05951	1.14069

TS4

Energy: -789905.4857701

C	0.74876	2.49660	-1.39488
C	0.56164	3.87624	-1.35624
C	1.05713	1.84082	-0.21222
C	0.68663	4.56344	-0.15047
H	0.31553	4.41301	-2.26623
C	1.19519	2.50589	1.00049

C	1.00521	3.88479	1.02456
H	0.53442	5.63757	-0.12529
H	1.10379	4.42638	1.95919
H	1.44620	1.96215	1.90639
H	0.64850	1.92498	-2.31332
O	3.21784	-0.16472	-0.65211
C	3.89573	0.06462	0.47134
C	5.38325	0.03101	0.26925
O	3.33176	0.27438	1.52889
H	5.67093	-0.96773	-0.06540
H	5.66054	0.73813	-0.51451
H	5.88017	0.27822	1.20514
O	1.64844	-2.63883	-0.2969
C	0.85672	-3.33454	0.41247
O	-0.1705	-2.90618	0.99908
H	-0.77135	-1.60618	0.82663
C	1.17914	-4.80867	0.53116
H	0.90284	-5.29539	-0.40808
H	2.25166	-4.94581	0.67236
H	0.61585	-5.25721	1.34806
O	-1.01867	-0.61736	0.59388
N	-2.42932	-0.58236	0.44667
C	-2.96199	0.54745	1.2332
C	-2.82555	-0.76189	-0.96681
C	-4.49338	0.40476	1.18735
C	-2.5157	1.94529	0.76832
C	-2.49326	0.34549	2.67828
C	-4.35848	-0.88888	-0.94858
C	-2.21653	-2.08779	-1.43792
C	-2.37763	0.35179	-1.92884
C	-5.03983	0.275	-0.23223
H	-4.76514	-0.49261	1.75654
H	-4.93404	1.26598	1.70244
H	-1.44841	1.94846	0.53331
H	-3.06039	2.30017	-0.10758
H	-2.68179	2.66567	1.57562
H	-2.6623	-0.6894	2.98657
H	-1.42868	0.5685	2.7803
H	-3.05154	1.01183	3.34282
H	-4.7077	-0.97177	-1.98377
H	-4.61544	-1.82514	-0.43815
H	-1.14639	-1.98296	-1.63938
H	-2.36561	-2.86387	-0.68241
H	-2.70139	-2.39647	-2.36898
H	-3.00052	1.24565	-1.87106
H	-1.33868	0.62696	-1.74122
H	-2.4257	-0.02384	-2.95546
H	-4.88594	1.20772	-0.78703
H	-6.12192	0.11306	-0.20036
I	1.21002	-0.27605	-0.23585
O	0.77041	-0.42444	-1.97827

8

Energy: -486095.6435674

C	-4.52952	-0.47771	-0.13084
C	-3.83765	0.21180	-1.12381
C	-2.44459	0.22998	-1.11385
C	-1.77128	-0.44840	-0.10372
C	-2.44602	-1.14237	0.89102
C	-3.83824	-1.15354	0.87315
H	-5.61461	-0.49089	-0.14134
H	-4.37943	0.73494	-1.90479
H	-1.89683	0.77684	-1.87704
H	-1.87837	-1.66784	1.65312
H	-4.38412	-1.69075	1.64175
O	0.14884	1.25641	0.97466
C	0.12726	2.28527	0.13418
O	0.14325	2.11918	-1.07208
C	0.07934	3.61138	0.83539
H	-0.03032	4.40418	0.09841
H	-0.75057	3.62124	1.54371
H	1.00515	3.74039	1.39988
O	2.40342	0.21673	0.39075
C	3.21338	-0.59883	-0.23664
O	2.79973	-1.5215	-0.93333
C	4.67978	-0.31409	-0.03018
H	5.27562	-0.98148	-0.65043
H	4.89059	0.72897	-0.27306
H	4.92232	-0.46503	1.02412
I	0.37395	-0.46597	-0.12582
O	0.45808	-1.72524	1.1632

TS5

Energy: -703626.3659394

C	2.54735	0.60894	-1.70339
C	3.90180	0.93956	-1.74511
C	2.01616	0.13981	-0.51246
C	4.69384	0.78801	-0.61101

H	4.33588	1.31223	-2.66695
C	2.79055	-0.01930	0.63169
C	4.14221	0.30683	0.57605
H	5.74749	1.04485	-0.64972
H	4.76371	0.18583	1.45727
C	-2.06418	3.02268	-0.42825
C	-1.38802	2.44787	0.79888
C	-0.01958	2.67709	0.98335
C	-2.05800	1.60116	1.68546
C	0.66871	2.06404	2.02923
H	0.50577	3.32266	0.28399
C	-1.36817	0.96839	2.71673
H	-3.11247	1.39905	1.52433
C	-0.00137	1.19257	2.88634
H	1.73098	2.24763	2.15714
H	-1.88779	0.28179	3.37698
H	0.53625	0.68283	3.67928
H	2.34735	-0.38884	1.55335
H	1.90636	0.70751	-2.57387
O	0.61218	-2.25325	-0.32989
C	0.57315	-2.68055	0.93178
C	0.82884	-4.1557	1.04334
O	0.35274	-1.92417	1.857
H	-0.01669	-4.68229	0.59507
H	1.72632	-4.42117	0.48288
H	0.92986	-4.42588	2.0925
O	-1.87158	-1.68942	-0.46572
C	-2.92158	-0.97451	-0.72025
O	-2.88409	0.24989	-0.87482
O	-1.43405	2.55809	-1.60318
H	-1.81719	1.69166	-1.81344
H	-3.13065	2.77264	-0.41381
C	-4.21374	-1.75177	-0.81682
H	-4.15479	-2.42589	-1.67435
H	-4.34326	-2.36468	0.07712
H	-5.05217	-1.06795	-0.93856
H	-1.96527	4.11384	-0.4264
I	-0.07468	-0.32937	-0.44222
O	-0.25764	-0.13864	-2.22818

TS5$_{OC}$

Energy: -486092.9419929

C	-4.34903	-1.11458	-0.24856
C	-3.70993	-0.44657	-1.29093
C	-2.34527	-0.18171	-1.21076
C	-1.64949	-0.59365	-0.07885
C	-2.26915	-1.26591	0.96615
C	-3.63417	-1.52378	0.87597
H	-5.41246	-1.32065	-0.31483
H	-4.27137	-0.13044	-2.16362
H	-1.83890	0.35266	-2.00958
H	-1.67828	-1.57741	1.82200
H	-4.13975	-2.04604	1.68136
O	0.11939	1.59997	0.91321
C	-0.19162	2.51311	-0.00903
O	-0.32168	2.22345	-1.18180
C	-0.35519	3.88646	0.57672
H	-0.64487	4.57976	-0.21022
H	-1.11112	3.8573	1.36352
H	0.58852	4.19401	1.03141
O	2.75697	0.26549	0.24312
C	3.14131	-0.81028	-0.32337
O	2.31679	-1.67934	-0.71274
C	4.62024	-1.03535	-0.50217
H	4.79821	-1.8175	-1.23862
H	5.10552	-0.10293	-0.79216
H	5.03421	-1.3462	0.46057
I	0.45625	-0.22062	0.02059
O	0.60891	-1.09911	1.58171

9

Energy: -703629.3699966

C	1.63574	-2.03275	-1.61852
C	2.95510	-2.47235	-1.69143
C	1.27172	-1.21177	-0.56183
C	3.87461	-2.08419	-0.71790
H	3.26623	-3.11589	-2.50768
C	2.17043	-0.81008	0.41821
C	3.48757	-1.25489	0.33239
H	4.90204	-2.42836	-0.78106
H	4.20777	-0.94756	1.08383
C	0.96733	2.39726	-1.78793
C	1.38628	2.42326	-0.33436
C	2.73570	2.43682	0.01621
C	0.41794	2.41449	0.67502
C	3.11789	2.43775	1.35584
H	3.49194	2.42742	-0.76429
C	0.79856	2.39047	2.01433

H	-0.63826	2.42571	0.40766
C	2.15009	2.40334	2.35691
H	4.17155	2.44678	1.6168
H	0.03977	2.35141	2.78831
H	2.44568	2.38538	3.40077
H	1.856	-0.15703	1.22966
H	0.89281	-2.30625	-2.36237
O	-1.1122	-2.06854	0.79339
C	-0.94776	-1.68802	2.06032
C	-1.39732	-2.73415	3.03795
O	-0.4824	-0.60173	2.34656
H	-2.4788	-2.85316	2.9424
H	-0.93179	-3.69044	2.79464
H	-1.13761	-2.42131	4.04718
O	-2.98833	-0.21422	0.12606
C	-3.3057	0.92861	-0.35196
O	-2.51046	1.66468	-0.97648
O	-0.03589	1.42252	-2.05034
H	-0.93087	1.80207	-1.9498
H	0.60396	3.38404	-2.09893
C	-4.73721	1.36871	-0.13972
H	-5.38078	0.76562	-0.78521
H	-5.03538	1.18081	0.89268
H	-4.85192	2.42165	-0.39255
H	1.82656	2.14571	-2.41555
I	-0.72939	-0.49894	-0.44785
O	-1.26406	-1.30865	-1.96315

9_{OC}

Energy: -486110.0469572

I	-0.56572	0.02351	-0.07314
C	1.54442	-0.08600	-0.09091
C	2.22472	-0.52156	-1.21489
C	2.16400	0.28645	1.08727
C	3.61531	-0.57189	-1.15183
H	1.69773	-0.82565	-2.11387
C	3.55484	0.22893	1.13228
H	1.56350	0.60027	1.93705
C	4.27364	-0.19565	0.01702
H	4.18036	-0.90918	-2.01398
H	4.07551	0.51377	2.04019
H	5.35689	-0.23811	0.05904
O	-0.82612	0.11342	1.71974
O	-0.35452	-2.08235	-0.04246
O	-0.13810	2.09518	-0.15152
O	-2.56969	-1.93530	-0.13982
O	-2.35383	2.16721	-0.30443
C	-1.56524	-4.12251	0.03639
C	-1.13417	4.24807	-0.22834
C	-1.56518	-2.62058	-0.05902
C	-1.2861	2.75078	-0.23407
H	-1.17212	-4.40955	1.01423
H	-2.57996	-4.49681	-0.08396
H	-0.90427	-4.54256	-0.72361
H	-0.4277	4.5508	-1.00318
H	-2.10432	4.7149	-0.38678
H	-0.72195	4.55465	0.73554

TS6

Energy: -703628.6217794

C	1.82283	-1.78343	-1.72240
C	3.17837	-2.07482	-1.85125
C	1.41181	-1.05398	-0.61705
C	4.08105	-1.63510	-0.88414
H	3.52958	-2.64356	-2.70570
C	2.28952	-0.60325	0.36002
C	3.64219	-0.90146	0.21604
H	5.13656	-1.86421	-0.99067
H	4.34914	-0.55509	0.96284
C	0.44831	2.44777	-1.66932
C	1.02182	2.49329	-0.26925
C	2.39133	2.65846	-0.06542
C	0.17813	2.35769	0.83862
C	2.91590	2.68515	1.22489
H	3.05278	2.75015	-0.92283
C	0.70401	2.35720	2.12809
H	-0.89629	2.26423	0.68418
C	2.0747	2.52309	2.32322
H	3.98376	2.81453	1.37098
H	0.04372	2.22243	2.97796
H	2.48359	2.52634	3.32833
H	1.93161	-0.0276	1.21
H	1.08756	-2.10271	-2.45565
O	-0.92076	-2.23307	0.76719
C	-0.67545	-1.90797	2.03554
C	-1.01293	-3.01487	2.99201
O	-0.22626	-0.81988	2.34332
H	-2.08583	-3.21039	2.9373

H	-0.49186	-3.92632	2.69396
H	-0.7297	-2.72031	4.00045
O	-3.02006	-0.31435	0.04863
C	-3.42739	0.82767	-0.3142
O	-2.72747	1.70781	-0.88876
O	-0.44719	1.36177	-1.87004
H	-1.40452	1.61246	-1.5943
H	-0.08545	3.38094	-1.88827
C	-4.88401	1.14977	-0.05303
H	-5.47276	0.74462	-0.8808
H	-5.21962	0.67064	0.86647
H	-5.03315	2.22818	-0.01368
H	1.25707	2.34389	-2.39825
I	-0.65194	-0.59585	-0.4332
O	-1.1388	-1.4622	-1.93246

10

Energy: -559928.9719157

I	-1.10626	-0.60444	0.74126
C	-0.33464	1.28111	0.14810
C	0.61853	1.40091	-0.84709
C	-0.85136	2.33568	0.87700
C	1.08081	2.68682	-1.12236
H	0.98172	0.53577	-1.39113
C	-0.37533	3.61030	0.57726
H	-1.60290	2.15371	1.64199
C	0.58639	3.78125	-0.41631
H	1.83313	2.82386	-1.89217
H	-0.75734	4.46694	1.12204
H	0.95429	4.77678	-0.64223
O	-2.38899	-0.02621	1.88969
O	0.30072	-0.63323	2.20341
O	-2.49433	-0.34356	-0.93509
O	-0.68741	-1.09280	-1.99167
C	-2.6891	-0.68666	-3.27156
C	-1.8659	-0.72968	-2.00908
H	-2.97706	0.34802	-3.47071
H	-2.1125	-1.08341	-4.10533
H	-3.60644	-1.26072	-3.12929
C	1.31643	-1.59095	1.95348
H	1.92199	-1.62649	2.86441
H	0.89706	-2.60008	1.80231
C	2.16818	-1.21676	0.76017
C	2.02124	-1.87281	-0.46216
C	3.05346	-0.1377	0.85127
C	2.75349	-1.46737	-1.57871
H	1.32205	-2.70088	-0.55309
C	3.78986	0.26454	-0.25586
H	3.14767	0.39029	1.79631
C	3.64126	-0.40163	-1.47435
H	2.61815	-1.97947	-2.52528
H	4.47668	1.10104	-0.17455
H	4.21553	-0.08594	-2.3396

TS7

Energy: -559914.0147311

C	-4.65757	-0.71033	0.74062
C	-3.57630	-1.51189	1.10291
C	-2.27870	-1.08200	0.83898
C	-2.10410	0.14466	0.21207
C	-3.16229	0.95970	-0.15542
C	-4.45544	0.51874	0.11517
H	-5.66763	-1.04686	0.95060
H	-3.74277	-2.46678	1.59002
H	-1.42334	-1.69755	1.11154
H	-2.95716	1.90972	-0.64074
H	-5.30516	1.13376	-0.16161
O	2.20201	1.64506	-0.36496
C	2.08235	2.21330	0.76014
O	1.00625	2.17576	1.42893
C	3.25184	2.98943	1.31631
H	3.21137	3.00794	2.40494
H	4.1903	2.56332	0.96204
H	3.17873	4.01655	0.94868
I	-0.11469	0.77541	-0.20932
O	-0.63712	2.33043	-0.96367
O	-0.10842	-0.2853	-1.90316
C	1.16205	-0.91029	-2.1305
H	1.02017	-1.47281	-3.05832
H	1.94274	-0.15761	-2.28943
C	1.53066	-1.82739	-0.98835
C	2.65002	-1.57444	-0.19454
C	0.68043	-2.89051	-0.6636
C	2.92432	-2.3857	0.90646
H	3.28075	-0.71971	-0.4239
C	0.95271	-3.69646	0.43635
H	-0.2005	-3.06995	-1.2752
C	2.07823	-3.44434	1.22254

H	3.7946	-2.18298	1.52203
H	0.29366	-4.52394	0.67991
H	2.29168	-4.07262	2.08122

11

Energy: -559916.8579251

C	-4.82605	0.54382	-0.29966
C	-3.82830	1.42273	-0.71529
C	-2.49384	1.02782	-0.66073
C	-2.18387	-0.24274	-0.19113
C	-3.16678	-1.13215	0.21738
C	-4.49874	-0.72909	0.16368
H	-5.86583	0.85157	-0.34236
H	-4.08808	2.41021	-1.08231
H	-1.70803	1.70942	-0.98182
H	-2.87770	-2.12120	0.55941
H	-5.28236	-1.40907	0.48135
O	1.94925	-1.37253	0.57995
C	2.53120	-1.84033	-0.48825
O	1.98196	-1.86048	-1.58884
C	3.93274	-2.36276	-0.27340
H	4.37622	-2.64214	-1.22765
H	4.54206	-1.6061	0.22585
H	3.88499	-3.23282	0.38544
I	-0.12926	-0.82788	-0.12629
O	-0.55539	-2.57038	0.11311
O	-0.18191	-0.01568	1.68125
C	0.9689	0.74209	2.08054
H	0.62605	1.23456	2.99692
H	1.80563	0.08148	2.30877
C	1.33991	1.75821	1.02979
C	2.56524	1.69523	0.36591
C	0.40305	2.72799	0.65366
C	2.85563	2.59479	-0.6586
H	3.27519	0.92171	0.64331
C	0.6903	3.62249	-0.37188
H	-0.55466	2.7662	1.1679
C	1.91921	3.55553	-1.0295
H	3.80882	2.53742	-1.17358
H	-0.03827	4.37546	-0.65591
H	2.14406	4.25188	-1.83071

12

Energy: -863728.1299470

C	-0.14544	-2.01047	-2.37027
C	0.02950	-3.38739	-2.49339
C	-0.67643	-1.51713	-1.18903
C	-0.32951	-4.23299	-1.44549
H	0.44773	-3.79949	-3.40570
C	-1.03521	-2.34095	-0.12937
C	-0.86175	-3.71523	-0.26581
H	-0.18775	-5.30419	-1.54622
H	-1.13313	-4.37697	0.55014
H	-1.42552	-1.92415	0.79740
H	0.13611	-1.31937	-3.15996
O	-1.41208	2.86448	-0.66039
C	-0.78952	3.35328	0.34993
O	-0.10377	2.68725	1.15013
H	0.93807	1.22593	1.07555
C	-0.92296	4.85066	0.53515
H	-0.37196	5.34732	-0.2676
H	-1.96952	5.14613	0.44412
H	-0.51672	5.1516	1.49963
O	1.23942	0.39413	0.64926
N	2.64098	0.35045	0.89516
C	2.96352	-0.97895	1.4526
C	3.36272	0.84156	-0.30091
C	4.46283	-0.94236	1.79167
C	2.62103	-2.17493	0.54424
C	2.16338	-1.1154	2.75283
C	4.8493	0.85143	0.08941
C	2.8893	2.28115	-0.53872
C	3.13023	0.04412	-1.59526
C	5.32876	-0.50096	0.61351
H	4.60538	-0.23835	2.6209
H	4.76067	-1.93477	2.14919
H	1.66311	-2.01426	0.04287
H	3.38039	-2.36369	-0.21604
H	2.53312	-3.08094	1.1524
H	2.28547	-0.21713	3.36413
H	1.0999	-1.2536	2.54146
H	2.5205	-1.9813	3.31866
H	5.43426	1.16452	-0.7825
H	4.99068	1.60934	0.87001
H	1.89668	2.29887	-0.99892
H	2.8554	2.83316	0.40485
H	3.57931	2.78524	-1.22196
H	3.67439	-0.90068	-1.62427

H	2.06591	-0.15333	-1.74214
H	3.46118	0.64627	-2.44697
H	5.29625	-1.25093	-0.18517
H	6.37542	-0.43192	0.92654
I	-0.85703	0.57322	-0.8957
O	-0.01015	1.01865	-2.43137
O	-2.72951	0.48233	-1.51084
C	-3.68003	0.92817	-0.52619
H	-3.52243	1.98889	-0.31285
H	-4.64399	0.79748	-1.02437
C	-3.59039	0.08641	0.72428
C	-4.21274	-1.16369	0.77779
C	-2.78311	0.49455	1.79251
C	-4.03684	-1.99318	1.8807
H	-4.82762	-1.4869	-0.05771
C	-2.59412	-0.34288	2.89191
H	-2.28542	1.46306	1.76549
C	-3.22272	-1.58509	2.93768
H	-4.52832	-2.96011	1.91476
H	-1.96045	-0.01892	3.71095
H	-3.08132	-2.23369	3.79617

TS8

Energy: -863725.4608794

C	-0.47050	-2.46099	-1.90558
C	-0.57766	-3.84303	-1.76369
C	-0.89483	-1.66912	-0.85243
C	-1.10121	-4.38593	-0.59190
H	-0.24979	-4.49432	-2.56675
C	-1.42303	-2.17851	0.32535
C	-1.52620	-3.56083	0.44858
H	-1.17717	-5.46322	-0.48681
H	-1.93299	-3.99007	1.35807
H	-1.73668	-1.52144	1.13363
H	-0.05811	-1.99676	-2.79769
O	-0.37171	3.02397	-0.76520
C	0.15015	3.42419	0.29405
O	0.71615	2.67586	1.17020
H	0.93142	1.53821	0.82581
C	0.16043	4.90373	0.60514
H	1.12541	5.30885	0.28705
H	-0.63038	5.4133	0.05663
H	0.06635	5.06659	1.67905
O	1.12404	0.42635	0.43752
N	2.48568	0.18202	0.71905
C	2.61692	-1.03382	1.54254
C	3.33747	0.37315	-0.47091
C	4.08321	-1.07249	2.00703
C	2.22071	-2.35476	0.85698
C	1.71609	-0.83447	2.76688
C	4.78668	0.30753	0.04232
C	3.06184	1.78608	-0.9987
C	3.11669	-0.63094	-1.61619
C	5.07377	-0.95583	0.8507
H	4.2413	-0.23462	2.69709
H	4.2439	-1.99865	2.57115
H	1.31714	-2.21854	0.25962
H	3.00421	-2.75059	0.20886
H	2.00799	-3.11255	1.61824
H	1.87091	0.16398	3.1849
H	0.66216	-0.93566	2.49524
H	1.95379	-1.58444	3.52774
H	5.46167	0.38422	-0.81753
H	4.96008	1.18514	0.67711
H	2.10042	1.83985	-1.5176
H	3.06385	2.49973	-0.16968
H	3.84195	2.06601	-1.71311
H	3.55722	-1.609	-1.41681
H	2.05248	-0.75303	-1.82735
H	3.57596	-0.23811	-2.52869
H	5.01497	-1.84136	0.20717
H	6.09717	-0.92742	1.23852
I	-0.67258	0.41516	-1.01672
O	0.19643	0.42724	-2.60534
O	-2.50755	0.59141	-1.81022
C	-3.33367	1.4078	-0.97157
H	-2.90151	2.41137	-0.86299
H	-4.28226	1.49345	-1.50799
C	-3.53192	0.76441	0.3841
C	-4.37681	-0.34112	0.51639
C	-2.7888	1.18882	1.49038
C	-4.48513	-1.00563	1.73377
H	-4.94113	-0.68265	-0.34709
C	-2.88687	0.51763	2.7094
H	-2.12147	2.04402	1.39748
C	-3.73772	-0.57839	2.83203
H	-5.14821	-1.8597	1.82697
H	-2.30021	0.85437	3.55776

H	-3.82023	-1.09817	3.78112

13

Energy: -720025.5722916

I	0.10898	-0.68439	-0.96345
C	1.34971	-1.28125	0.65358
C	1.96198	-0.36085	1.48702
C	1.47230	-2.65079	0.79605
C	2.74230	-0.85508	2.52981
H	1.87263	0.70994	1.32707
C	2.25673	-3.12450	1.84624
H	0.96337	-3.31288	0.09936
C	2.88565	-2.22930	2.70832
H	3.24312	-0.15852	3.19423
H	2.37453	-4.19348	1.98842
H	3.49520	-2.60466	3.52381
O	-0.34255	-2.34413	-1.56868
O	1.80525	-0.68964	-2.13705
C	2.19297	0.61566	-2.49322
H	2.89779	0.52069	-3.32693
H	1.34408	1.21989	-2.86539
C	2.84421	1.34634	-1.33608
C	2.28593	2.50304	-0.79238
C	3.99369	0.80756	-0.7495
C	2.86108	3.11493	0.3235
H	1.39544	2.93377	-1.24477
C	4.57638	1.41933	0.35343
H	4.40891	-0.1103	-1.15617
C	4.00769	2.57321	0.89662
H	2.41337	4.01239	0.73886
H	5.46812	0.99185	0.80097
H	4.45981	3.04794	1.76166
O	-1.3305	-0.59029	0.51865
N	-2.34237	0.27103	0.03687
C	-3.60913	-0.50533	-0.06805
C	-2.32117	1.50353	0.86908
C	-4.71138	0.46622	-0.51484
C	-3.99713	-1.24214	1.22421
C	-3.40253	-1.54779	-1.16941
C	-3.46372	2.41317	0.39375
C	-0.99349	2.20905	0.57225
C	-2.39363	1.24516	2.38276
C	-4.81327	1.70421	0.36934
H	-4.4944	0.78285	-1.5433
H	-5.65952	-0.08237	-0.53892
H	-3.11356	-1.71944	1.65354
H	-4.44415	-0.58767	1.97442
H	-4.72991	-2.02021	0.99027
H	-3.05072	-1.06617	-2.08832
H	-2.66657	-2.29922	-0.87891
H	-4.35268	-2.04535	-1.38659
H	-3.48993	3.29769	1.04043
H	-3.2296	2.75918	-0.62166
H	-0.14895	1.64559	0.98163
H	-0.86287	2.32345	-0.50935
H	-0.97917	3.20318	1.029
H	-3.4028	1.01336	2.72508
H	-1.73406	0.4144	2.64463
H	-2.06066	2.13752	2.9223
H	-5.12002	1.42841	1.38465
H	-5.58469	2.37982	-0.01352

TS9

Energy: -720010.0365063

I	-0.41187	-0.00078	0.66646
C	-0.35819	2.07590	0.20949
C	-0.10586	2.99448	1.21318
C	-0.56096	2.42659	-1.11297
C	-0.04841	4.34215	0.86399
H	0.04655	2.68010	2.24046
C	-0.50482	3.77923	-1.44314
H	-0.72849	1.65400	-1.85782
C	-0.24700	4.73047	-0.45911
H	0.15224	5.08587	1.62781
H	-0.65983	4.08669	-2.47197
H	-0.20044	5.78153	-0.72391
O	0.00871	-0.55331	-1.04470
O	-2.36955	0.70623	1.26583
C	-3.23893	-0.32617	1.58523
H	-2.70846	-1.18600	2.05357
H	-3.95017	0.02569	2.34786
C	-4.01062	-0.86002	0.39034
C	-4.81098	-1.99805	0.51736
C	-3.93104	-0.22102	-0.84471
C	-5.51965	-2.48987	-0.57247
H	-4.8753	-2.50296	1.47923
C	-4.63863	-0.71444	-1.93981
H	-3.31469	0.66884	-0.92815

C	-5.43366	-1.84801	-1.80771
H	-6.1363	-3.37661	-0.46235
H	-4.5677	-0.2104	-2.89881
H	-5.98313	-2.23302	-2.6608
O	1.85633	-0.01123	1.08136
N	2.46974	-0.6425	0.10095
C	3.37736	0.22002	-0.69404
C	2.78517	-2.0559	0.42066
C	3.8679	-0.5749	-1.91027
C	4.54662	0.72752	0.16906
C	2.57761	1.42357	-1.18727
C	3.30347	-2.74466	-0.84816
C	1.4912	-2.75509	0.85211
C	3.7933	-2.14869	1.57992
C	4.40296	-1.95574	-1.54926
H	3.02192	-0.69229	-2.59894
H	4.62933	0.02456	-2.42157
H	4.15737	1.10653	1.11773
H	5.28612	-0.04778	0.37819
H	5.0576	1.54508	-0.34844
H	1.67236	1.07085	-1.68637
H	2.29949	2.08142	-0.36044
H	3.18663	1.99489	-1.89427
H	3.6465	-3.74865	-0.57433
H	2.45842	-2.86026	-1.53822
H	1.12901	-2.35764	1.80521
H	0.72789	-2.64099	0.07677
H	1.68899	-3.8213	0.99186
H	4.80573	-1.87673	1.27635
H	3.47921	-1.48131	2.38681
H	3.82377	-3.17247	1.965
H	5.28995	-1.87387	-0.90994
H	4.72136	-2.4827	-2.45393

14

Energy: -720022.8288328

I	-1.68030	-0.15519	-1.51819
C	-2.78482	-0.54748	0.26909
C	-3.54879	0.52648	0.68858
C	-2.69570	-1.75003	0.95485
C	-4.25917	0.39116	1.88124
H	-3.55981	1.42805	0.07612
C	-3.42103	-1.86282	2.14037
H	-2.05281	-2.53884	0.58130
C	-4.19458	-0.79869	2.60298
H	-4.86622	1.21512	2.24313
H	-3.37597	-2.78809	2.70624
H	-4.75203	-0.90022	3.52885
O	-0.45685	-2.01636	-0.73347
C	0.63668	-2.18555	-1.54115
C	1.95273	-2.18980	-0.77519
H	0.72914	-1.39681	-2.33327
C	2.00357	-2.79335	0.48542
C	3.12384	-1.64202	-1.30037
C	3.19699	-2.85547	1.19804
H	1.08043	-3.19846	0.88968
C	4.32218	-1.69017	-0.58499
H	3.09845	-1.17388	-2.28346
C	4.36228	-2.30016	0.66621
H	3.22178	-3.33297	2.17327
H	5.22329	-1.25451	-1.00749
H	5.29316	-2.34417	1.22286
O	-0.48921	1.49892	0.05141
N	0.67577	1.59864	0.40036
C	1.58191	2.37929	-0.51765
C	1.04525	1.04875	1.74763
C	3.04106	1.95032	-0.31965
C	1.35915	3.86105	-0.17855
C	1.12474	2.08801	-1.9474
C	2.52619	0.65826	1.73507
C	0.15008	-0.16659	1.99806
C	0.73976	2.14838	2.77838
C	3.43074	1.73063	1.13785
H	3.20231	1.01033	-0.85886
H	3.66891	2.71217	-0.79352
H	0.29344	4.09916	-0.22315
H	1.74626	4.12171	0.80912
H	1.88325	4.46755	-0.92189
H	1.25258	1.02352	-2.17148
H	0.07721	2.36265	-2.10333
H	1.76182	2.65703	-2.62908
H	2.80822	0.4256	2.76726
H	2.63599	-0.2642	1.15535
H	-0.87111	0.13668	2.24146
H	0.11658	-0.82812	1.12272
H	0.56558	-0.71935	2.84571
H	1.42447	2.99629	2.71095
H	-0.28614	2.50411	2.65275

H	0.83372	1.71083	3.77584
H	3.37711	2.6652	1.70905
H	4.47017	1.39328	1.18405
O	-2.48209	1.51474	-1.81907
H	0.59259	-3.13451	-2.11271

TS10

Energy: -720017.1115877

I	-1.86110	-0.63779	-1.57900
C	-2.70830	-0.89564	0.36889
C	-3.71559	0.00592	0.66063
C	-2.27136	-1.87733	1.24453
C	-4.32066	-0.07801	1.91386
H	-3.98884	0.74328	-0.09453
C	-2.89656	-1.94491	2.49022
H	-1.45084	-2.53067	0.96850
C	-3.91257	-1.05049	2.82468
H	-5.11435	0.61463	2.17656
H	-2.58233	-2.70134	3.20292
H	-4.39003	-1.11547	3.79733
O	0.17772	-1.88555	-0.34879
C	1.16350	-1.20323	-0.91525
C	2.57722	-1.64228	-0.55671
H	1.16486	-0.07822	-0.60623
C	2.7749	-2.5165	0.50976
C	3.6892	-1.12526	-1.22742
C	4.0654	-2.85264	0.91515
H	1.89062	-2.92185	0.99164
C	4.97919	-1.45589	-0.82382
H	3.53812	-0.46929	-2.08434
C	5.16996	-2.3181	0.25613
H	4.21142	-3.53753	1.74533
H	5.83602	-1.05256	-1.35532
H	6.17481	-2.58022	0.57199
O	-0.84473	1.34321	0.39953
N	0.31116	1.64284	0.44867
C	0.83048	2.59598	-0.62424
C	1.05776	1.29485	1.73546
C	2.35409	2.47338	-0.71413
C	0.37341	3.99388	-0.16693
C	0.15277	2.2431	-1.94681
C	2.56487	1.2676	1.46997
C	0.51679	-0.05245	2.20937
C	0.67673	2.39279	2.74677
C	3.06128	2.4481	0.63819
H	2.59445	1.55095	-1.25388
H	2.70348	3.30986	-1.32844
H	-0.7072	4.00286	-0.00502
H	0.88065	4.33658	0.73536
H	0.60606	4.69271	-0.97348
H	0.54132	1.29465	-2.32475
H	-0.93887	2.17094	-1.8679
H	0.40891	3.02799	-2.66318
H	3.05253	1.25162	2.4503
H	2.82366	0.32918	0.96932
H	-0.48175	0.05698	2.63969
H	0.45683	-0.78873	1.39669
H	1.19605	-0.42074	2.98328
H	1.17914	3.34387	2.56512
H	-0.40536	2.54544	2.76109
H	0.98242	2.03068	3.73158
H	2.92075	3.39603	1.17083
H	4.1371	2.34095	0.47553
O	-2.95681	0.83621	-2.08177
H	1.11595	-1.10801	-2.0315

15

Energy: -720068.9966873

I	-2.01354	-0.58123	-1.53731
C	-2.66763	-0.71151	0.49168
C	-3.26717	0.43796	0.97202
C	-2.47832	-1.85996	1.24015
C	-3.70404	0.42838	2.29351
H	-3.34294	1.30534	0.31983
C	-2.91714	-1.84343	2.56581
H	-1.99150	-2.73398	0.82055
C	-3.52627	-0.70560	3.08744
H	-4.17521	1.31353	2.70844
H	-2.78161	-2.72460	3.18474
H	-3.86627	-0.70176	4.11810
O	0.08186	-2.75214	-0.52576
C	1.01624	-2.26026	-1.12202
C	2.38302	-2.14566	-0.57827
H	0.55287	0.58117	-0.24696
C	2.67308	-2.63739	0.69893
C	3.36201	-1.48657	-1.32391
C	3.9431	-2.45777	1.22858
H	1.88738	-3.14002	1.25436

C	4.63294	-1.30071	-0.78753
H	3.12138	-1.11267	-2.31675
C	4.9185	-1.78514	0.48758
H	4.17777	-2.8311	2.2196
H	5.39552	-0.78115	-1.35776
H	5.90797	-1.63894	0.90883
O	-0.90832	1.7357	0.37094
N	0.40508	1.50231	0.22007
C	1.04222	2.48589	-0.7765
C	1.09091	1.30279	1.57954
C	2.54651	2.20787	-0.86334
C	0.71446	3.91305	-0.35459
C	0.34891	2.17201	-2.10243
C	2.59008	1.07892	1.36192
C	0.42396	0.05217	2.15301
C	0.78881	2.4889	2.48885
C	3.24195	2.1544	0.49499
H	2.69608	1.24011	-1.36378
H	2.99467	2.96783	-1.51316
H	-0.33255	3.94365	-0.04815
H	1.34618	4.27444	0.4585
H	0.85978	4.5768	-1.21069
H	0.65202	1.17774	-2.46071
H	-0.74015	2.18124	-1.9742
H	0.64937	2.8987	-2.8617
H	3.06946	1.01749	2.34567
H	2.73905	0.10315	0.88176
H	-0.63003	0.2513	2.35857
H	0.47871	-0.7866	1.44579
H	0.93491	-0.24787	3.07281
H	1.44788	3.34137	2.31724
H	-0.2494	2.78441	2.32634
H	0.91487	2.17489	3.52873
H	3.19864	3.13122	0.98915
H	4.3031	1.92126	0.35776
O	-2.81695	1.073	-2.04591
H	0.8746	-1.82048	-2.13262

12.3 Cycle 2: TEMPO Oxidation

TS11

Energy: -512803.5925873

C	3.28330	2.80047	-0.53995
C	3.02826	1.56054	-1.12114
C	1.81172	0.92290	-0.89605
C	0.86248	1.53456	-0.08063
C	1.10961	2.77431	0.50419
C	2.32701	3.40847	0.27038
H	4.23164	3.29662	-0.71939
H	3.77545	1.08384	-1.74743
H	1.61410	-0.04895	-1.34136
H	0.36219	3.24311	1.13700
H	2.52809	4.37442	0.72159
I	-0.99737	0.52576	0.27402
O	-0.27144	-0.44561	1.88757
O	-3.19059	-0.70237	0.38092
C	-3.65456	-0.08139	-0.62105
O	-3.02230	0.82326	-1.23470
C	-5.05602	-0.42863	-1.08478
H	-5.21981	-0.07573	-2.10251
H	-5.21345	-1.50582	-1.0147
H	-5.77362	0.05835	-0.41886
C	-0.09645	-1.84514	1.65566
H	-1.03117	-2.2984	1.30352
C	1.03681	-2.12163	0.70105
C	0.79969	-2.6823	-0.55421
C	2.33806	-1.74794	1.04852
C	1.8497	-2.87292	-1.45231
H	-0.21393	-2.96271	-0.83036
C	3.38707	-1.93704	0.1565
H	2.51623	-1.29092	2.0182
C	3.14359	-2.49999	-1.09695
H	1.65621	-3.31009	-2.42648
H	4.39413	-1.64238	0.43379
H	3.96231	-2.64978	-1.79364
H	0.12504	-2.24451	2.65167

16

Energy: -512804.7724689

O	-0.45311	0.38466	-0.30995
C	1.98777	1.99370	-0.67770
C	2.89952	3.04695	-0.64814
C	2.33635	0.77849	-0.09990
C	4.14806	2.88097	-0.05357
H	2.63281	3.99934	-1.09514
C	3.57767	0.60556	0.50259
C	4.48886	1.65997	0.52063
H	4.85441	3.70437	-0.03541

H	5.45757	1.52769	0.99135
I	1.01832	-0.94147	-0.13488
C	-1.17115	0.61870	0.91094
H	-0.59298	1.28704	1.56277
C	-2.47489	1.23388	0.49403
C	-3.37592	0.44725	-0.23027
C	-2.78899	2.55861	0.78672
C	-4.58726	0.98503	-0.64585
H	-3.1014	-0.58025	-0.45681
C	-4.00774	3.09504	0.37583
H	-2.08065	3.16947	1.34001
C	-4.9052	2.30912	-0.34038
H	-5.28772	0.37456	-1.20652
H	-4.25307	4.12561	0.61106
H	-5.85357	2.72705	-0.6628
H	3.84382	-0.3409	0.96923
H	1.01191	2.11636	-1.13466
O	-0.93799	-2.16957	-0.06574
C	-0.57214	-3.40912	0.05987
C	-1.71538	-4.40111	0.1376
O	0.60503	-3.76543	0.10578
H	-2.38751	-4.124	0.95245
H	-2.28992	-4.36162	-0.79071
H	-1.32522	-5.40595	0.29255
H	-1.348	-0.33641	1.41619

TS12

Energy: -816615.7120671

C	1.84566	-1.73215	-0.43505
C	2.23363	-3.00995	-0.82964
C	1.20762	-0.91079	-1.35854
C	1.97961	-3.45105	-2.12574
H	2.73295	-3.65773	-0.1165
C	0.95458	-1.33389	-2.6601
C	1.34314	-2.61559	-3.04067
H	2.27806	-4.45047	-2.42487
H	1.14552	-2.95951	-4.05027
H	0.44736	-0.68175	-3.36377
H	2.0344	-1.39392	0.57899
O	-0.65722	3.28201	-0.08296
C	-1.83732	3.40597	-0.48197
O	-2.59637	2.45823	-0.86894
H	-2.17789	1.22339	-0.72163
C	-2.46594	4.78698	-0.486
H	-3.1327	4.86613	0.37749
H	-1.70013	5.55832	-0.4178
H	-3.07218	4.91731	-1.38348
O	-1.73817	0.21206	-0.59087
N	-2.62001	-0.48733	0.26179
C	-2.94953	-1.79288	-0.34105
C	-2.21939	-0.40039	1.67901
C	-4.0664	-2.40234	0.52304
C	-1.76861	-2.76764	-0.48815
C	-3.51224	-1.50719	-1.73758
C	-3.35614	-1.04506	2.49091
C	-2.1529	1.08809	2.03954
C	-0.86979	-1.05705	2.03524
C	-3.71227	-2.4476	2.0064
H	-4.96717	-1.79042	0.39141
H	-4.29156	-3.40452	0.14074
H	-0.9151	-2.25757	-0.93924
H	-1.44926	-3.20549	0.45925
H	-2.05666	-3.5895	-1.15176
H	-4.25512	-0.70731	-1.68323
H	-2.7191	-1.19475	-2.41986
H	-3.98457	-2.40988	-2.1378
H	-3.06301	-1.05449	3.54716
H	-4.24003	-0.40202	2.40131
H	-1.28192	1.58374	1.60194
H	-3.04693	1.59872	1.67234
H	-2.09602	1.19768	3.12722
H	-0.94068	-2.13912	2.15909
H	-0.12241	-0.85898	1.26524
H	-0.49533	-0.63897	2.97571
H	-2.87779	-3.13777	2.17866
H	-4.55684	-2.8389	2.58262
I	0.52967	1.01409	-0.78417
O	2.36656	1.83463	-1.04171
C	2.97433	2.22832	0.1879
H	2.31707	2.9031	0.75249
C	3.36818	1.03958	1.02937
C	2.65817	0.70593	2.18447
C	4.40838	0.20775	0.60537
C	2.98327	-0.44122	2.90864
H	1.83983	1.34351	2.51241
C	4.73849	-0.93436	1.32655

H	4.9483	0.45822	-0.3038
C	4.02454	-1.26133	2.48014
H	2.42333	-0.69281	3.80369
H	5.54949	-1.57239	0.99031
H	4.28158	-2.152	3.04462
H	3.85942	2.79663	-0.11691

17

Energy: -672918.0887696

O	-1.95881	-0.96336	-0.52231
C	-2.05543	1.89432	-0.65765
C	-2.55905	3.18905	-0.54551
C	-0.76617	1.65436	-0.20699
C	-1.78019	4.20488	0.00134
H	-3.56704	3.39591	-0.88945
C	0.03641	2.64364	0.34290
C	-0.48897	3.93166	0.44225
H	-2.18016	5.21000	0.08425
H	0.12135	4.72061	0.86936
I	0.02362	-0.34414	-0.37106
C	-2.67040	-0.97966	0.68590
H	-2.51299	-0.04730	1.25891
C	-4.15575	-1.12130	0.43095
C	-4.65776	-1.25013	-0.86181
C	-5.04351	-1.11441	1.50993
C	-6.03137	-1.3682	-1.07256
H	-3.96004	-1.26249	-1.69191
C	-6.41201	-1.23338	1.30048
H	-4.65617	-1.01318	2.52152
C	-6.91118	-1.35997	0.00436
H	-6.41312	-1.46949	-2.08389
H	-7.09145	-1.22597	2.14711
H	-7.97976	-1.45242	-0.16123
H	1.04528	2.41784	0.66512
H	-2.65131	1.08626	-1.06827
O	1.91736	0.60398	-0.21581
N	2.80918	-0.45597	0.0328
C	3.73342	-0.57513	-1.1255
C	3.38761	-0.27509	1.38984
C	4.71578	-1.71525	-0.81918
C	4.46987	0.72874	-1.47791
C	2.88716	-0.97513	-2.3386
C	4.37787	-1.42413	1.63073
C	2.23526	-0.40701	2.39132
C	4.05407	1.09423	1.61471
C	5.41987	-1.54926	0.52371
H	4.15418	-2.65841	-0.80971
H	5.43953	-1.77642	-1.63988
H	3.77039	1.56649	-1.42683
H	5.31514	0.93431	-0.81975
H	4.85847	0.66506	-2.4989
H	2.28407	-1.85981	-2.11012
H	2.22511	-0.15837	-2.63697
H	3.53978	-1.21384	-3.18353
H	4.85345	-1.2714	2.60635
H	3.8087	-2.36088	1.68898
H	1.53943	0.43225	2.30068
H	1.6871	-1.33884	2.22448
H	2.63028	-0.41395	3.4114
H	5.06055	1.15324	1.1985
H	3.44537	1.8807	1.16163
H	4.13173	1.29253	2.68809
H	6.07096	-0.66774	0.50835
H	6.07031	-2.40842	0.71585
H	-2.33657	-1.80692	1.33521

TS13

Energy: -841282.3606119

C	2.56353	-1.88305	-1.58388
C	3.40758	-2.98889	-1.4962
C	1.53826	-1.75147	-0.65545
C	3.20806	-3.94545	-0.50393
H	4.21837	-3.10122	-2.20856
C	1.3238	-2.69433	0.34321
C	2.16512	-3.80249	0.40855
H	3.86629	-4.80572	-0.44194
H	2.00897	-4.54939	1.17998
C	3.34091	1.66814	-0.40874
C	2.86639	1.32246	0.9938
C	3.162	0.10385	1.60624
C	1.98675	2.20475	1.63088
C	2.57862	-0.23379	2.82692
H	3.83177	-0.60317	1.12412
C	1.40972	1.87339	2.85171
H	1.7334	3.13937	1.13607
C	1.70208	0.64881	3.45174
H	2.81067	-1.18958	3.28706

H	0.72363	2.56426	3.33176
H	1.24625	0.38463	4.40102
H	0.51347	-2.56631	1.05427
H	2.71699	-1.12246	-2.34307
O	-1.13166	2.20063	-0.69975
C	-0.45448	3.22305	-0.96295
O	0.78028	3.25591	-1.26085
O	2.33004	1.32804	-1.36362
H	1.67052	2.15034	-1.40138
H	3.47167	2.75856	-0.45131
C	-1.15289	4.57441	-0.93537
H	-1.11923	5.01205	-1.93608
H	-2.18706	4.47147	-0.60962
H	-0.61052	5.24955	-0.2695
C	4.63976	0.99945	-0.82529
H	4.92414	1.35229	-1.81851
H	5.44207	1.23151	-0.11982
H	4.52078	-0.08642	-0.87399
I	0.30761	-0.02566	-0.70871
O	-1.31859	-1.28854	-0.23017
N	-2.4469	-0.57258	0.03085
C	-3.44829	-0.75978	-1.04045
C	-2.77159	-0.59408	1.47156
C	-4.63602	0.1574	-0.71843
C	-3.88352	-2.22547	-1.19462
C	-2.80934	-0.28652	-2.35129
C	-3.99663	0.30828	1.6786
C	-1.58495	0.02745	2.21702
C	-3.01391	-2.01507	2.00457
C	-5.13691	0.00021	0.71369
H	-4.30893	1.19389	-0.8673
H	-5.43162	-0.04891	-1.44299
H	-3.00127	-2.87041	-1.23375
H	-4.52376	-2.56243	-0.37667
H	-4.44155	-2.34939	-2.12783
H	-2.37733	0.71065	-2.21867
H	-2.03241	-0.98	-2.6856
H	-3.57624	-0.24099	-3.13015
H	-4.31923	0.20924	2.7212
H	-3.67815	1.34692	1.52609
H	-0.70843	-0.62775	2.21127
H	-1.30982	0.9832	1.759
H	-1.86164	0.20243	3.26155
H	-3.96749	-2.43064	1.67236
H	-2.21265	-2.67858	1.66609
H	-3.01559	-2.00657	3.09898
H	-5.52398	-1.0121	0.88072
H	-5.97216	0.68382	0.89458

12.4 Cycle 2_SI: TEMPO Oxidation

19

Energy: -742794.5158055

C	-0.74723	2.51451	0.94366
C	-0.35818	3.85144	0.97126
C	-0.86145	1.86457	-0.28334
C	-0.09264	4.52723	-0.21762
H	-0.26269	4.36441	1.92260
C	-0.60772	2.53675	-1.47659
C	-0.22067	3.87417	-1.44095
H	0.21426	5.56774	-0.19058
H	-0.01586	4.40213	-2.36641
H	-0.69391	2.01923	-2.42761
H	-0.96690	1.98194	1.86401
O	-3.27524	0.31746	-0.26696
C	-3.75995	0.53525	0.97303
C	-5.24795	0.76041	0.91294
O	-3.08224	0.54675	1.96829
H	-5.47709	1.54168	0.18669
H	-5.72693	-0.16198	0.57829
H	-5.60561	1.0364	1.90286
I	-1.33087	-0.22385	-0.34973
O	-2.54266	-2.26654	-0.36116
C	-1.66529	-3.16017	-0.62048
O	-0.45582	-2.9175	-0.80608
H	0.98501	-1.71999	-0.90732
C	-2.1754	-4.58551	-0.69893
H	-2.65776	-4.84854	0.24483
H	-2.93211	-4.65386	-1.48346
H	-1.35466	-5.27025	-0.90724
O	1.25986	-0.80316	-0.71151
N	2.59044	-0.91072	-0.21424
C	3.3949	0.14955	-0.85714
C	2.56228	-1.02029	1.2628
C	4.83476	-0.02771	-0.34888
C	2.89012	1.58358	-0.61683
C	3.36561	-0.12881	-2.36362

C	4.02348	-1.17468	1.71288
C	1.80309	-2.31119	1.58924
C	1.87704	0.1464	1.9944
C	4.928	-0.06962	1.17403
H	5.225	-0.9695	-0.75431
H	5.44617	0.78363	-0.75985
H	1.80372	1.62855	-0.72374
H	3.1549	1.97249	0.3676
H	3.3287	2.25306	-1.36359
H	3.6054	-1.17807	-2.55463
H	2.3785	0.08434	-2.77944
H	4.10168	0.50163	-2.87172
H	4.04499	-1.20523	2.80808
H	4.3902	-2.14326	1.35119
H	0.73719	-2.21978	1.36464
H	2.20022	-3.14182	0.9992
H	1.91238	-2.54624	2.65208
H	2.50853	1.03184	2.0816
H	0.96191	0.43539	1.47383
H	1.60126	-0.16759	3.00561
H	4.64557	0.89941	1.60156
H	5.96316	-0.25005	1.48054

TS14

Energy: -742793.5806156

C	-1.06143	2.58761	0.82890
C	-0.83732	3.96090	0.77333
C	-1.06019	1.85472	-0.35564
C	-0.61596	4.58511	-0.45217
H	-0.83447	4.54204	1.68946
C	-0.84711	2.47036	-1.58680
C	-0.62249	3.84398	-1.63122
H	-0.43710	5.65476	-0.48858
H	-0.45014	4.33171	-2.58479
H	-0.83850	1.88437	-2.50094
H	-1.24333	2.09070	1.77636
O	-3.27494	-0.12409	-0.17791
C	-3.76712	0.15865	1.04544
C	-5.27031	0.06369	1.03681
O	-3.08364	0.44654	1.99496
H	-5.67645	0.69354	0.24376
H	-5.55593	-0.96866	0.82533
H	-5.65173	0.37281	2.00792
I	-1.25602	-0.26889	-0.30147
O	-1.85666	-2.65394	-0.22897
C	-0.95371	-3.32227	-0.81993
O	0.13584	-2.86411	-1.24547
H	0.956	-1.54344	-1.01523
C	-1.23354	-4.80254	-1.00998
H	-1.29883	-5.27839	-0.02837
H	-2.20095	-4.93047	-1.49933
H	-0.44389	-5.27003	-1.59632
O	1.1991	-0.59686	-0.75661
N	2.48895	-0.6625	-0.16882
C	3.29367	0.45003	-0.71357
C	2.38847	-0.84923	1.2955
C	4.71335	0.27079	-0.15058
C	2.74269	1.85586	-0.41725
C	3.34221	0.26041	-2.2333
C	3.83099	-1.01366	1.80177
C	1.6336	-2.16441	1.5269
C	1.66868	0.27877	2.06221
C	4.7423	0.13234	1.36904
H	5.14099	-0.63506	-0.59761
H	5.32455	1.11835	-0.48076
H	1.67073	1.89622	-0.63001
H	2.90139	2.17128	0.61516
H	3.24021	2.58352	-1.06618
H	3.60541	-0.77309	-2.47252
H	2.37415	0.48525	-2.68545
H	4.0938	0.92846	-2.6651
H	3.8041	-1.10559	2.89349
H	4.22516	-1.95597	1.40213
H	0.56041	-2.06134	1.34248
H	2.01626	-2.94395	0.86351
H	1.76242	-2.47885	2.56699
H	2.33271	1.10369	2.3265
H	0.84899	0.69279	1.47213
H	1.24522	-0.11693	2.99021
H	4.43121	1.07055	1.84311
H	5.76594	-0.05625	1.70767

20

Energy: -599104.6662064

O	2.80167	-1.41682	-0.18763
C	3.22382	1.27639	0.63339
C	3.93344	2.47479	0.66675
C	1.96089	1.27842	0.05817

C	3.38064	3.63817	0.13867
H	4.92310	2.49259	1.11104
C	1.38378	2.42272	-0.47519
C	2.11048	3.61110	-0.42865
H	3.94057	4.56688	0.17027
H	1.67592	4.51551	-0.84123
I	0.80798	-0.52192	0.00715
H	0.38481	2.39298	-0.89382
H	3.64961	0.36106	1.02374
O	-0.83634	0.76255	0.18496
N	-1.95679	-0.07963	0.01453
C	-2.71057	-0.11604	1.29520
C	-2.66485	0.3298	-1.22594
C	-3.94039	-1.00978	1.07838
C	-3.10506	1.27008	1.83255
C	-1.80183	-0.78253	2.33358
C	-3.89787	-0.57447	-1.37245
C	-1.71873	0.05079	-2.39887
C	-3.05013	1.81903	-1.26949
C	-4.77652	-0.5877	-0.12565
H	-3.59287	-2.03942	0.925
H	-4.5376	-1.00112	1.99718
H	-2.26133	1.95682	1.73083
H	-3.96961	1.69668	1.32215
H	-3.35827	1.19051	2.89415
H	-1.4259	-1.73957	1.95797
H	-0.95428	-0.13803	2.58118
H	-2.36632	-0.97093	3.25143
H	-4.46288	-0.24518	-2.25192
H	-3.55076	-1.5963	-1.57172
H	-0.85978	0.72805	-2.3841
H	-1.35532	-0.98028	-2.36197
H	-2.24929	0.19831	-3.34404
H	-3.95001	2.04459	-0.69581
H	-2.22898	2.42573	-0.879
H	-3.23845	2.11816	-2.30511
H	-5.21987	0.40048	0.04257
H	-5.61241	-1.28058	-0.26384
C	2.67862	-2.722	-0.22884
C	3.9953	-3.452	-0.37411
H	4.4863	-3.13446	-1.29633
H	4.65228	-3.18884	0.45748
H	3.81927	-4.52612	-0.39093
O	1.601	-3.29438	-0.1587

TS15

Energy: -673066.1682533

O	-3.10593	0.68824	0.16381
C	-0.17996	3.33326	0.78508
C	0.40076	4.59182	0.92648
C	0.27095	2.49506	-0.22861
C	1.41968	4.99495	0.06741
H	0.05552	5.25484	1.71298
C	1.27639	2.88794	-1.10643
C	1.85685	4.14432	-0.94513
H	1.87078	5.97477	0.18378
H	2.64668	4.45955	-1.61938
I	-0.57199	0.54745	-0.42391
H	1.61560	2.22021	-1.89214
H	-0.97315	3.01062	1.45195
O	1.68961	-0.11595	-0.85378
N	2.17466	-1.05685	-0.13872
C	2.06808	-2.41871	-0.72772
C	3.06587	-0.62743	0.96944
C	2.41007	-3.47369	0.33185
C	3.02407	-2.50023	-1.92753
C	0.62616	-2.62542	-1.20051
C	3.343	-1.80889	1.90767
C	2.37638	0.49299	1.75297
C	4.3637	-0.08457	0.34908
C	3.63034	-3.11048	1.16924
H	1.54778	-3.5939	0.99941
H	2.54955	-4.4281	-0.18607
H	2.83419	-1.66255	-2.60309
H	4.07216	-2.46949	-1.61799
H	2.85385	-3.43423	-2.47091
H	-0.10384	-2.45376	-0.40244
H	0.3809	-1.97755	-2.04501
H	0.51682	-3.66209	-1.53049
H	4.17658	-1.52479	2.55842
H	2.46809	-1.95824	2.55281
H	2.25868	1.39324	1.14624
H	1.3939	0.17285	2.11159
H	2.995	0.73811	2.62072
H	4.93875	-0.87159	-0.14555
H	4.11861	0.68578	-0.38688
H	4.98903	0.36108	1.1282
H	4.52268	-3.01349	0.53965
H	3.84106	-3.90763	1.88776

C	-3.29921	-0.55875	0.0426
C	-4.73142	-1.05513	0.34679
O	-2.42188	-1.38676	-0.30045
C	-4.81888	-2.56993	0.18236
H	-5.83701	-2.91381	0.39688
H	-4.55372	-2.86519	-0.83561
H	-4.12662	-3.07454	0.86098
C	-5.68728	-0.35308	-0.62529
H	-6.72269	-0.64562	-0.41809
H	-5.59658	0.73082	-0.52555
H	-5.45852	-0.62427	-1.66122
C	-5.07864	-0.64937	1.78391
H	-6.10502	-0.9471	2.02568
H	-4.40744	-1.13525	2.50019
H	-4.98443	0.43225	1.90281

13. References

1. Martin, M. L.; Boyer, A. Controlling Selectivity in the Synthesis of Z-α, β-Unsaturated Amidines by Tuning the N-Sulfonyl Group in a Rhodium (II) Catalyzed 1, 2-H Shift. *Eur. J. Org. Chem.* **2021**, *2021* (43), 5857–5861.
2. Jung, H. H.; Floreancig, P. E. Mechanistic analysis of oxidative C–H cleavages using inter- and intramolecular kinetic isotope effects. *Tetrahedron* **2009**, *65* (52), 10830–10836.
3. Ge, J.-J.; Yao, C.-Z.; Wang, M.-M.; Zheng, H.-X.; Kang, Y.-B.; Li, Y. Transition-metal-free deacylative cleavage of unstrained C (sp3)–C (sp2) bonds: cyanide-free access to aryl and aliphatic nitriles from ketones and aldehydes. *Org. Lett.* **2016**, *18* (2), 228–231.
4. Chahdoura, F.; Mallet-Ladeira, S.; Gómez, M. Palladium nanoparticles in glycerol: a clear-cut catalyst for one-pot multi-step processes applied in the synthesis of heterocyclic compounds. *Org. Chem. Front.* **2015**, *2* (4), 312–318.
5. Lucchetti, N.; Tkacheva, A.; Fantasia, S.; Muñiz, K. Radical C− H-Amination of Heteroarenes using Dual Initiation by Visible Light and Iodine. *Adv. Synth. Catal.* **2018**, *360* (20), 3889–3893.
6. Maity, A.; Hyun, S.-M.; Powers, D. C. Oxidase catalysis via aerobically generated hypervalent iodine intermediates. *Nat. Chem.* **2018**, *10* (2), 200–204.
7. Tsujiyama, S.; Suzuki, K.; Guthrie, D. B.; Gibney, H. M. Preparation of benzocyclobutenone derivatives based on an efficient generation of benzynes. *Org. Synth.* **2007**, *84*, 272—284.
8. Uyanik, M.; Yasui, T.; Ishihara, K. Enantioselective Kita oxidative spirolactonization catalyzed by in situ generated chiral hypervalent iodine (III) species. *Angew. Chem. Int. Ed.* **2010**, *49* (12), 2175–2177.
9. Pluta, R.; Krach, P. E.; Cavallo, L.; Falivene, L.; Rueping, M. Metal-free catalytic asymmetric fluorination of keto esters using a combination of hydrogen fluoride (HF) and oxidant: experiment and computation. *ACS Catal.* **2018**, *8* (3), 2582–2588.
10. Shimogaki, M.; Fujita, M.; Sugimura, T. Enantioselective Oxidation of Alkenylbenzoates Catalyzed by Chiral Hypervalent Iodine (III) To Yield 4-Hydroxyisochroman-1-ones. *Eur. J. Org. Chem.* **2013**, *2013* (31), 7128–7138.
11. Hokamp, T.; Wirth, T. Hypervalent iodine (III)-catalysed enantioselective α-acetoxylation of ketones. *Chem. Eur. J.* **2020**, *26* (46), 10417–10421.
12. De Mico, A.; Margarita, R.; Parlanti, L.; Vescovi, A.; Piancatelli, G. A Versatile and Highly Selective Hypervalent Iodine (III)/2,2,6,6-Tetramethyl-1-piperidinyloxyl-Mediated Oxidation of Alcohols to Carbonyl Compounds. *J. Org. Chem.* **1997**, *62* (20), 6974–6977.
13. Burés, J. Variable Time Normalization Analysis: General Graphical Elucidation of Reaction Orders from Concentration Profiles. *Angew. Chem. Int. Ed.* **2016**, *55* (52), 16084–16087.
14. Whitaker, D.; Burés, J.; Larrosa, I. Ag(I)-Catalyzed C–H Activation: The Role of the Ag(I) Salt in Pd/Ag-Mediated C–H Arylation of Electron-Deficient Arenes. *J. Am. Chem. Soc.* **2016**, *138* (27), 8384–8387.
15. Zhao, Y.; Truhlar, D. G. The M06 suite of density functionals for main group thermochemistry, thermochemical kinetics, noncovalent interactions, excited states, and transition elements: two new functionals and systematic testing of four M06-class functionals and 12 other functionals. *Theor. Chem. Acc.* **2008**, *120* (1), 215–241.

16. Zhao, Y.; Truhlar, D. G. Density functionals with broad applicability in chemistry. *Acc. Chem. Res.* **2008**, *41* (2), 157–167.
17. Bergner, A.; Dolg, M.; Küchle, W.; Stoll, H.; Preuß, H. Ab initio energy-adjusted pseudopotentials for elements of groups 13–17. *Mol. Phys.* **1993**, *80* (6), 1431–1441.
18. Weigend, F.; Furche, F.; Ahlrichs, R. Gaussian basis sets of quadruple zeta valence quality for atoms H–Kr. *J. Chem. Phys.* **2003**, *119* (24), 12753–12762.
19. Tomasi, J.; Mennucci, B.; Cancès, E. The IEF version of the PCM solvation method: an overview of a new method addressed to study molecular solutes at the QM ab initio level. *J. Mol. Struct.* **1999**, *464* (1-3), 211–226.
20. Johnson, E. R.; Keinan, S.; Mori-Sánchez, P.; Contreras-García, J.; Cohen, A. J.; Yang, W. Revealing noncovalent interactions. *J. Am. Chem. Soc.* **2010**, *132* (18), 6498–6506.
21. Robert Paton, Pymol, https://gist.github.com/bobbypaton, accessed May 2025.
22. Claude Legault, CYLview, https://www.cylview.org/, accessed May 2025.
23. Farshadfar, K.; Chipman, A.; Yates, B. F.; Ariafard, A. DFT mechanistic investigation into BF_3-catalyzed alcohol oxidation by a hypervalent iodine (III) compound. *ACS Catal.* **2019**, *9* (7), 6510–6521.
24. Ganji, B.; Ariafard, A. DFT mechanistic investigation into phenol dearomatization mediated by an iodine (iii) reagent. *Org. Biomol. Chem.* **2019**, *17* (14), 3521–3528.
